# Nephroplex: a kidney-focused NGS panel highlights the challenges of *PKD1* sequencing and identifies a founder *BBS4* mutation

**DOI:** 10.1007/s40620-021-01048-4

**Published:** 2021-05-08

**Authors:** Miriam Zacchia, Francesca Del Vecchio Blanco, Francesco Trepiccione, Giancarlo Blasio, Annalaura Torella, Andrea Melluso, Giovanna Capolongo, Rosa Maria Pollastro, Giulio Piluso, Valentina Di Iorio, Francesca Simonelli, Davide Viggiano, Alessandra Perna, Vincenzo Nigro, Giovambattista Capasso

**Affiliations:** 1 Department of Translational Medical Sciences, University of Campania “Luigi Vanvitelli”, Naples, Italy; 2 Department of Precision Medicine, University of Campania “Luigi Vanvitelli”, Naples, Italy; 3 Biogem Scarl, 83031 Ariano Irpino, AV Italy; 4 Eye Clinic, Multidisciplinary Department of Medical, Surgical and Dental Sciences, University of Campania “Luigi Vanvitelli”, Naples, Italy; 5 Telethon Institute of Genetics and Medicine (TIGEM), Pozzuoli, NA Italy; 6 Section of Nephrology, Università degli studi di Napoli Federico II, Via Pansini 5, 80131 Naples, Italy

**Keywords:** Gene-panel, Inherited kidney disease, NGS, Bardet-Biedl syndrome, ADPKD

## Abstract

**Background:**

Genetic testing of patients with inherited kidney diseases has emerged as a tool of clinical utility by improving the patients’ diagnosis, prognosis, surveillance and therapy.

**Methods:**

The present study applied a Next Generation Sequencing (NGS)-based panel, named NephroPlex, testing 115 genes causing renal diseases, to 119 individuals, including 107 probands and 12 relatives. Thirty-five (poly)cystic and 72 non (poly)cystic individuals were enrolled. The latter subgroup of patients included Bardet-Biedl syndrome (BBS) patients, as major components.

**Results:**

Disease-causing mutations were identified in 51.5 and 40% of polycystic and non-polycystic individuals, respectively. Autosomal dominant polycystic kidney disease (ADPKD) patients with truncating *PKD1* variants showed a trend towards a greater slope of the age-estimated glomerular filtration rate (eGFR) regression line than patients with (i) missense variants, (ii) any *PKD2* mutations and (iii) no detected mutations, according to previous findings. The analysis of BBS individuals showed a similar frequency of *BBS4,9,10* and *12* mutations. Of note, all *BBS4-*mutated patients harbored the novel c.332+1G>GTT variant, which was absent in public databases, however, in our internal database, an additional heterozygote carrier was found. All *BBS4-*mutated individuals originated from the same geographical area encompassing the coastal provinces of Naples.

**Discussion:**

In conclusion, these findings indicate the potential for a genetic panel to provide useful information at both clinical and epidemiological levels.

**Graphic abstract:**

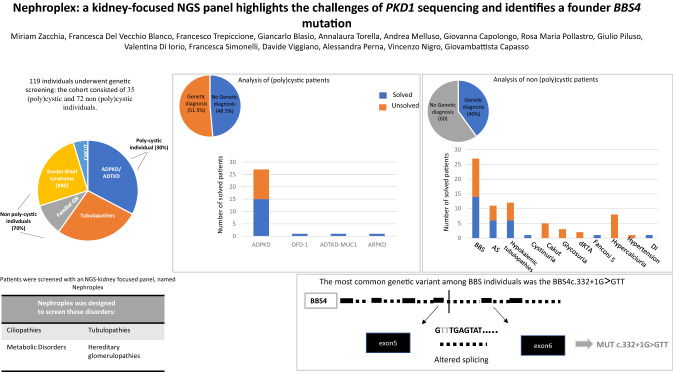

**Supplementary Information:**

The online version contains supplementary material available at 10.1007/s40620-021-01048-4.

## Introduction

The development of “Next-Generation Sequencing” (NGS) has determined a revolution in clinical genetics, thus improving the possibility of increasing sequencing content while dramatically reducing costs, due to the simultaneous analysis of multiple genes through one single reaction [1–3]. Genetic kidney diseases (GKD) are a heterogeneous group of disorders, accounting for approximately 10% of adult chronic kidney disease (CKD) and up to 30% of pediatric CKD patients [4, 5]. It has recently been shown that genetic analysis of GKD patients has a significant clinical impact in terms of either diagnosis and management, reinforcing the rationale for analyzing these patients at the molecular level [4]. While whole exome sequencing (WES) and whole genome sequencing (WGS) are widely used as the first choice genetic analysis, focused genetic panels still retain unique advantages: they produce higher coverage and better separate genes from pseudogenes, an important clue in genetic kidney diseases, especially in the most common one, i.e., autosomal dominant polycystic kidney disease (ADPKD) [6]. In addition, handling large amounts of data produced by WGS and WES requires significant computing power and storage capacity. We developed a gene panel that includes 115 genes causing kidney disorders, including major genetic loci which account for ~ 85–90% of ADPKD and over 96% of Bardet-Biedl syndrome (BBS), namely *PKD1-2* and *BBS1-15*, respectively [7, 8]. Our study illustrates the potential of using Nephroplex to test GKD patients, demonstrating its utility in the molecular diagnosis of classic, challenging and genetically heterogeneous conditions, such as ADPKD and BBS and showing major challenges in *PKD1* analysis.

## Methods

### Patient recruitment and clinical characterization

One-hundred-nineteen subjects referred to the Units of Nephrology of the University of Campania L. Vanvitelli were studied by Nephroplex. This group of individuals included 107 probands and 12 unaffected relatives. Among the 107 probands, 7 were used as positive controls. All probands fulfilled specific diagnostic criteria. Patients were defined as having (poly)cystic kidney diseases (n = 35) and non-(poly)cystic kidney disease (n = 72) (Tables 1 and 2). The former group of individuals included patients with a clinical diagnosis of ADPKD based on the number of kidney cysts and family history [7]; sporadic cases were included when a clear clinical suspicion based on kidney ultrasound or abdominal CT scan was present. Patients with non-cystic disorders were classified as follows: patients with hypokalemic tubulopathies (N = 12), when documented metabolic alkalosis and hypokalemia were ascertained after excluding gastrointestinal and endocrine causes; patients with a clinical suspicion of Alport Syndrome (AS) (n = 11) were defined according to current guidelines [8]. Other tubulopathies were included, such as cystinuria(n = 1) and distal renal tubular acidosis (dRTA) (n = 3). The diagnosis of dRTA was based on a urine acidification test [9], while the Fanconi syndrome patient was defined by the presence of aminoaciduria, low-molecular weight proteinuria and metabolic acidosis due to urine bicarbonate loss that was detected after the loading test. Twenty-seven patients fulfilled the clinical criteria for the diagnosis of Bardet-Biedl syndrome, according to Beales criteria[10]. Further 8 hypercalciuric patients, 1 individual with diabetes insipidus and 5 patients with congenital anomalies of the kidney and urinary tract were included. One patient with familial drug-resistant hypertension. Patients with a likely immune pathogenesis, and other acquired kidney diseases (such as diabetic nephropathy) were excluded. Clinical and laboratory findings, information on familial segregation, and previous genetic testings were requested for each patient (*Zacchia et al, DOI: sfaa182 in Clinical Kidney Journal, in press*). All patients provided written informed consent, in accordance with standard procedures.

**Table 1:** List of cystic individuals, showing major clinical and genetic information

Patient ID	Clinical Diagnosis	Gene	HumanGRCh37/hg19	Sequence	cDNA change	Region	ZYG	Protein variant	exaC	gnomAD	Reference	ACMG Classification	Solved patients
K4	Polycystic kidney disease	PKD1	chr16:2158886	NM_000296	c.6282G>A	EX15	het	p.W2094X	0	0	This study	Pathogenic	x
K5	Polycystic kidney disease	PKD1	chr16:2143910	NM_000296	c.10720_10721insAGGG	EX36	het	p.W3574Rfs*5 (0 53?)	0	0	This study	Pathogenic	x
K6	Polycystic kidney disease	PKD1	chr16:2140418	NM_000296	c.12307_12308delGT	EX45	het	p.V4103Yfs*52	0	0	This study	Pathogenic	x
K9	Polycystic kidney disease	None											
K10	Polycystic kidney disease	None											
K13	Polycystic kidney disease	None											
K14	Polycystic kidney disease	PKD2	chr4:88973292	NM_000297	c.1698_1699insT	EX8	het	p.V569Cfs*4	0	0	[11]	Pathogenic	x
K15	Polycystic kidney disease	PKD2	chr4:88973292	NM_000297	1698_1699insT	EX8	het	p.V569Cfs*4	0	0	[11]	Pathogenic	x
K25	Polycystic kidney disease	PKD1	chr16:2140418	NM_000296	c.12307_12308delGT	EX45	het	V4103fs*52	0	0	This study	Pathogenic	x
K28	Polycystic kidney disease	PKD1	chr16:2160078	NM_000296	c.5090T>G	EX15	het	p.L1697R	0	0	This study	Likely pathogenic	x
		PKD1	chr16:2157957	NM_000296	c.6992C>G	EX16	Het	p.A2331G	0	0.000109	This study	Likely benign	
K31	Polycystic kidney disease	PKD1	chr16:2141795	NM_000296	c.11521T>C	EX41	het	p.W3841R	0	0	[12]	Likely pathogenic	x
		PKD1	chr16:2157957	NM_000296	c.6992C>G	EX16	het	p.A2331G	0	0.000109	This study	Likely benign	
K33	Polycystic kidney disease	PKD1	chr16:2160232	NM_000296	c.4935delC	EX15	het	p.T1646Pfs*76	0	0	[11]	Pathogenic	x
K34	Polycystic kidney disease	PKD1	chr16:2147246	NM_000296	c.10403-4C > T	IVS33	het	Non coding variant	0.001199	0.000467	[13]	Benign	
K44	Polycystic kidney disease	PKD1	chr16:2160991	NM_000296	c.4177C>T	EX15	het	p.Q1393X	0	0	[14]	Pathogenic	x
K54	Polycystic kidney disease	None											
K56	Polycystic kidney disease	PKD1	chr.16:2141032	NM_001009944	c.11856C>G	EX43	het	p.R3952R	− 1	0	This study	likely benign	
		PKD1	chr16:2154478	NM_000296	c.8161+21T>C	IVS22	hom	Non coding variant	0.6267	0.627	[15]	Benign	
		PKD1	chr16:2164211	NM_001009944	c.2813C>T	EX11	het	p.T938M	0.1033	0.0328	This study	Uncertain significance	
		PKD1	chr16:2165395	NM_001009944	c.2081C>T	EX10	het	p.P694L	0.1114	0.0243	This study	Uncertain significance	
		PKD2	chr4:88928968	NM_000297	c.83G>C	EX1	het	p.R28P	0.1538	0.377	[13]	Benign	
		PKD2	chr4:88959381	NM_000297	c.844-22G>A	IVS3	hom	Non coding variant	0.6039	0.604	[16]	Benign	
K57	Polycystic kidney disease	PKD1	chr16:2,158,869	NM_000296	c.6299C>T	EX15	het	p.S2100L	0.0003206	0.0000881	[17]	Uncertain significance	
K60	Polycystic kidney disease	None											
K62	Polycystic kidney disease	PKD1	chr16:2153756	NM_000296	c.8302G>A	EX23	het	p.V2768M	0	0.0000081	[18]	Uncertain significance	
		PKD2	chr4:88967919	NM_000297	c.1445T>G	EX6	het	p.F482C	0.002117	0.00204	This study	Benign	
K66	Polycystic kidney disease	None											
K67	Polycystic kidney disease	PKD1	chr16:2160622	NM_000296	c.4546G>A	EX15	het	p.A1516T	0.006088	0.00576	[13]	Benign	
K70	Polycystic kidney disease	PKD1	chr16:2152434	NM_000296	c.9149_9150insG	EX25	het	p.A3050fs	0	0	This study	Pathogenic	x
		PKD1	chr16:2153345	NM_000296	c.8713G>A	EX23	het	p.V2905I	0.001499	0.0017	[18]	Benign	
K71	Polycystic kidney disease	PKD1	chr16:2154537	NM_000296	c.8123C>T	EX22	het	p.T2708M	0.006665	0.00968	[18]	Uncertain significance	
K72	Polycystic kidney disease	PKD1	chr16:2150039	NM_000296	c.9746T>C	EX29	het	p.L3249P	0	0	This study	Uncertain significance	
		PKD1	chr16:2161666	NM_000296	c.3502C>G	EX15	het	p.P1168A	0	0.0000272	This study	Likely benign	
K76	Polycystic kidney disease	PKD1	chr16:2159259	NM_000296	c.5909C>G	EX15	het	p.A1970G	0	0	This study	Uncertain significance	
K81	Polycystic kidney disease	PKD1	chr16:2159653	NM_000296	c.5515T>A	EX15	het	p.W1839R	0	0	This study	Likely pathogenic	x
K82	Polycystic kidney disease	PKD1	chr16:2143014	NM_000296	c.11094C>G	EX38	het	p.Y3698X	0	0	This study	Pathogenic	x
K84	Polycystic kidney disease	PKD2	chr4:88964539	NM_000297	c.1249C>T	EX5	het	p.R417X	0	0	[11]	Pathogenic	x
K94	Polycystic kidney disease	PKD1	chr16:2160152	NM_000296	c.5014_5015del	EX15	het	p.R1672fs	− 1	0	[18]	Pathogenic	x
		PKD1	chr16:2150245	NM_000296	c.9634T>G	EX28	het	p.F3212V	0.000008621	0.0000121	This study	Uncertain significance	
		PKD1	chr16:2150244	NM_000296	c.9635T>C	EX28	het	p.F3212S	0.000008628	0.0000121	This study	Uncertain significance	
K104	Polycystic kidney disease	PKD1	chr16:2154549	NM_001009944:exon22:	c.8111C>T	EX22	het	p.A2704V	0.003471	0.00142	[13]	Benign	
K125	Polycystic kidney disease	None											
K134	Polycystic kidney disease	PKD1	chr16:2140596	NM_000296	c.12136-5C>T	IVS44	het	spl	0.0006577	0.000545	This study	Likely benign	
K1	Polycystic kidney disease	OFD1	chrX:13757135	NM_003611	c.397_400delAAAG		hem	E134Ifs*10	0	0	[19]	Pathogenic	x
K114	Polycystic kidney disease	PKHD1	chr6: 51712716	NM_138694	c.7964A>G		het	p.H2655R	0	0	This study	Uncertain significance	x
		PKHD1	chr6: 51935203	NM_138694	C.707+1G>A		het	spl			This study	Pathogenic	
K101	multicystic kidney (CAKUT)	MUC1	chr1:155159959	NM_001204295	c.309_316del		het	p.V103fs	− 1	0	This study	Pathogenic	x

**Table 2:** List of non-cystic individuals, with clinical and genetic information

Sample	Gender	Clinical diagnosis	Gene	Genomic position	Genetic sequence	cDNA change	ZYG	Protein change	ExAC	gnomAD	Reference	AMCG classification	Controls	Solved cases
K21	F	Bardet-Biedl syndrome	*BBS12*	chr4:123664149	NM_152618	C1102T	het	p.R368C	0.00	0.00	This study	Uncertain significance	x	
K26	M	Bardet-Biedl syndrome	*BBS9*	chr7:33185869	NM_198428	c.6_6delT	het	p.L3Yfs*38	0.00	0.00	This study	Pathogenic	x	x
			*BBS9*	chr7:33384190	NM_198428	c.1276-2_1277delAGCA	het	Q426Sfs*5	0.00	0.00	This study	Pathogenic		
K29	M	Bardet-Biedl syndrome	*BBS2*	chr16:56531713	NM_031885	c.T1739G	het	p.L580R	0.00	0.00	This study	Uncertain significance	x	
K30	F	Bardet-Biedl syndrome	*BBS4*	chr15:73007744	NM_033028	c.332+1G>GTT	hom	Spl?	0.00	0.00	This study			x
K32	F	Bardet-Biedl syndrome	*BBS4*	chr15:73007744	NM_033028	c.332+1G>GTT	hom	spl?	0.00	0.00	This study			x
K40	F	Bardet-Biedl syndrome	*TTC8*	chr14:89338776	NM_001288782	c.C733T	het	p.R245W	0.00	0.00	This study	Uncertain significance		
K41	M	Bardet-Biedl syndrome	*BBS12*	chr4:123665070	NM_152618	c.C2023T	hom	p.R675X	0.00	0.00	[20]	Pathogenic	x	x
K45	M	Bardet-Biedl syndrome	*BBS10*	chr12:76741493	NM_024685	c.272_273insT	hom	p.C91Lfs*5	0.00	0.00	This study	Pathogenic		x
K46	M	Bardet-Biedl syndrome	*BBS12*	chr4:123665070	NM_152618	c.C2023T	hom	p.R675X	0.00	0.00	[20]	Pathogenic		x
K49	F	Bardet-Biedl syndrome	*BBS4*	chr15:73007744	NM_033028	c.332+1G>GTT	hom	spl?	0.00	0.00	This study			x
K50	M	Bardet-Biedl syndrome	*BBS2*	chr16:56533706	NM_031885	c.C1511T	het	p.A504V	0.01	0.00	[21]	Benign	x	
K58	F	Bardet-Biedl syndrome	*BBS10*	chr16:2159557	NM_024685	c.641T>A	hom	p.V214E	0.00	0.00	This study	Uncertain significance	x	x
K59	F	Bardet-Biedl syndrome	None											
K69	M	Bardet-Biedl syndrome	None											
K73	F	Bardet-Biedl syndrome	*BBS9*	chr7:33296990	NM_	c.585_586del	het	p.V196LFs*10	0.00	0.00	This study	Pathogenic		x
				chr7:33545112	NM_	c.2033delG	het	p.G678Afs*10	0.00	0.00	This study	Pathogenic		
K88	F	CARRIER(K73 mother)	*BBS9*	chr7:33296990	NM_	c.585_586del	het	p.V196LFs*10	0.00	0.00	This study	Pathogenic		
K89	M	carrier(k73 father)	*BBS9*	chr7:33545112	NM_	c.2033delG	het	p.G678Afs*10	0.00	0.00	This study	Pathogenic		
K74	M	Bardet-Biedl syndrome	*BBS4*	chr15:73007744	NM_033028	c.332+1G>GTT	het	spl			This study			x
			*BBS4*	chr15:73027508	NM_033028	c.1091C>A	het	p.A364E	0.00	0.00	[22]	Likely pathogenic		
K77	F	Bardet-Biedl syndrome	*BBS9*	chr7: 33312706	NM_001348042	c.T785C	hom	p.V262A	0.00	0.00	[23]	Likely pathogenic		x
K83	M	Bardet-Biedl syndrome	None											
K87	F	Bardet-Biedl syndrome	*BBS9*	chr7:33380537	NM_001033604	c.T1227A	het	p.D409E	0.00	0.00	This study	Uncertain significance		
			*BBS4*	chr15:73009119	NM_033028	c.334_338del	het	p.112_113del	0.00	0.00	This study	Pathogenic		
K105		Bardet-Biedl syndrome	*BBS12*	chr4:123663945	NM_152618	c.898C>T	hom	p.Q300X	0.00	0.00	This study	Pathogenic		x
K107		Bardet-Biedl syndrome	None											
K115		Bardet-Biedl syndrome	None											
K116		Bardet-Biedl syndrome	*BBS10*	chr12:76741234	NM_024685	c.531C>A	het	p.Y177X	0.00	0.00	This study	Pathogenic		x
			*BBS10*	chr12:76741492	NM_024685	c.273C>G	het	p.C91W	0.00	0.00	[24]	Pathogenic		
K124	M	Bardet-Biedl syndrome	None											
K128		Bardet-Biedl syndrome	None											
K131		Bardet-Biedl Syndrome	*BBS9*	chr7:33195293	NM_001348042	c.175del	het	p.C59fs*20	0.00	0.00	This study	Pathogenic		x
			*BBS9*	chr7:33384190	NM_001348042	C.1141_1142del	het	p.Q381Sfs*5	0.00	0.00	This study	Pathogenic		
K132		Bardet-Biedl syndrome	*bbs4*	chr15:73007744	NM_033028	c.332+1G>GTT	het	Spl?			This study			
K75	M	Alport syndrome	no mut											
K85	M	Alport syndrome	*COL4A5*	chrX:107816858	NM_000495	c.520G>C	hem	p.G174R	0.00	0.00	[25]	Pathogenic		x
K95		Alport Syndrome	None											
K96		Alport Syndrome	None											
K100		Alport Syndrome	*COL4A5*	chrX:107816858	NM_000495	c.520G>C	het	p.G174R	0.00	0.00	[25]	Pathogenic		x
K109		Alport Syndrome	*COL4A3*	chr2:228159682	NM_000091	c.3421C>A	het	p.L1141I	− 1.00	0.00	This study	Uncertain significance		
			*COL4A3*	chr2:228159677	NM_000091	c.3419-3C>A	het	spl	− 1.00	0.00	This study	Uncertain significance		
			*COL4A3*	chr2:228172415	NM_000091	c.4253-11G>C	het	spl	0.00	0.00	This study	Uncertain significance		
K110		Alport Syndrome	*COL4A5*	chrX:107816858	NM_000495	c.520G>C	het	p.G174R	0.00	0.00	[25]	Pathogenic		x
K99		Alport Syndrome	*COL4A5*	chrX:107816858	NM_000495	c.520G>C	het	p.G174R	0.00	0.00	[25]	Pathogenic		x
K121		Alport Syndrome	*COL4A5*	chrX:107868947	NM_000495.5	c.3032deIC	het	p.P1011Lfs*10	0.00	0.00	This study	Pathogenic		x
K122		Alport Syndrome	*COL4A5*	chrX:107868947	NM_000495.5	c.3032deIC	het	p.P1011Lfs*10	0.00	0.00	This study	Pathogenic		x
K42	F	Alport syndrome	*COL4A4*	chr2:227920747	NM_000092	c.G2630A	het	p.R877Q	0.00	0.00	[26]	Benign	x	
			*COL4A4*	chr2:227973562	NM_000092	c.680G>A	het	p.R227H	0.00	0.00	This study	Uncertain significance		
K3	M	Tubulopahty (fanconi)	*SLC2A2*	chr3:170720364	NM_001278658.2	c.711+1G>A	het	p.R182X	0.00	0.00	This study	Pathogenic		x
K16	F	Tubulopathy (hypokalemic metabolic acidosis)	None											
K22		Carrier (K16 mother)												
K17	F	Tubulopathy (hypokalemic metabolic acidosis)	*SLC12A3*	chr16:56938322	NM_000339	c.2899A>G	hom	p.R967G	0.00	0.00	This study	Likely pathogenic		x
K18	F	Tubulopathy (hypokalemic metabolic acidosis)	*SLC12A3*	chr16:56933468	NM_000339	c.2687G>A	het	p.R896Q	0.00	0.00	[27]	Likely pathogenic		x
			*SLC12A3*	chr16:56933491	NM_000339	c.2710A>T	het	p.I904F	0.00	0.00	This study	Uncertain significance		
K19	F	Tubulopathy (hypokalemic metabolic acidosis)	*SLC12A3*	chr16:56933468	NM_000339	G2687A	het	p.R896Q	0.00	0.00	[27]	Likely pathogenic		x
			*SLC12A3*	chr16:56933491	NM_000339	c.2710A>T	het	p.I904F	0.00	0.00	This study	Uncertain significance		
K36	F	Tubulopathy (hypokalemic metabolic alkalosis	None											
K39	F	Tubulopathy (hypokalemic metabolic acidosis)	*SLC12A3*	chr16:56916409	NM_000339	c.1669G>C	het	p.G557R	0.00	0.00	This study	Likely pathogenic		x
			*SLC12A3*	chr16:56921879	NM_000339	c.2221G>A	het	p.G741R	0.00	0.00	[28]	Likely pathogenic		
K47	F	Tubulopathy (hypokalemic metabolic alkalosis	None											
K65	F	Tubulopathy (hypokalemic metabolic alkalosis	None											
K78	M	Tubulopathy (hypokalemic metabolic alkalosis	None											
K103		Tubulopathy/hypokalemic metabolic alkalosis)	*CLCNKB*	chr1:16378205	NM_000085	c.1298G>A	hom	p.G433E	0.00	0.00	[29]	Likely pathogenic		x
K106		Tubulopathy/hypokalemic metabolic alkalosis)	None											
			*SLC12A3*	chr16:56913006	NM_000339	c.1202C>T	het	p.A401V	− 1.00	0.00	This study	Uncertain significance		
K117		Tubulopathy/hypokalemic metabolic alkalosis)	*CLCNKB*	chr1:16374487	NM_000085	c.446T>A	hom	p.V149E	0.00	0.00	[30]	Likely pathogenic		x
K2	F	CAKUT	None											
K35	M	CAKUT	None											
K98		CAKUT	None											
K61	F	CAKUT	None											
K63	M	CAKUT	None											
K52	F	Tubulopahty (Renal Glucosuria)	*SLC5A2*	chr16:31499413	NM_003041	c.940T>C	het	p.C314R	0.00	0.00	This study	Uncertain significance		
			*SLC5A2*	chr16:31500293	NM_003041	c.1373C>A	het	p.A458E	0.00	0.00	This study	Uncertain significance		
K86	F	Tubulopathy (Renal Glucosuria)	None											
K68	F	Tubulopathy (Renal Glucosuria)	*SLC5A2*	chr16:31499413	NM_003041	c.940T>C	het	p.C314R	0.00	0.00	This study	Uncertain significance		
K43	M	Tubulopahty (Hypercalciuria)	None											
K53	F	Tubulopathy (hypercalciuria)	SLC34A1	chr5:176812822	NM_001167579	c.80C>T	het	p.T27M	0.00	0.00	This study	Uncertain significance		
			*EHHADH*	chr3:184910069	NM_001166415	c.1829A>T	het	p.N610I	0.00	0.00	Benign	Benign		
K27	F	Tubulopahty (Hypercalciuria)	None											
K97		Tubulopathy (hypercalciuria)												
K7	F	Tubulopathy (hypercalciuria)	None											
K8	M	Tubulopathy (hypercalciuria)	None											
K120		Tubulopathy (hypercacliuria)	None											
K133	F	Tubulopathy (hypercacliuria)	None											
K11	F	Tubulopathy (RTA)	None											
K102		Tubulopathy (RTA)	*ATP6V0A4*	chr7:138434066	NM_020632	c.1030-6T>-	het	spl	− 1.00		This study	Uncertain significance		
K123		Tubulopathy (cystinuria)	*SLC7A9*	chr19:33355167	NM_001126335	c.313G>A	het	p.G105R	0.00	0.00	[30]	Pathogenic		x
			*SLC7A9*	chr19:33324187	NM_001126335	c.1266_1267del	het	p.L424Gfs*63	0.00	0.00	This study	Pathogenic		
K12	M	Tubulopathy (Diabetes Insipidus)	*AVPR2*	chrX:153171298	NM_000054	c.338G>A	hem	p.R113Q	0.00	0.00	This study	Likely pathogenic		x
K20	M	Tubulopathy (hypertension)	None											

The glomerular filtration rate(GFR) was estimated (eGFR) using the CKD-EPI formula : eGFR = 141 × min(serum Creatinine/κ, 1)^α^ × max(SCr /κ, 1)^−1.209^ × 0.993^Age^ x1.018 [if female] × 1.159 [if Black], according to the literature and using standardized serum creatinine (SCr) [31].

All studies were conducted according to the international guidelines and to the tenets of the 2008 and 2013 Helsinki Declaration. In addition, the study was approved by the Ethics Committee of the University of Campania, L. Vanvitelli.

### Gene panel construction and validation

A custom enrichment tool, named Nephroplex, covering all exons and at least ten flanking nucleotides of the 115 genes causing different inherited kidney diseases was built (Supplemental Table 1). Gene selection was conducted based on literature analysis showing the association between chosen genetic loci and human disease. As a strategy for targeting regions of interest, corresponding to 338.809 Kbp, the HaloPlex TM Target Enrichment System (Agilent) was used.

### DNA extraction and NGS workflow

DNA samples were extracted from whole blood, using standard procedures. DNA quality and quantity were assessed using both spectrophotometric (Nanodrop ND 1000, Thermo Scientific Inc., Rockford, IL, USA) and fluorometry-based (Qubit 2.0 Fluorometer, Life Technologies, Carlsbad, CA, USA) methods, according to the manufacturer’s instructions (HaloPlex Target Enrichment System for Illumina Sequencing, Agilent Technologies, Santa Clara, CA, USA). For library preparation, 200 ng of genomic DNA was digested in restriction reactions for each individual. The fragments were hybridized to specific probes, as described elsewhere [32]. After the capture of target DNA, fragments were closed by a ligase, captured and amplified by PCR. The enriched target DNA in each library sample was validated and quantified by microfluidics analysis using the Bioanalyzer High Sensitivity DNA Assay kit (Agilent Technologies) and the 2100 Bioanalyzer with the 2100 Expert Software. All samples were analyzed in 4 different experimental sections, with a mean of 30 samples per run. Each group was loaded on a single lane of HIseq1000 Illumina system.

### Targeted sequencing analysis

The libraries were sequenced using the HiSeq1000 system (Illumina, San Diego, CA, USA). The generated sequences were analyzed using eXSP, an in-house pipeline designed to automate the analysis workflow, composed of modules performing every step using the appropriate tools available to the scientific community or developed in-house [33]. Paired sequencing reads were aligned to the reference genome (UCSC, hg19 build) using BWA and sorted with SAM tools and Picard (http://picard.sourceforge.net). Post alignment processing (local realignment around insertions-deletions and base recalibration) and SNV and small insertions-deletions (ins-del) calling were performed using the Genome Analysis Toolkit (GATK) [34] with parameters adapted to the haloplex-generated sequences. The called SNV and ins-del variants produced with both platforms were annotated using ANNOVAR [35] with; the relative position in genes using RefSeq [36] gene model, amino acid change, presence in dbSNP v137 [37], frequency in NHLBI Exome Variant Server (http://evs.gs.washington.edu/EVS) and the 1000 genomes large scale projects, multiple cross-species conservation and prediction scores of damaging on protein activity [38]. The annotated variants were then imported into the internal variation database.

### Validation of nephroplex

To design the Nephroplex-panel, a straightforward procedure was followed. Briefly, disease genes causing major inherited kidney disorders were selected. The target sequences were enriched by the HaloPlex system (see “Methods” Section). To validate NephroPlex, the analysis included DNA samples belonging to patients with known genetic mutations (n = 7, see Table 1), with 100% specificity. The average read depth of the target region was more than 98% at 20 × and more than 90% at 100 ×. Damaging variants were validated by Sanger sequencing. Primers for PCR were designed using PRIMER3PLUS free software (http://www.bioinformatics.nl/cgi-bin/primer3plus/primer3plus.cgi) and synthesized by Eurofins Genomics. Sanger sequencing was performed using the BigDye Terminator v1.1 cycle sequencing kit and ABI3130xl, as suggested by the manufacturer (thermoFisher). For validation of *PKD1* variants, Long PCR was performed to discriminate the *PKD1* gene from the pseudogene overlapping region (exon1- exon34), as reported by Tan YC et al [39].

### Variant interpretation

To provide clinically relevant data, a multidisciplinary board consisting of geneticists and nephrologists reviewed the analysis in the context of clinical data. To identify causal variants, the latter were first prioritized based on frequency in public databases (http://www.broadinstitute.org/) and in the internal database, using a minimum allele frequency (MAF)<1% as the cut-off . Then, among the rare variants, we selected exonic and splicing mutations. These variants were searched for in public databases, such as CLINVAR (https://www.ncbi.nlm.nih.gov/clinvar/), HGMD (http://www.hgmd.cf.ac.uk/ac/index.php), and, with regard to ADPKD patients, also in the MAYO CLINIC Database. All variants were classified into the five categories defined by ACMG standards: pathogenic (P), likely pathogenic (LP), uncertain significance (US), likely benign (LB) and benign (B). P variants were defined as such when reported in the literature as deleterious or when they resulted in protein truncation. LP variants were defined in the same way as those previously established as LP in the literature; LB and B variants were those defined as such by other articles or that were predicted not to be damaging by *in silico* programs, such as SIFT and Polyphen.

### Statistics

To compare the effect of genetic mutations (truncating *PKD1* vs non-truncating) on the eGFR decrease as a function of age we used covariance analysis with eGFR as the dependent variable, age as covariate and type of mutation (truncating vs. non truncating) as factor. Statistical significance was accepted for p<0.05.

## Results

### Patient cohort

One-hundred-nineteen subjects were enrolled in the study, including 107 probands and 12 relatives. Probands underwent genetic analysis to address the molecular basis of the following clinical pictures: inherited polycystic diseases (N = 35) (Table 1); among non-cystic patients, the following categories were included in the study; hypokalemic metabolic alkalosis tubulopathies (n = 12), Fanconi syndrome (n = 1), cystinuria (n = 1), renal glycosuria (n = 3), distal renal tubular acidosis /dRTA (n = 2), hypercalciuria (n = 8), diabetes insipidus (n = 1), Alport Syndrome/ AS(n = 11), congenital anomalies of kidney and urinary tract (CAKUT)(N = 5), Bardet-Biedl Syndrome/ BBS (n = 27), and resistant hypertension (n = 1) (Table 2). Most patients were analyzed as single/sporadic cases (97 out of 107 patients), and ten as familial.

### Molecular analysis of polycystic patients

**Fig. 1:**
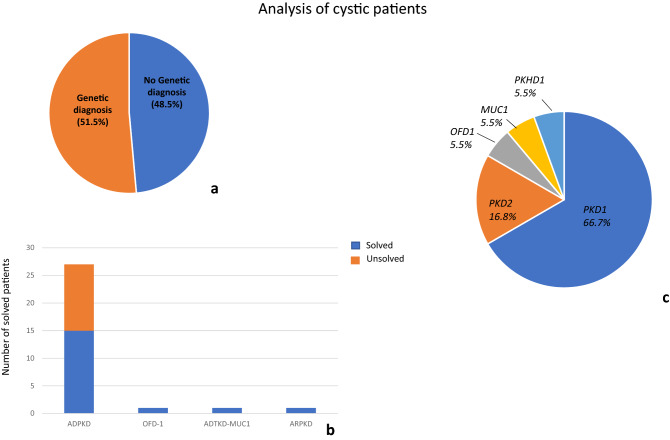
Genetic analysis of cystic patients. 1a Genetic diagnosis was obtained for 51.5% of patients, the remaining showed either variants of uncertain significance (VUS) or no causative variants. 1b. Genetic analysis confirmed the diagnosis of autosomal dominant polycystic kidney dissease (ADPKD) in 15 individuals, Oro-facio-digital type 1 Syndrome (OFD-1, n  =  1), autosomal dominant tubulointerstitial kidney disease (ADTKD, n = 1) and autosomal recessive polycystic kidney disease (ARPKD, n = 1). 1c. Among the pathogenic variants, our results showed that the main mutations occurred in *PKD1*, followed by *PKD2*. *OFD1*, *MUC1* and *PKHD1* mutations were less frequent

Thirty-five individuals underwent genetic analysis due to the clinical suspicion of inherited kidney cystic diseases. A causative mutation was found in over 51% of patients studied; the remaining patients showed either variants of uncertain significance (VUS) or no putative genetic mutations (Fig. 1a). Thirty-six variants in *PKD1* and *PKD2* were found in 25 individuals; twenty-two variants were novel, the remaining were already described in the literature. *PKD1* variants occurred with higher frequency than *PKD2* (83.4% *vs* 16.6%, respectively). We detected damaging *PKD1-2* variants in 15 individuals; 12 in *PKD1* and* 3* in *PKD2,* respectively (Figs. 1b and C). Twelve pathogenic variants were truncating variants, while the remaining were missense variants. Seven out of 12 damaging *PKD1* mutations were located in duplicated regions. Finally, seven patients did not show rare variants in the genes of interest. Interestingly, analysis of covariance of eGFR using age as covariate and type of mutation (truncating *PKD1* variants vs. all others, including no detected variants) as factor revealed significant effects for age (F = 5.87, p = 0.027) and borderline age x mutation type interaction effect (F = 3.1, p = 0.09). This was due to the greater slope of the age-eGFR regression line in the group with *PKD1-* truncating patients, as indicated by previous studies [40, 41]. Indeed, regression analysis in this group showed that each year of age led to a mean loss of eGFR of 2.54 ml/min/1.73m^2^, whereas in the non-truncating group the loss of eGFR was of 0.73ml/min/1.73m^2^ (Supplemental Fig. 1). Moreover, we found a frameshift hemizygote *OFD1* mutation in a young female patient, one patient with compound heterozygote *PKHD1* variants, and one patient with a frameshift *MUC1* variant (Fig. 1b and c).

### Molecular analysis of non (poly)cystic patients

#### BBS individuals

Two of the 27 BBS individuals were studied as trios (K73 and K128). Nine patients showed homozygote variants and five patients had compound heterozygote variants in known BBS genes. Six patients showed only heterozygote BBS variants, while 7 patients did not show any alteration in the genes of interest (Table 2). Major variants were predicted as likely pathogenic or pathogenic (see Table 2). The most common mutations were detected in *BBS10, BBS12, BBS4,* and *BBS9* genes.

#### Alport Syndrome patients

Eleven patients (3 males and 8 females) with a clinical suspicion of AS were analyzed. Six indexes showed pathogenic variants: all mutations were in the *COL4A5* gene. Among these patients, four related individuals showed the known *COL4A5*c.520G>C pathogenic variant, while two sisters showed the novel *COL4A5* c.3032deIC variant, resulting in a frameshift mutation (Table 2).

#### Tubulopathies and CAKUT patients

**Fig. 2:**
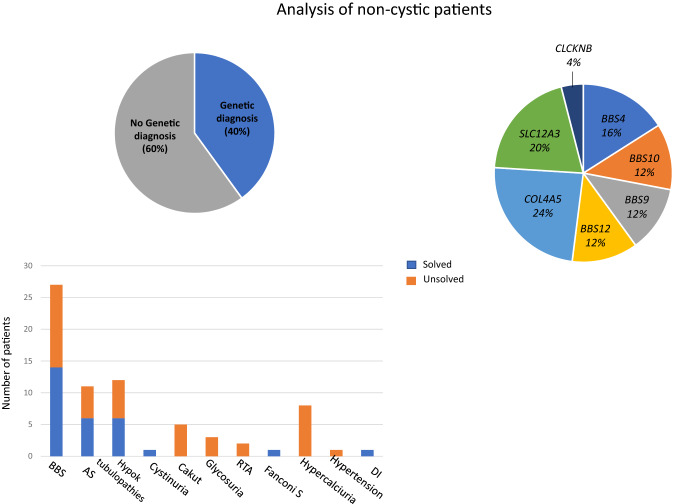
Genetic analysis of non-cystic patients. **a** Forty percent of patients were solved. **b** Classes of disorders and relative number of solved and unsolved individuals. **c** Major pathogenic variants detected in this category of individuals. *BBS* Bardet-Biedl syndrome; *AS* Alport syndrome; *Hypok* hypokalemic; *RTA* renal tubular acidosis; *Fanconi S* Fanconi syndrome; *DI* diabetes insipidus

Patients with hypokalemic metabolic alkalosis of renal origin made up the most substantial subgroup of patients with a clinical suspicion of tubulopathies, accounting for 12 individuals. Six individuals showed either pathogenic or likely pathogenic homozygote or composite heterozygote variants in *SLC12A3* (n = 5) or *CLCKNB* (n = 1). One patient showed only a heterozygote *SLC12A3* variant and the remaining 5 patients showed no mutations in genes of interest. Two out of three patients with renal glycosuria showed variants of uncertain significance in *SLC5A2*; hypercalciuric patients, as well as patients with CAKUT were all unsolved. Major genetic findings of non-cystic individuals are summarized in Fig. 2 and table 2.

#### Frequency of *BBS4*c.332 + 1G > GTT in the patients’ cohort

**Fig. 3:**
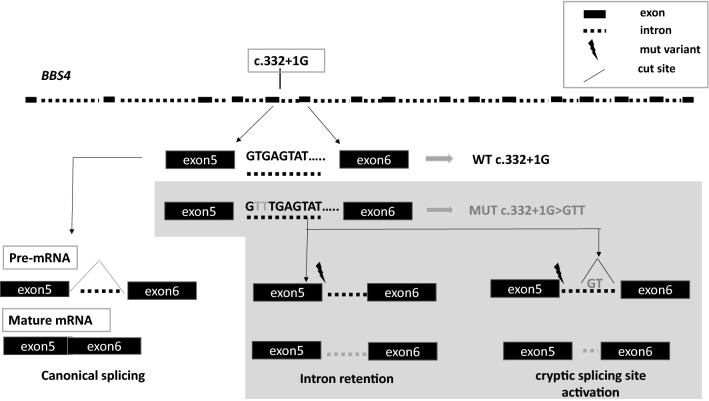
Schematic representation of the BBS4 c. *332* + *1G* > *GTT* variant. The figure shows the possible effects of the genetic variants, according to in silico program: (1) retention of the enthrone; (2) activation of a cryptic site of splicing, with the resulting protein encountering a premature stop codon

We found the c.332+1G>GTT variant in the *BBS4* gene in five unrelated BBS individuals. The variant was homozygote in three BBS patients, while two patients were heterozygote. The predicted effect of genetic mutation of protein function is depicted in Fig. 3. One of the two heterozygote patients showed a second *BBS4* variant, described in the literature as pathogenic. The other patient did not show additional variants in *BBS4,* thus was unsolved. Given the high frequency of this variant, we searched for the variant in our internal database, accounting for 4,000 individuals: besides the cases reported above, it was detected in an additional subject. The latter underwent genetic analysis for the suspicion of AS (K121). The *BBS4* c.332+1G>GTT variant was heterozygote and, was consistent with the autosomal recessive inheritance of BBS. This patient did not show clinical signs of the disorder and was considered an unaffected carrier. Interestingly, individuals harboring the variant showed restricted geographic origin.

#### *PKD1* variants in non-cystic individuals

During the analysis of *PKD1* variants in non-cystic individuals, a high prevalence of *PKD1* variants was observed. We detected a total of 28 rare *PKD1-2* variants in 21 out of 75 adult individuals with non-cystic phenotype (28%). Five variants were detected in *PKD2*, while the remaining variants were found in *PKD1* (18% vs 82%, respectively). Supplemental Table 2 shows the position of the variants and whether they have been previously reported in major public databases, such as Clinvar and/or the Mayo Clinic database. All detected variants were predicted by *in silico* program as benign or likely benign variants, with two exceptions. Patients K7 and K17 underwent genetic analysis due to the clinical suspicion of hypercalciuria and Gitelman syndrome, respectively. K7 was unsolved, while K17 showed a homozygote *SLC12A3* variant, explaining the phenotype. Moreover, our analysis revealed that both individuals carried a *PKD1* variant: a frameshift *PKD1* mutation (K7), predicted as pathogenic, and a missense *PKD1* variant predicted as likely pathogenic (K17), respectively. Both mutations were located in duplicated *PKD1* regions, as well as in 77.3% of detected *PKD1* variants in this subgroup of individuals. To further analyze whether our findings might have been the result of contamination by pseudogenes, we performed ClustalW alignment of *PKD1* with all pseudogene sequences to localize the position of the ‘incidental variants’. Our analysis revealed that all the variants detected in exon 10-33 were located in overlapped regions with almost one pseudogene (see Supplemental Table 2 and additional supplemental material). These findings suggest possible contamination.

## Discussion

In the present study, we set-up and validated a gene panel, named Nephroplex, that includes 115 genes causing inherited kidney disorders, with the aim to define the genetic landscape of a cohort of individuals with kidney cystic and non-cystic phenotype. Recently, WES and WGS have entered into clinical use in several fields. However, there has been a great deal of speculation concerning the perceived advantages and limitations of these studies as compared to focused panels. Costs, time to results, coverage and scalability are major considerations. Given the reduction of costs of NGS, the latter is not a crucial discriminator in choosing sequencing strategies. Moreover, focused gene panels still retain some advantages when used for diagnostic purposes. WGS produces massive amounts of data, requiring intense computational analysis and adequate instrumentation that few clinical laboratories have embraced. The generation of so much sequence data per patient causes low coverage compared with targeted panels, even though this limitation has been overcome in recent studies [42]. Thus, while gene panels and WGS provide similar diagnostic yield, a more laborious analysis is required to handle WGS data. Clearly, WGS offers the advantage of re-analysis paralleling the advances in knowledge and the possibility to discover novel disease, risk and modifier genes, when probands are studied as trios and when data are validated properly.

In our study, the group of polycystic individuals consisted mainly of ADPKD patients. The genetic panel included the two most common genes causing ADPKD, namely *PKD1* and *PKD2* [43, 44]. The study is in line with data from the literature suggesting the superiority of NGS compared with Sanger in analyzing the *PKD1*,  which is a large gene consisting of 46 exons[45, 46]. Molecular screening is unusually difficult, as exons 1–33 have six copies of this region presenting as pseudogenes (PKD1P1-P6), located ~ 13–16Mb proximal to *PKD1*, on the short arm of chromosome 16 [47]. These pseudogenes have early stop codons, so they do not generate large protein products and are 98–99% identical to *PKD1* in homologous regions. This complexity makes molecular diagnosis challenging. Comprehensive screening of well-characterized ADPKD patients has revealed definite (truncating) mutations in up to 61% of affected families, and in-frame changes in ~ 26%, all of which were scored as pathogenic [48, 49]. Screening for larger rearrangements using multiplex ligation-dependent probe amplification detected mutations in a further ~ 4% of families [50]. Non-definite mutations were found in 26% of patients, and ~ 9% of individuals showed no mutations in either *PKD1* or *PKD2* [48]. There are several explanations for this: missed mutations in *PKD1* gene due to technical limitations; *PKD1* pseudogenes; intronic mutations; gene promoter changes; mosaicism; other genes, as recently suggested [51]. In our study, we found a higher prevalence of *PKD1* than *PKD2* mutations in ADPKD patients, just as reported in the literature. Interestingly, in the study we encountered the greatest difficulties during ADPKD molecular diagnosis : (1) the high incidence of private mutations; (2) the large prevalence of missense variants. As largely addressed by experts, the classification of missense variants remains cumbersome given the technical difficulties of performing functional studies. In this scenario, the high allelic heterogeneity of *PKD1* and *PKD2* in non-cystic individuals further complicates molecular diagnosis, as we showed in our cohort. Most variants found in subjects with no clinical ADPKD phenotype were missense variants. Only two patients showed a pathogenic and a likely pathogenic variant, respectively, according to prediction tools. However, the majority of *PKD1* variants in the cohort of non-cystic individuals were located in duplicated regions, including the ones defined as pathogenic: our alignment studies suggest that they may be the result of contamination (*PKD1* gene vs pseudogenes?).

BBS was the second most represented disease in our cohort. The analysis revealed a diagnostic rate of 44%. Interestingly, the study showed a surprisingly high prevalence of *BBS4* variants. *BBS1, 2*, and *10* are known to constitute nearly 50% of diagnoses [52, 53]. One possible explanation is that patients were selected from a cohort consisting of over 60 well-characterized BBS individuals, with most of them possessing a genetic diagnosis at basal. Thus, several *BBS1*-mutated patients were excluded from the study. The high prevalence of *BBS4* mutations in our study was peculiar and attracted our attention. All patients harbored the same *BBS4c.332*+*1G*>*GTT* variant. The latter was homozygote in three unrelated BBS individuals. A fourth patient showed two *BBS4* mutations. An additional BBS subject with no complete molecular diagnosis showed the heterozygote *BBS4c.332*+*1G*>*GTT* variant. The identified mutation is predicted to determine defective splicing. Interestingly, all individuals were from the same region of Southern Italy. Three of 5 *BBS4*-patients were from an area south of Naples, between Torre del Greco and Castellammare di Stabia. The remaining two BBS individuals were from Naples city. A review of both the public and of our own internal database showed no evidence of the variant, except for one additional individual who was identified in the internal database. The patient was a woman born in Naples, undergoing genetic analysis for the clinical suspicion of AS(K121). She showed the heterozygote *BBS4c.332*+*1G*>*GTT* variant, in the absence of any signs of BBS, as expected. Considering the rarity of the disease, with a prevalence of 1:160,000 individuals, the detected *BBS4* variant shows a striking prevalence in Naples, indicating a possible founder mutation. These observations provide the rationale for a cost- and time-efficient screening of this limited geographic area to determine allele frequency distribution and to estimate the risk of BBS occurrence.

Additional non cystic patients in the study included patients suffering from tubulopathies and CAKUT. Fifty percent of patients with hypokalemic metabolic alkalosis were solved as Gitelman Syndrome or Bartter syndrome type 3. Conversely, patients with hypercalciuria and CAKUT were all unsolved. The scarce knowledge of the genetic landscape of these disorders and the contribution of acquired factors to their pathogenesis account at least in part for these results [54, 55].

The present study demonstrates the potential of a kidney focused gene-panel in the diagnosis of renal inherited disorders. In the era of WGS and WES, the potential of focused genetic panels is still of clinical utility and scientific interest, providing advantages when studying inherited kidney disorders in terms of both diagnostic purpose and identification of allele frequency in a restricted geographic area, a pre-requisite to address the risk of occurrence of genetic disorders.

## Supplementary Information

Below is the link to the electronic supplementary material.

Supplementary file1 *PKD1* alignment with PKD1P1- P6. The position of detected variants in non-cystic individuals is marked in yellow. (PDF 216 KB)

Supplementary file2 (DOCX 1389 KB)
