# Supplementary material for: Nephroplex: a kidney-focused NGS panel highlights the challenges of PKD1 sequencing and identifies a founder BBS4 mutation
Source: J Nephrol. 2021 May 8;34(6):1855–74. doi: 10.1007/s40620-021-01048-4 (PMC8610957; doi:10.1007/s40620-021-01048-4)
Supplement: Supplementary file 1 — Supplementary file1 PKD1 alignment with PKD1P1- P6. The position of detected variants in non-cystic individuals is marked in yellow. (PDF 216 KB) [file 40620_2021_1048_MOESM1_ESM.pdf]

CLUSTAL 2.1 multiple sequence alignment

```
PKD1P2          ------------------------------------------------------------
PKD1P5          ------------------------------------------------------------
PKD1            GCACTGCAGCGCCAGCGTCCGAGCGGGCGGCCGAGCTCCCGGAGCGGCCTGGCCCCGAGC
PKD1P4          ------------------------------------------------------------
PKD1P1          ------------------------------------------------------------
PKD1P3          ------------------------------------------------------------
PKD1P6          ------------------------------------------------------------


PKD1P5          --------------GCTCAGCAGCAGGTCGCGGCCGCAGCCCCATCCAGCCCCGCGCCCG
PKD1            CCCGAGCGGGCGTCGCTCAGCAGCAGGTCGCGGCCGCAGCCCCATCCAGCCCCGCGCCCG
PKD1P4          ------------------------------------------------------------
PKD1P1          ------------------------------------------------------------
PKD1P3          --------------GCTCAGCAGCAGGTCGCGGCCGCAGCCCCATCCAGCCCCGCGCCCG
PKD1P6          ------------------------------------------------------------


PKD1P2          ------------------------------------------------------------
PKD1P5          CCATGCCGTCCGCCGGCCCCGCCTGAGCCGCGGCCTCCGCGCGCGGGCGGGCCTGGGGAC
PKD1            CCATGCCGTCCGCGGGCCCCGCCTGAGCTGCGGCCTCCGCGCGCGGGCGGGCCTGGGGAC
PKD1P4          ------------------------------------------------------------
PKD1P1          ------------------------------------------------------------
PKD1P3          CCATGCCGTCCGCCGGCCCCGCCTGAGCCGCGGCCTCCGCGCGCGGGCGGGCCTGGGGAC
PKD1P6          ------------------------------------------------------------


PKD1P2          ------------------------------------------------------------
PKD1P5          GGCGGGGCCATGCGCGCGCTGCCCTAACGATGCCGCCCGCCGCGCCCGCCCGCCTGGCGC
PKD1            GGCGGGGCCATGCGCGCGCTGCCCTAACGATGCCGCCCGCCGCGCCCGCCCGCCTGGCGC
PKD1P4          ------------------------------------------------------------
PKD1P1          -----------------------------ATGCCGCCCGCCGCGCCCGCCCGCCTGGCGC
PKD1P3          GGCGGGGCCATGCGCGCGCTGCCCTAACGATGCCGCCCGCCGCGCCCGCCCGCCTGGCGC
PKD1P6          ------------------------------------------------------------


PKD1P2          ------------------------------------------------------------
PKD1P5          TGGCCCTGGGCCTGGGCCTGTGGCTCGGGGCGCTGGCGGGGGGCCCCGGGCGCGGCTGCG
PKD1            TGGCCCTGGGCCTGGGCCTGTGGCTCGGGGCGCTGGCGGGGGGCCCCGGGCGCGGCTGCG
PKD1P4          ------------------------------------------------------------
PKD1P1          TGGCCCTGGGCCTGGGCCTGTGGCTCGGGGCGCTGGCGGGGGGCCCCGGGCGCGGCTGCG
PKD1P3          TGGCCCTGGGCCTGGGCCTGTGGCTCGGGGCGCTGGCGGGGGGCCCCGGGCGCGGCTGCG
PKD1P6          ------------------------------------------------------------


PKD1P2          ------------------------------------------------------------
PKD1P5          GGCCTTGCGAGCCCCCCTGCCTCTGCGGCCTAGCGCCCGGCGCCGCCTGCCGCGTCAACT
PKD1            GGCCCTGCGAGCCCCCCTGCCTCTGCGGCCCAGCGCCCGGCGCCGCCTGCCGCGTCAACT
PKD1P4          ------------------------------------------------------------
PKD1P1          GGCCTTGCGAGCCCCCCTGCCTCTGCGGCCTAGCGCCCGGCGCCGCCTGCCGCGTCAACT
PKD1P3          GGCCTTGCGAGCCCCCCTGCCTCTGCGGCCTAGCGCCCGGCGCCGCCTGCCGCGTCAACT
PKD1P6          ------------------------------------------------------------


PKD1P2          ------------------------------------------------------------
PKD1P5          GCTCGGGCCGCGGGCCGCGGGCTGCGGACGCTCGGTCCCGCGCTGCGCATCCCCGCGGAC
PKD1            GCTCGGGCCGCGGGC-------TGCGGACGCTCGGTCCCGCGCTGCGCATCCCCGCGGAC
PKD1P4          ------------------------------------------------------------
PKD1P1          GCTCGGGCCGCGGGCCGCGGGCTGCGGACGCTCGGTCCCGCGCTGCGCATCCCCGCGGAC
PKD1P3          GCTCGGGCCGCGGGCCGCGGGCTGCGGACGCTCGGTCCCGCGCTGCGCATCCCCGCGGAC
PKD1P6          ------------------------------------------------------------


PKD1P2          -----------AGACGTCTCCCACAACCTGCTCCGGGCGCTGGACGTTGGGCTCCTGGCG
PKD1P5          GCCACAGCGCTAGACGTCTCCCACAACCTGCTCCGGGCGCTGGACGTTGGGCTCCTGGCG
PKD1            GCCACAGCGCTAGACGTCTCCCACAACCTGCTCCGGGCGCTGGACGTTGGGCTCCTGGCG
PKD1P4          -----------AGACGTCTCCCACAACCTGCTCCGGGCGCTGGACGTTGGGCTCCTGGCG
PKD1P1          GCCACAGCGCTAGACGTCTCCCACAACCTGCTCCGGGCGCTGGACGTTGGGCTCCTGGCG
PKD1P3          GCCACAGCGCTAGACGTCTCCCACAACCTGCTCCGGGCGCTGGACGTTGGGCTCCTGGCG
PKD1P6          ------------------------------------------------------------


PKD1P2          AACCTCTCGGCACTGGCAGAGCTGGATATAAGCAACAACAAGATTTCTACGTTAGAAGAA
PKD1P5          AACCTCTCGGCGCTGGCAGAGCTGGATATAAGCAACAACAAGATTTCTACGTTAGAAGAA
PKD1            AACCTCTCGGCGCTGGCAGAGCTGGATATAAGCAACAACAAGATTTCTACGTTAGAAGAA
PKD1P4          AACCTCTCGGCGCTGGCAGAGCTGGATATAAGCAACAACAAGATTTCTACGTTAGAAGAA
PKD1P1          AACCTCTCGGCGCTGGCAGAGCTGGATATAAGCAACAACAAGATTTCTACGTTAGAAGAA
PKD1P3          AACCTCTCGGCGCTGGCAGAGCTGGATATAAGCAACAACAAGATTTCTACGTTAGAAGAA
PKD1P6          ------------------------------------------------------------


PKD1P2          GGAATATTTGCTAATTTATTTAATTTAAGTGAAATAAACCTGAGTGGGAACCCGTTTGAG
PKD1P5          GGAATATTTGCTAATTTATTTAATTTAAGTGAAATAAACCTGAGTGGGAACCCGTTTGAG
PKD1            GGAATATTTGCTAATTTATTTAATTTAAGTGAAATAAACCTGAGTGGGAACCCGTTTGAG
PKD1P4          GGAATATTTGCTAATTTATTTAATTTAAGTGAAATAAACCTGAGTGGGAACCCGTTTGAG
PKD1P1          GGAATATTTGCTAATTTATTTAATTTAAGTGAAATAAACCTGAGTGGGAACCCGTTTGAG
PKD1P3          GGAATATTTGCTAATTTATTTAATTTAAGTGAAATAAACCTGAGTGGGAACCCGTTTGAG
PKD1P6          ------------------------------------------------------------


PKD1P2          TGTGACTGTGGCCTGGCGTGGCTGCCGCGATGGGCGGAGGAGCAGCAGGTGCGGGTGGTG
PKD1P5          TGTGACTGTGGCCTGGCGTGGCTGCCGCGATGGGCGGAGGAGCAGCAGGTGCGGGTGGTG
PKD1            TGTGACTGTGGCCTGGCGTGGCTGCCGCGATGGGCGGAGGAGCAGCAGGTGCGGGTGGTG
PKD1P4          TGTGACTGTGGCCTGGCGTGGCTGCCGCGATGGGCGGAGGAGCAGCAGGTGCGGGTGGTG
PKD1P1          TGTGACTGTGGCCTGGCGTGGCTGCCGCGATGGGCGGAGGAGCAGCAGGTGCGGGTGGTG
PKD1P3          TGTGACTGTGGCCTGGCGTGGCTGCCGCGATGGGCGGAGGAGCAGCAGGTGCGGGTGGTG
```

```
PKD1P6    ------------------------------------------------------------

PKD1P2    CAGCCCGAGGCAGCCACGTGTGCTGGGCCTGGCTCCCTGGCTGGCCAGCCTCTGCTTGGC
PKD1P5    CAGCCCGAGGCAGCCACGTGTGCTGGGCCTGGCTCCCTGGCTGGCCAGCCTCTGCTTGGC
PKD1      CAGCCCGAGGCAGCCACGTGTGCTGGGCCTGGCTCCCTGGCTGGCCAGCCTCTGCTTGGC
PKD1P4    CAGCCCGAGGCAGCCACGTGTGCTGGGCCTGGCTCCCTGGCTGGCCAGCCTCTGCTTGGC
PKD1P1    CAGCCCGAGGCAGCCACGTGTGCTGGGCCTGGCTCCCTGGCTGGCCAGCCTCTGCTTGGC
PKD1P3    CAGCCCGAGGCAGCCACGTGTGCTGGGCCTGGCTCCCTGGCTGGCCAGCCTCTGCTTGGC
PKD1P6    ------------------------------------------------------------

PKD1P2    ATCCCCTTGCTGGACAGTGGCTGTGGTGAGGAGTATGTCGCCTGCCTCCCTGACAACAGC
PKD1P5    ATCCCCTTGCTGGACAGTGGCTGTGGTGAGGAGTATGTCGCCTGCCTCCCTGACAACAGC
PKD1      ATCCCCTTGCTGGACAGTGGCTGTGGTGAGGAGTATGTCGCCTGCCTCCCTGACAACAGC
PKD1P4    ATCCCCTTGCTGGACAGTGGCTGTGGTGAGGAGTATGTCGCCTGCCTCCCTGACAACAGC
PKD1P1    ATCCCCTTGCTGGACAGTGGCTGTGGTGAGGAGTATGTCGCCTGCCTCCCTGACAACAGC
PKD1P3    ATCCCCTTGCTGGACAGTGGCTGTGGTGAGGAGTATGTCGCCTGCCTCCCTGACAACAGC
PKD1P6    ------------------------------------------------------------

PKD1P2    TCAGGCACCGTGGCAGCAGTGTCCTTTTCAGCTGCCCACGAAGGCCTGCTTCAGCCAGAG
PKD1P5    TCAGGCACCGTGGCAGCAGTGTCCTTTTCAGCTGCCCACGAAGGCCTGCTTCAGCCAGAG
PKD1      TCAGGCACCGTGGCAGCAGTGTCCTTTTCAGCTGCCCACGAAGGCCTGCTTCAGCCAGAG
PKD1P4    TCAGGCACCGTGGCAGCAGTGTCCTTTTCAGCTGCCCACGAAGGCCTGCTTCAGCCAGAG
PKD1P1    TCAGGCACCGTGGCAGCAGTGTCCTTTTCAGCTGCCCACGAAGGCCTGCTTCAGCCAGAG
PKD1P3    TCAGGCACCGTGGCAGCAGTGTCCTTTTCAGCTGCCCACGAAGGCCTGCTTCAGCCAGAG
PKD1P6    ------------------------------------------------------------

PKD1P2    GCCTGCAGCGCCTTCTGCTTCTCCACCGGCCAGGGCCTCGCAGCCCTCTCCGAGCAGGGC
PKD1P5    GCCTGCAGCGCCTTCTGCTTCTCCACCGGCCAGGGCCTCGCAGCCCTCTCCGAGCAGGGC
PKD1      GCCTGCAGCGCCTTCTGCTTCTCCACCGGCCAGGGCCTCGCAGCCCTCTCGGAGCAGGGC
PKD1P4    GCCTGCAGCGCCTTCTGCTTCTCCACCGGCCAGGGCCTCGCAGCCCTCTCCGAGCAGGGC
PKD1P1    GCCTGCAGCGCCTTCTGCTTCTCCACCGGCCAGGGCCTCGCAGCCCTCTCCGAGCAGGGC
PKD1P3    GCCTGCAGCGCCTTCTGCTTCTCCACCGGCCAGGGCCTCGCAGCCCTCTCGGAGCAGGGC
PKD1P6    ------------------------------------------------------------

PKD1P2    TGGTGCCTGTGTGGGTCAGCCCAGCCCTCCAGTGCCTCCTTCGCCTGCCTGTCCCTCTGC
PKD1P5    TGGTGCCTGTGTGGGTCAGCCCAGCCCTCCAGTGCCTCCTTCGCCTGCCTGTCCCTCTGC
PKD1      TGGTGCCTGTGTGGGGCGGCCCAGCCCTCCAGTGCCTCCTTTGCCTGCCTGTCCCTCTGC
PKD1P4    TGGTGCCTGTGTGGGTCAGCCCAGCCCTCCAGTGCCTCCTTCGCCTGCCTGTCCCTCTGC
PKD1P1    TGGTGCCTGTGTGGGTCAGCCCAGCCCTCCAGTGCCTCCTTCGCCTGCCTGTCCCTCTGC
PKD1P3    TGGTGCCTGTGTGGGGCGGCCCAGCCCTCCAGTGCCTCCTTCGCCTGCCTGTCCCTCTGC
PKD1P6    ------------------------------------------------------------

PKD1P2    TCCGGCCCCCCGCCGCCTCCTGCCCCCACCTGTAGGGGCCCCACCCTCCTCCAGCACGTC
PKD1P5    TCCGGCCCCCCGCCGCCTCCTGCCCCCACCTGTAGGGGCCCCACCCTCCTCCAGCACGTC
PKD1      TCCGGCCCCCCGCCACCTCCTGCCCCCACCTGTAGGGGCCCCACCCTCCTCCAGCACGTC
PKD1P4    TCCGGCCCCCCGCCGCCTCCTGCCCCCACCTGTAGGGGCCCCACCCTCCTCCAGCACGTC
PKD1P1    TCCGGCCCCCCGCCGCCTCCTGCCCCCACCTGTAGGGGCCCCACCCTCCTCCAGCACGTC
PKD1P3    TCCGGCCCCCTGCCGCCTCCTGCCCCCACCTGTAGGAGCCCCACCCTCCTCCAGCACGTC
PKD1P6    ------------------------------------------------------------

PKD1P2    TTCCCTGCCTCCCCAGGGGCCACCCTGGTGGGGCCCCACGGACCCCTGGCCTCCGGCCAG
PKD1P5    TTCCCTGCCTCCCCAGGGGCCACCCTGGTGGGGCCCCACGGACCCCTGGCCTCCGGCCAG
PKD1      TTCCCTGCCTCCCCAGGGGCCACCCTGGTGGGGCCCCACGGACCTCTGGCCTCTGGCCAG
PKD1P4    TTCCCTGCCTCCCCAGGGGCCACCCTGGTGGGGCCCCACGGACCCCTGGCCTCCGGCCAG
PKD1P1    TTCCCTGCCTCCCCAGGGGCCCCCCTGGTGGGGCCCCACGGACCCCTGGCCTCCGGCCAG
PKD1P3    TTCCCTGCCTCCCCAGGGGCCGCCCTGGTGGGGCCCCACGGACCCCTGGCCTCCGGCCAG
PKD1P6    ------------------------------------------------------------

PKD1P2    CTAGCAGCCTTCCACATCGCTGCCCCGCTCCCTGTCACTGCCACACGCTGGGACTTCGGA
PKD1P5    CTAGCAGCCTTCCACATCGCTGCCCCGCTCCCTGTCACTGCCACACGCTGGGACTTCGGA
PKD1      CTAGCAGCCTTCCACATCGCTGCCCCGCTCCCTGTCACTGCCACACGCTGGGACTTCGGA
PKD1P4    CTAGCAGCCTTCCACATCGCTGCCCCGCTCCCTGTCACTGCCACACGCTGGGACTTCGGA
PKD1P1    CTAGCAGCCTTCCACATCGCTGCCCCGCTCCCTGTCACTGCCACACGCTGGGACTTCGGA
PKD1P3    CTAGCAGCCTTCCACATCGCTGCCCCGCTCCCTGTCACTGCCACATGCTGGGACTTTGGA
PKD1P6    ------------------------------------------------------------

PKD1P2    GACGGCTCCCCCGAGGTGGATGCCGCTGGGCCGGCTGCCTCGCATCGCTATGTGCTGCCT
PKD1P5    GACGGCTCCCCCGAGGTGGATGCCGCTGGGCCGGCTGCCTCGCATCGCTATGTGCTGCCT
PKD1      GACGGCTCCGCCGAGGTGGATGCCGCTGGGCCGGCTGCCTCGCATCGCTATGTGCTGCCT
PKD1P4    GACGGCTCCCCCGAGGTGGATGCCGCTGGGCCGGCTGCCTCGCATCGCTATGTGCTGCCT
PKD1P1    GACGGCTCCCCCGAGGTGGATGCCGCTGGGCCGGCTGCCTCGCATCGCTATGTGCTGCCT
PKD1P3    GACGGCTCCCCCGAGGTGGATGCCGCTGGGCCGGCTGCCTCGCATCGCTATGTGCTGCCT
PKD1P6    ------------------------------------------------------------

PKD1P2    GGGCGCTATCACGTGACGGCCGTGCTGGCCCTGGGGGCCGGCTCAGCCCTGCTGGGGACA
PKD1P5    GGGCGCTATCACGTGATGGCCGTGCTGGCCCTGGGGGCCGGCTCAGCCCTGCTGGGGACA
PKD1      GGGCGCTATCACGTGACGGCCGTGCTGGCCCTGGGGGCCGGCTCAGCCCTGCTGGGGACA
PKD1P4    GGGCGCTATCACGTGACGGCCGTGCTGGCCCTGGGGACCGGCTCAGCCCTGCTGGGGACA
PKD1P1    GGGCGCTATCACGTGACGGCCGTGCTGGCCCTGGGGACCGGCTCAGCCCTGCTGGGGACA
PKD1P3    GGGCGCTATCACGTGACGGCCGTGCTGGCCCTGGGGACCGGCTCAGCCCTGCTGGGGACA
PKD1P6    ------------------------------------------------------------

PKD1P2    GACGTGCAGGTGGAAGCGGCACCTGCCGCCCTGGAGCTCGTGTGCCCGTCCTCGGTGCAG
PKD1P5    GACGTGCAGGTGGAAGCGGCACCTGCCGCCCTGGAGCTCGTGTGCCCGTCCTCGGTGCAG
PKD1      GACGTGCAGGTGGAAGCGGCACCTGCCGCCCTGGAGCTCGTGTGCCCGTCCTCGGTGCAG
PKD1P4    GACGTGCAGGTGGAAGCGGCACCTGCCGCCCTGGAGCTCGTGTGCCCGTCCTCGGTGCAG
PKD1P1    GACGTGCAGGTGGAAGCGGCACCTGCCGCCCTGGAGCTCGTGTGCCCGTCCTCGGTGCAG
```

```
PKD1P3     GACGTGCAGGTGGAAGCGGCACCTGCCGCCCTGAAGCTCGTGTGCCCGTCCTCGGTGCAG
PKD1P6     ------------------------------------------------------------

PKD1P2     AGTGACGAGAGCCTCGACCTCAGCATCCAGAACCGTGTTGGTTCAGGCCTGGAGGCCGCC
PKD1P5     AGTGACGAGAGCCTCGACCTCAGCATCCAGAACCGCGGTGGTTCAGGCCTGGAGGCCGCC
PKD1       AGTGACGAGAGCCTCGACCTCAGCATCCAGAACCGCGGTGGTTCAGGCCTGGAGGCCGCC
PKD1P4     AGTGACGAGAGCCTCGACCTCAGCATCCAGAACCGCGGTGGTTCAGGCCTGGAGGCCGCC
PKD1P1     AGTGACGAGAGCCTCGACCTCAGCATCCAGAACCGCGGTGGTTCAGGCCTGGAGGCCGCC
PKD1P3     AGTGACGAGAGCCTCGACCTCAGCATCCAGAACCGCGGTGGTTCAGGCCTGGAGGCCGCC
PKD1P6     ------------------------------------------------------------

PKD1P2     TACAGCATCGTGGCCCTGGGCGAGGAGCCGGCCCGAGCGGTGCACCCGCTCTGCCCCTCG
PKD1P5     TACAGCATCGTGGCCCTGGGCGAGGAGCCGGCCCGAGCGGTGCACCCGCTCTGCCCCTCG
PKD1       TACAGCATCGTGGCCCTGGGCGAGGAGCCGGCCCGAGCGGTGCACCCGCTCTGCCCCTCG
PKD1P4     TACAGCATCGTGGCCCTGGGCGAGGAGCCGGCCCGAGCGGTGCACCCGCTCTGCCCCTCG
PKD1P1     TACAGCATCGTGGCCCTGGGCGAGGAGCCGGCCCGAGCGGTGCACCCGCTCTGCCCCTCG
PKD1P3     TACAGCATCGTGGCCCTGGGCGAGGAGCCGGCCCGAGCGGTGCACCCGCTCTGCCCCTCG
PKD1P6     ------------------------------------------------------------

PKD1P2     GACACGGAGATCTTCTCTGGCAACGGGCACTGCTACCGCCTGGTGGTGGAGAAGGCGGCC
PKD1P5     GACACGGAGATCTTCTCTGGCAACGGGCACTGCTACCGCCTGGTGGTGGAGAAGGCGGCC
PKD1       GACACGGAGATCTTCCCTGGCAACGGGCACTGCTACCGCCTGGTGGTGGAGAAGGCGGCC
PKD1P4     GACACGGAGATCTTCTCTGGCAATGGGCACTGCTACCGCCTGGTGGTGGAGAAGGCGGCC
PKD1P1     GACACGGAGATCTTCTCTGGCAATGGGCACTGCTACCGCCTGGTGGTGGAGAAGGCGGCC
PKD1P3     GACACGGAGATCTTCTCTGGCAATGGGCACTGCTACCGCCTGGTGGTGGAGAAGGCGGCC
PKD1P6     ------------------------------------------------------------

PKD1P2     TGGCTGCAGGCGCAGGAGCAGTGTCGGGCCTGGGCCGGGGCCGCCCTGGCAATGGTGGAC
PKD1P5     TGGCTGCAGGCGCAGGAGCAGTGTCGGGCCTGGGCCGGGGCCGCCCTGGCAATGGTGGAC
PKD1       TGGCTGCAGGCGCAGGAGCAGTGTCAGGCCTGGGCCGGGGCCGCCCTGGCAATGGTGGAC
PKD1P4     TGGCTGCAGGCGCAGGAGCAGTGTCGGGCCTGGGCCGGGGCCACCCTGGCAATGGTGGAC
PKD1P1     TGGCTGCAGGCGCAGGAGCAGTGTCGGGCCTGGGCCGGGGCCACCCTGGCAATGGTGGAC
PKD1P3     TGGCTGCAGGCGCAGGAGCAGTGTCGGGCCTGGGCCGGGGCCACCCTGGCAATGGTGGAC
PKD1P6     ------------------------------------------------------------

PKD1P2     AGTCCCGCCGTGCAGCGCTTCCTGGTCTCCCGGGTCACCAGGAGCCTAGACATGTGGATC
PKD1P5     AGTCCCGCCATGCAGCGCTTCCTGGTCTCCCGGGTCACCAGGAGCCTAGACATGTGGATC
PKD1       AGTCCCGCCGTGCAGCGCTTCCTGGTCTCCCGGGTCACCAGGAGCCTAGACGTGTGGATC
PKD1P4     AGTCCCGCCGTGCAGCGCTTCCTGGTCTCCCGGGTCACCAGGAGCCTAGACATGTGGATC
PKD1P1     AGTCCCGCCGTGCAGCGCTTCCTGGTCTCCCGGGTCACCAGGAGCCTAGACATGTGGATC
PKD1P3     AGTCCCGCCGTGCAGCGCTTCCTGGTCTCCCGGGTCACCAGGAGCCTAGACATGTGGATC
PKD1P6     ------------------------------------------------------------

PKD1P2     GGCTTCTCGACTGTGCAGGGGGTGGAGGTGGGCCCAGCGCCGCAGGGCGAGGCCTTCAGC
PKD1P5     GGCTTCTCGACTGTGCAGGGGGTGGAGGTGGGCCCAGCCCCGCAGGGCGAGGCCTTCAGC
PKD1       GGCTTCTCGACTGTGCAGGGGGTGGAGGTGGGCCCAGCGCCGCAGGGCGAGGCCTTCAGC
PKD1P4     GGCTTCTCGACTGTGCAGGGGGTGGAGGTGGGCCCAGCGCCGCAGGGCGAGGCCTTCAGC
PKD1P1     GGCTTCTCGACTGTGCAGGGGGTGGAGGTGGGCCCAGCGCCGCAGGGCGAGGCCTTCAGC
PKD1P3     GGCTTCTCGACTGTGCAGGGGGTGGAGGTGGGCCCAGCGCCGCAGGGCGAGGCCTTCAGC
PKD1P6     ------------------------------------------------------------

PKD1P2     CTGGAGAGCTGCCAGAACTGGCTGCCCGGGGAGCCACACCCAGCCACAGCCGAGCACTGC
PKD1P5     CTGGAGAGCTGCCAGAACTGGCTGCCCGGGGAGCCACACCCAGCCACAGCCGAGCACTGC
PKD1       CTGGAGAGCTGCCAGAACTGGCTGCCCGGGGAGCCACACCCAGCCACAGCCGAGCACTGC
PKD1P4     CTGGAGAGCTGCCAGAACTGGCTGCCCGGGGAGCCACACCCAGCCACAGCCGAGCACTGC
PKD1P1     CTGGAGAGCTGCCAGAACTGGCTGCCCGGGGAGCCACACCCAGCCACAGCCGAGCACTGC
PKD1P3     CTGGAGAGCTGCCAGAACTGGCTGCCCGGGGAGCCACACCCAGCCACAGCTGAGCACTGC
PKD1P6     ------------------------------------------------------------

PKD1P2     GTCCGGCTCGGGCCCACCGGGTGGTGTAACACCGACCTGTGCTCAGCGCTGCACAGCTAC
PKD1P5     GTCCGGCTCGGGCCCACCGGGTGGTGTAACACCGACCTGTGCTCAGCGCCGCACAGCTAC
PKD1       GTCCGGCTCGGGCCCACCGGGTGGTGTAACACCGACCTGTGCTCAGCGCCGCACAGCTAC
PKD1P4     GTCCGGCTCGGGCCCACCGGGTGGTGTAACACCGACCTGTGCTCAGCGCCGCACAGCTAC
PKD1P1     GTCCGGCTCGGGCCCACCGGGTGGTGTAACACCGACCTGTGCTCAGCGCCGCACAGCTAC
PKD1P3     GTCCGGCTCGGGCCCACCGGGTGGTGTAACACCGACCTGTGCTCAGCGCCGCACAGCTAC
PKD1P6     ------------------------------------------------------------

PKD1P2     GTCTGCGAGCTGCGGCCTGGAGGCCCAGTGCAGGATGCCGAGAACCTCCTCGTGGGAGCG
PKD1P5     GTCTGCGAGCTGCGGCCTGGAGGCCCAGTGCAGGATGCCGAGAACCTCCTCGTGGGAGCG
PKD1       GTCTGCGAGCTGCAGCCCGGAGGCCCAGTGCAGGATGCCGAGAACCTCCTCGTGGGAGCG
PKD1P4     GTCTGCGAGCTGCGGCCTGGAGGCCCAGTGCAGGATGCCGAGAACCTCCTCGTGGGAGCG
PKD1P1     GTCTGCGAGCTGCGGCCTGGAGGCCCAGTGCAGGATGCCGAGAACCTCCTCGTGGGAGCG
PKD1P3     GTCTGCGAGCTGCGGCCTGGAGGCCCAGTGCAGGATGCCGAGAACCTCCTCGTGGGAGCG
PKD1P6     ------------------------------------------------------------

PKD1P2     CCCAGTGGGGACCTGCAGGGACCCCTGATGCCTCTGGCACGGCAGTACGGCCTCTCAGCC
PKD1P5     CCCAGTGGGGACCTGCAGGGACCCCTGATGCCTCTGGCACGGCAGGACGGCCTCTCAGCC
PKD1       CCCAGTGGGGACCTGCAGGGACCCCTGACGCCTCTGGCACAGCAGGACGGCCTCTCAGCC
PKD1P4     CCCAGTGGGGACCTGTAGGGACCCCTGATGCCTCTGGCACGGCAGGACGGCCTCTCAGCC
PKD1P1     CCCAGTGGGGACCTGTAGGGACCCCTGATGCCTCTGGCACGGCAGGACGGCCTCTCAGCC
PKD1P3     CCCAGTGGGGACCTGTAGGGACCCCTGATGCCTCTGGCACGGCAGTACGGCCTCTCAGCC
PKD1P6     ------------------------------------------------------------

PKD1P2     CCGCATGAGCCCGTGGAGGTCATGGTATTCCCGGGCCTGCGTCTGAGCCGTGAAGCCTTC
PKD1P5     CCGCATGAGCCCGTGGAGGTCATGGTATTCCCGGGCCTGCGTCTGAGCCGTGAAGCCTTC
PKD1       CCGCACGAGCCCGTGGAGGTCATGGTATTCCCGGGCCTGCGTCTGAGCCGTGAAGCCTTC
PKD1P4     CCGCACGAGCCCGTGGAGGTCATGGTATTCCCGGGCCTGCGTCTGAGCCGTGAAGCCTTC
```

```
PKD1P1          CCGCACGAGCCCGTGGAGGTCATGGTATTCCCGGGCCTGCGTCTGAGCCGTGAAGCCTTC
PKD1P3          CCGCACGAGCCCCTGGAGGTCATGGTATTCCCGGGCCTGCGTCTGAGCCGTGAAGCCTTC
PKD1P6          ------------------------------------------------------------

PKD1P2          CTCACCACGGCCGAATTTGGGACCCAGGAGCTCCGGCGGCCCGCCCAGCTGCGGCTGCAG
PKD1P5          CTCACCACGGCCGAATTTGGGACCCAGGAGCTCCGGCGGCCCGCCCAGCTGCGGCTGCAG
PKD1            CTCACCACGGCCGAATTTGGGACCCAGGAGCTCCGGCGGCCCGCCCAGCTGCGGCTGCAG
PKD1P4          CTCACCACGGCCGAATTTGGGACCCAGGAGCTCCGGCGGCCCGCCCAGCTGCGGCTGCAG
PKD1P1          CTCACCACGGCCGAATTTGGGACCCAGGAGCTCCGGCGGCCCGCCCAGCTGCGGCTGCAG
PKD1P3          CTCACCACGGCCGAATTTGGGACCCAGGAGCTCCGGCGGCCCGCCCAGCTGCGGCTGCAG
PKD1P6          ------------------------------------------------------------

                                                      c.1864
                                                        .
PKD1P2          GTGTACCGGCTCCTCAGCACAGCAGGGACCCCGGAGAACGGCAGCGAGCCTGAGAGCAGG
PKD1P5          GTGTACCGGCTCCTCAGCACAGCAGGGACCCCGGAGAACGGCAGCGAGCCTGAGAGCAGG
PKD1            GTGTACCGGCTCCTCAGCACAGCAGGGACCCCGGAGAACGGCAGCGAGCCTGAGAGCAGG
PKD1P4          GTGTACCGGCTCCTCAGCACAGCAGGGACCCCGGAGAACGGCAGCGAGCCTGAGAGCAGG
PKD1P1          GTGTACCGGCTCCTCAGCACAGCAGGGACCCCGGAGAACGGCAGCGAGCCTGAGAGCAGG
PKD1P3          GTGTACCGGCTCCTCAGCACAGCAGGGACCCCGGAGAACGGCAGCGAGCCTGAGAGCAGG
PKD1P6          ------------------------------------------------------------

PKD1P2          TCCCCGGACAACAGGACCCAGCTGGCCCCCGCGTGCATGCCAGGGGGACGCTGGTGCCCT
PKD1P5          TCCCCGGACAACAGGACCCAGCTGGCCCCCGCGTGCATGCCAGGGGGACGCTGGTGCCCT
PKD1            TCCCCGGACAACAGGACCCAGCTGGCCCCCGCGTGCATGCCAGGGGGACGCTGGTGCCCT
PKD1P4          TCCCCGGACAACAGGACCCAGCTGGTCCCCGCGTGCATGCCAGGGGGACGCTGGTGCCCT
PKD1P1          TCCCCGGACAACAGGACCCAGCTGGTCCCCGCGTGCATGCCAGGGGGACGCTGGTGCCCT
PKD1P3          TCCCCGGACAACAGGACCCAGCTGGTCCCCGCGTGCATGCCAGGGGGACGCTGGTGCCCT
PKD1P6          ------------------------------------------------------------

PKD1P2          GGAGCCAACATCTGCTTGCCGCTGGACGCCTCCTGCCACCCC-AGGCCTGCGCCAATGGC
PKD1P5          GGAGCCAACATCTGCTTGCCGCTGGACGCCTCCTGCCACCCC-AGGCCTGCGCCAATGGC
PKD1            GGAGCCAACATCTGCTTGCCGCTGGACGCCTCCTGCCACCCCCAGGCCTGCGCCAATGGC
PKD1P4          GGAGCCAACATCTGCTTGCCGCTGGACGCCTCCTGCCACCCCCAGGCCTGCGCCAATGGC
PKD1P1          GGAGCCAACATCTGCTTGCCGCTGGACGCCTCCTGCCACCCCCAGGCCTGCGCCAATGGC
PKD1P3          GGAGCCAACATCTGCTTGCCGCTGGACGCCTCCTGCCACCCCCAGGCCTGCGCCAATGGC
PKD1P6          ------------------------------------------------------------

PKD1P2          TGCACGTCAGGGGCCAGGGCTACTCGGGGCCCCCTATGCGCTATGGAGAGAGTTCCTCTT
PKD1P5          TGCACGTCAGGGGCCAGGGCTACTCGGGGCCCCCTATGCGCTATGGAGAGAGTTCCTCTT
PKD1            TGCACGTCAGGG-CCAGGGCTACCCGGGGCCCCCTATGCGCTATGGAGAGAGTTCCTCTT
PKD1P4          TGCACGTCAGGG-CCAGGGCTACTCGGGGCCCCCTATGCGCTATGGAGAGAGTTCCTCTT
PKD1P1          TGCACGTCAGGG-CCAGGGCTACTCGGGTCCCCCTATGCGCTATGGAGAGAGTTCCTCTT
PKD1P3          TGCACGTCAGGG-CCAGGGCTACTCGGGTCCCCCTATGCGCTATGGAGAGAGTTCCTCTT
PKD1P6          ------------------------------------------------------------

PKD1P2          CTCCGTTCCCGCGGGGCCCCCCGCGCAGTACTCGGTCACCCTCCACGGCCAGGATGTCCT
PKD1P5          CTCCGTTCCCGCGGGGCCCCCCGCGCAGTACTCGGTCACCCTCCACGGCCAGGATGTCCT
PKD1            CTCCGTTCCCGCGGGGCCCCCCGCGCAGTACTCGGTCACCCTCCACGGCCAGGATGTCCT
PKD1P4          CTCCGTTCCCGCGGGGCCCCCCGCGCAGTACTCGGTCACCCTCCACAGCCAGGATGTCCT
PKD1P1          CTCCGTTCCCGCGGGGCCCCCCGCGCAGTACTCGGTCACCCTCCACGGCCAGGATGTCCT
PKD1P3          CTCCGTTCCCGCGGGGCCCCCCGCGCAGTACTCGGTCACCCTCCACGGCCAGGATGTCCT
PKD1P6          ------------------------------------------------------------

PKD1P2          CATGCTCCCTGGTGACCTCGTTGGCTTGCAGCACGACGCTGGCCCTGGCGCCCTCCCGCA
PKD1P5          CATGCTCCCTGGTGACCTCGTTGGCTTGCAGCACGACGCTGGCCCTGGCGCCCTCCCGCA
PKD1            CATGCTCCCTGGTGACCTCGTTGGCTTGCAGCACGACGCTGGCCCTGGCGCCCTCCTGCA
PKD1P4          CATGCTCCCTGGTGACCTCGTTGGCTTGCAGCACGACGCTGGCCCTGGCGCCCTCCTGCA
PKD1P1          CATGCTCCCTGGTGACCTCGTTGGCTTGCAGCACGACGCTGGCCCTGGCGCCCTCCCGCA
PKD1P3          CATGCTCCCTGGTGACCTCGTTGGCTTGCAGCACGACGCTGGCCCTGGCGCCCTCCCGCA
PKD1P6          ------------------------------------------------------------

PKD1P2          CTGCTCGCCGGCTCCCGGCCACCCTGGTCCCCAGGCCCCGTACCTCTCCGCCAACGCCTC
PKD1P5          CTGCTCGCCGGCTCCCGGCCACCCTGGTCCCCAGGCCCCGTACCTCTCCGCCAACGCCTC
PKD1            CTGCTCGCCGGCTCCCGGCCACCCTGGTCCCCGGGCCCCGTACCTCTCCGCCAACGCCTC
PKD1P4          CTGCTCGCCGGCTCCCGGCCACCCTGGTCCCCAGGCCCCGTACCTCTCCGCCAACGCCTC
PKD1P1          CTGCTCGCCGGCTCCCGGCCACCCTGGTCCCCAGGCCCCGTACCTCTCCGCCAACGCCTC
PKD1P3          CTGCTCGCCGGCTCCCGGCCACCCTGGTCCCCAGGCCCCGTACCTCTCCGCCAACGCCTC
PKD1P6          ------------------------------------------------------------

PKD1P2          GTCATGGCTGCCCCACTTGCCAGCCCAGCTGGAGGGCACTTGGGCCTGCCCTGCCTGTGC
PKD1P5          GTCATGGCTGCCCCACTTGCCAGCCCAGCTGGAGGGCACTTGGGCCTGCCCTGCCTGTGC
PKD1            GTCATGGCTGCCCCACTTGCCAGCCCAGCTGGAGGGCACTTGGGCCTGCCCTGCCTGTGC
PKD1P4          GTCATGGCTGCCCCACTTGCCAGCCCAGCTGGAGGGCACTTGGGCCTGCCCTGCCTGTGC
PKD1P1          GTCATGGCTGCCCCACTTGCCAGCCCAGCTGGAGGGCACTTGGGCCTGCCCTGCCTGTGC
PKD1P3          GTCATGGCTGCCCCACTTGCCAGCCCAGCTGGAGGGCACTTGGGCCTGCCCTGCCTGTGC
PKD1P6          ------------------------------------------------------------

PKD1P2          CCTGCGGCTGCTTGCAGCCACGGAACAGCTCACCGTGCTGCTGGGCCTGAGGCCCAACCC
PKD1P5          CCTGCGGCTGCTTGCAGCCACGGAACAGCTCACCGTGCTGCTGGGCCTGAGGCCCAACCC
PKD1            CCTGCGGCTGCTTGCAGCCACGGAACAGCTCACCGTGCTGCTGGGCTTGAGGCCCAACCC
PKD1P4          CCTGCGGCTGCTTGCAGCCACGGAACAGCTCACCGTGCTGCTGGGCCTGAGGCCCAACCC
PKD1P1          CCTGCGGCTGCTTGCAGCCACGGAACAGCTCACCGTGCTGCTGGGCCTGAGGCCCAACCC
PKD1P3          CCTGCGGCTGCTTGCAGCCACGGAACAGCTCACCGTGCTGCTGGGCCTGAGGCCCAACCC
PKD1P6          ------------------------------------------------------------

PKD1P2          TGGGCTGCGGCTGCCTGGGCGCTATGAGGTCCGGGCAGAGGTGGGCAATGGCGTGTCCAG
PKD1P5          TGGGCTGCGGCTGCCTGGGCGCTATGAGGTCCGGGCAGAGGTGGGCAATGGCGTGTCCAG
```

```
PKD1        TGGACTGCGGCTGCCTGGGCGCTATGAGGTCCGGGCAGAGGTGGGCAATGGCGTGTCCAG
PKD1P4      TGGGCTGCGGCTGCCTGGGCGCTATGAGGTCCGGGCAGAGGTGGGCAATGGCGTGTCCAG
PKD1P1      TGGGCTGCGGCTGCCTGGGCGCTATGAGGTCCGGGCAGAGGTGGGCAATGGCGTGTCCAG
PKD1P3      TGGGCTGCGGCTGCCTGGGCGCTATGAGGTCCGGGCAGAGGTGGGCAATGGCGTGTCCAG
PKD1P6      ------------------------------------------------------------


PKD1P2      GCACAACCTGTCCTGCAGCTTTGACGTGGTCTCCCCAGTGGCTGGGCTGCGGGTCATCTA
PKD1P5      GCACAACCTGTCCTGCAGCTTTGACGTGGTCTCCCCAGTGGCTGGGCTGCGGGTCATCTA
PKD1        GCACAACCTCTCCTGCAGCTTTGACGTGGTCTCCCCAGTGGCTGGGCTGCGGGTCATCTA
PKD1P4      GCACAACCTGTCCTGCAGCTTTGACGTGGTCTCCCCAGTGGCTGGGCTGCGGGTCATCTA
PKD1P1      GCACAACCTGTCCTGCAGCTTTGACGTGGTCTCCCCAGTGGCTGGGCTGCGGGTCATCTA
PKD1P3      GCACAACCTGTCCTGCAGCTTTGACGTGGTCTCCCCAGTGGCTGGGCTGCGGGTCATCTA
PKD1P6      ------------------------------------------------------------

              c.2489
                 .
PKD1P2      CCCTGCCCCCCGCGACGGCCGCCTCTACGTGCCCACCAACGGCTCAGCCTCGGTGCTCCA
PKD1P5      CCCTGCCCCCCGCGACGGCCGCCTCTACGTGCCCACCAACGGCTCAGCCTCGGTGCTCCA
PKD1        CCCTGCCCCCCGCGACGGCCGCCTCTACGTGCCCACCAACGGCTCAGCCTTGGTGCTCCA
PKD1P4      CCCTGCCCCCCGCGACGGCCGCCTCTACGTGCCCACCAACGGCTCAGCCTCGGTGCTCCA
PKD1P1      CCCTGCCCCCCGCGACGGCCGCCTCTACGTGCCCACCAACGGCTCAGCCTCGGTGCTCCA
PKD1P3      CCCTGCCCCCCGCGACGGCCGCCTCTACGTGCCCACCAACGGCTCAGCCTCGGTGCTCCA
PKD1P6      ------------------------------------------------------------


PKD1P2      GGTGGACTCTGGTGCCAGCGCCACGGCCACGGCTCGCTGGCCTGGGGGCAGTGTCAGCGC
PKD1P5      GGTGGACTCTGGTGCCAGCGCCACGGCCACGGCTCGCTGGCCTGGGGGCAGTGTCAGCGC
PKD1        GGTGGACTCTGGTGCCAACGCCACGGCCACGGCTCGCTGGCCTGGGGGCAGTGTCAGCGC
PKD1P4      GGTGGACTCTGGTGCCAGCGCCACGGCCACGGCTCGCTGGCCTGGGGGCAGTGTCAGCGC
PKD1P1      GGTGGACTCTGGTGCCAGCGCCACGGCCACGGCTCGCTGGCCTGGGGGCAGTGTCAGCGC
PKD1P3      GGTGGACTCTGGTGCCAGCGCCACGGCCACGGCTTGCTGGCCTGGGGGCAGTGTCAGCGC
PKD1P6      ------------------------------------------------------------


PKD1P2      CCGCTTTGAGAATGCCTGCCCTGCCCTGGTGGCCACCTTCGTGCCCGGCTGCCCCTGGGA
PKD1P5      TCGCTTTGAGAATGCCTGCCCTGCCCTGGTGGCCACCTTCGTGCCCGGCTGCCCCTGGGA
PKD1        CCGCTTTGAGAATGTCTGCCCTGCCCTGGTGGCCACCTTCGTGCCCGGCTGCCCCTGGGA
PKD1P4      CCGCTTTGAGAATGCCTGCCCTGCCCTGGTGGCCACCTTCGTGCCCGGCTGCCCCTGGGA
PKD1P1      CCGCTTTGAGAATGCCTGCCCTGCCCTGGTGGCCACCTTCGTGCCCAGCTGCCCCTGGGA
PKD1P3      CCGCTTTGAGAATGCCTGCCCTGCCCTGGTGGCCACCTTCGTGCCCGGCTGCCCCTGGGA
PKD1P6      ------------------------------------------------------------
```

```
                                                                 c.2722
                                                                      .
PKD1P2      GACCAATGATACCCTGTTCTCAGTGGTAGCACTGCCGTGGCTCGGTGAGGGGGAGCACGT
PKD1P5      GACCAATGATACCCTGTTCTCAGTGGTAGCACTGCCGTGGCTCGGTGAGGGGGAGCACGT
PKD1        GACCAACGATACCCTGTTCTCAGTGGTAGCACTGCCGTGGCTCAGTGAGGGGGAGCACGT
PKD1P4      GACCAATGATACCCTGTTCTCAGTGGTAGCACTGCCGTGGCTCGGTGAGGGGGAGCACGT
PKD1P1      GACCAATGATACCCTGTTCTCAGTGGTAGCACTGCCGTGGCTCGGTGAGGGGGAGCACGT
PKD1P3      GACCAATGATACCCTGTTCTCAGTGGTAGCACTGCCGTGGCTCGGTGAGGGGGAGCACGT
PKD1P6      ------------------------------------------------------------


PKD1P2      GATGGACGTTGTGGTGGAAAACAGCGCCAGCCGGGCCAACCTCAGCCTGCGGGTGACGGC
PKD1P5      GATGGACGTTGTGGTGGAAAACAGCGCCAGCCGGGCCAACCTCAGCCTGCGGGTGACGGC
PKD1        GGTGGACGTGGTGGTGGAAAACAGCGCCAGCCGGGCCAACCTCAGCCTGCGGGTGACGGC
PKD1P4      GATGGACGTTGTGGTGGAAAACAGCGCCAGCCGGGCCAACCTCAGCCTGCGGGTGACGGC
PKD1P1      GATGGACGTTGTGGTGGAAAACAGCGCCAGCCGGGCCAACCTCAGCCTGCGGGTGACGGC
PKD1P3      GATGGACGTTGTGGTGGAAAACAGCGCCAGCCGGGCCAACCTCAGCCTGCGGGTGACGGC
PKD1P6      ------------------------------------------------------------


PKD1P2      GGAGGAGCCCATCTGTGGCCTCCGCGCCACGCCCAGCCCCGAGGCCCGTGTACTGCAGGG
PKD1P5      GGAGGAGCCCATCTGTGGCCTCCGCGCCACGCCCAGCCCCGAGGCCCGTGTACTGCAGGG
PKD1        GGAGGAGCCCATCTGTGGCCTCCGCGCCACGCCCAGCCCCGAGGCCCGTGTACTGCAGGG
PKD1P4      GGAGGAGCCCATCTGTGGCCTCCGCGCCACGCCCAGCCCCGAGGCCCGTGTACTGCAGGG
PKD1P1      GGAGGAGCCCATCTGTGGCCTCCGCGCCACGCCCAGCCCCGAGGCCCGTGTACTGCAGGG
PKD1P3      GGAGGAGCCCATCTGTGGCCTCCGCGCCACGCCCAGCCCCGAGGCCCGTGTACTGCAGGG
PKD1P6      ------------------------------------------------------------


PKD1P2      AGTCCCAGTGAGGTACAGCCCCGTGGTGGAGGCCGGCTCGGACATGGTCTTCCGGTGGAC
PKD1P5      AGTCCCAGTGAGGTACAGCCCCGTGGTGGAGGCCGGCTCGGACATGGTCTTCCGGTGGAC
PKD1        AGTCCTAGTGAGGTACAGCCCCGTGGTGGAGGCCGGCTCGGACATGGTCTTCCGGTGGAC
PKD1P4      AGTCCCAGTGAGGTACAGCCCCGTGGTGGAGGCCGGCTCGGACATGGTCTTCCGGTGGAC
PKD1P1      AGTCCCAGTGAGGTACAGCCCCGTGGTGGAGGCCGGCTCGGACATGGTCTTCCGGTGGAC
PKD1P3      AGTCCCAGTGAGGTACAGCCCCGTGGTGGAGGCCGGCTCGGACATGGTCTTCTGGTGGAC
PKD1P6      ------------------------------------------------------------


PKD1P2      CATCAACGACAAGCAGTCCCTGACCTTCCAGAACGTGGTCTTCAATGTCATTTATCAGAG
PKD1P5      CATCAACGACAAGCAGTCCCTGACCTTCCAGAACGTGGTCTTCAATGTCATTTATCAGAG
PKD1        CATCAACGACAAGCAGTCCCTGACCTTCCAGAACGTGGTCTTCAATGTCATTTATCAGAG
PKD1P4      CATCAACGACAAGCAGTCCCTGACCTTCCAGAACGTGGTCTTCAATGTCATTTATCAGAG
PKD1P1      CATCAACGACAAGCAGTCCCTGACCTTCCAGAACGTGGTCTTCAATGTCATTTATCAGAG
PKD1P3      CATCAACGACAAGCAGTCCCTGACCTTCCAGAACGTGGTCTTCAATGTCATTTATCAGAG
PKD1P6      ------------------------------------------------------------


PKD1P2      CGCGGCGGTCTTCAAGCTCTCACTGACGGCCTCCAACCACGTGAGCAACGTCACCGTGAA
PKD1P5      CGCGGCGGTCTTCAAGCTCTCACTGACGGCCTCCAACCACGTGAGCAACGTCACCGTGAA
```

```
PKD1       CGCGGCGGTCTTCAAGCTCTCACTGACGGCCTCCAACCACGTGAGCAACGTCACCGTGAA
PKD1P4     CGCGGCGGTCTTCAAGCTCTCACTGACGGCCTCCAACCACGTGAGCAACGTCACCGTGAA
PKD1P1     CGCGGCGGTCTTCAAGCTCTCACTGACGGCCTCCAACCACGTGAGCAACGTCACCGTGAA
PKD1P3     CGCGGCGGTCTTCAAGCTCTCACTGACGGCCTCCAACCACGTGAGCAACGTCACCGTGAA
PKD1P6     ------------------------------------------------------------

PKD1P2     CTACAACATCACCGTGGAGCGGATGAACAGGATGCAGGGCCTGCGGGTCTCCACAGTGCC
PKD1P5     CTACAACATCACCGTGGAGCGGATGAACAGGATGCAGGGCCTGCGGGTCTCCACAGTGCC
PKD1       CTACAACGTAACCGTGGAGCGGATGAACAGGATGCAGGGTCTGCAGGTCTCCACAGTGCC
PKD1P4     CTACAACATCACCGTGGAGCGGATGAACAGGATGCAGGGCCTGCGGGTCTCTACAGTGCC
PKD1P1     CTACAACATCACCGTGGAGCGGATGAACAGGATGCAGGGCCTGCGGGTCTCTACAGTGCC
PKD1P3     CTACAACATCACCGTGGAGCGGATGAACAGGATGCAGGGCCTGCGGGTCTCTACAGTGCC
PKD1P6     ------------------------------------------------------------

PKD1P2     GGCCGTGCTGTCCCCCAATGCCACGCTGGCACTGACGGCGGGCGTGCTGGTGGACTCGGC
PKD1P5     GGCCGTGCTGTCCCCCAATGCCACGCTGGCACTGACGGCGGGCGTGCTGGTGGACTCGGC
PKD1       GGCCGTGCTGTCCCCCAATGCCACGCTAGCACTGACGGCGGGCGTGCTGGTGGACTCGGC
PKD1P4     AGCCGTGCTGTCCCCCAATGCCACGCTGGCACTGACGGCGGGCGTGCTGGTGGACTCGGC
PKD1P1     AGCCGTGCTGTCCCCCAATGCCACGCTGGCACTGACGGCGGGCGTGCTGGTGGACTCGGC
PKD1P3     AGCCGTGCTGTCCCCCAATGCCACGCTGGCACTGACGGCGGGCGTGCTGGTGGACTCGGC
PKD1P6     ------------------------------------------------------------

PKD1P2     CGTGGAGGTGGCCTTCCTGTGGACCTTTGGGGATGGGGAGCAGGCCCTCCACCAGTTCCA
PKD1P5     TGTGGAGGTGGCCTTCCTGTGGACCTTTGGGGATGGGGAGCAGGCCCTCCACCAGTTCCA
PKD1       CGTGGAGGTGGCCTTCCTGTGGACCTTTGGGGATGGGGAGCAGGCCCTCCACCAGTTCCA
PKD1P4     CGTGGAGGTGGCCTTCCTGTGGACCTTTGGGGATGGGGAGCAGGCCCTCCACCAGTTCCA
PKD1P1     CGTGGAGGTGGCCTTCCTGTGGACCTTTGGGGATGGGGAGCAGGCCCTCCACCAGTTCCA
PKD1P3     CGTGGAGGTGGCCTTCCTGTGGACCTTTGGGGATGGGGAGCAGGCCCTCCACCAGTTCCA
PKD1P6     ------------------------------------------------------------

PKD1P2     GCCTCCGTACAACGAGTCCTTCCCGGTTCCAGACCCCTCGGTGGCCCAGGTGCTGGTGGA
PKD1P5     GCCTCCGTACAACGAGTCCTTCCCAGTTCCAGACCCCTCGGTGGCCCAGGTGCTGGTGGA
PKD1       GCCTCCGTACAACGAGTCCTTCCCGGTTCCAGACCCCTCGGTGGCCCAGGTGCTGGTGGA
PKD1P4     GCCTCCGTACAACGAGTCCTTCCCGGTTCCAGACCCCTCGGTGGCCCAGGTGCTGGTGGA
PKD1P1     GCCTCCGTACAACGAGTCCTTCCCGGTTCCAGACCCCTCGGTGGCCCAGGTGCTGGTGGA
PKD1P3     GCCTCCATACAACGAGTCCTTCCCGGTTCCAGACCCCTCGGTGGCCCAGGTGCTGGTGGA
PKD1P6     ------------------------------------------------------------

PKD1P2     GCACAATGTCACGCACACCTACGCTGCCCCAGGTGAGTACGTCCTGACCGTGCTGGCATC
PKD1P5     GCACAATGTCACGCACACCTACGCTGCCCCAGGTGAGTACGTCCTGACCGTGCTGGCATC
PKD1       GCACAATGTCATGCACACCTACGCTGCCCCAGGTGAGTACCTCCTGACCGTGCTGGCATC
PKD1P4     GCACAATGTCACCCACACCTACGCTGCCCCAGGTGAGTACGTCCTGACCTTGCTGGCATC
PKD1P1     GCACAATGTCACCCACACCTACGCTGCCCCAGGTGAGTACGTCCTGACCTTGCTGGCATC
PKD1P3     GCACAATGTCACCCACACCTACGCTGCCCCAGGTGAGTACGTCCTGACCTTGCTGGCATC
PKD1P6     ------------------------------------------------------------

PKD1P2     TAATGCCTTCGAGAACCGGACGCAGCAGGTGCCTGTGAGCGTGCGCGCCTCCCTGCCCTC
PKD1P5     TAATGCCTTCGAGAACCGGATGCAGCAGGTGCCTGTGAGCGTGCGCGCCTCCCTGCCCTC
PKD1       TAATGCCTTCGAGAACCTGACGCAGCAGGTGCCTGTGAGCGTGCGCGCCTCCCTGCCCTC
PKD1P4     TAATGCCTTCGAGAACCGGACGCAGCAGGTGCCTGTGAGCGTGTGCGCCTCCCTGCCCTC
PKD1P1     TAATGCCTTCGAGAACCGGACGCAGCAGGTGCCTGTGAGCGTGTGCGCCTCCCTGCCCTC
PKD1P3     TAATGCCTTCGAGAACCGGACGCAGCAGGTGCCTGTGAGCGTGTGCGCCTCCCTGCCCTC
PKD1P6     ------------------------------------------------------------

                                                      c.3430
                                                        .
PKD1P2     CGAGGCTGTGGGTGTGAGTGACGGCGTCCTGGTGGCCGGCCGGCCCGTCACCTTCTACCC
PKD1P5     CGAGGCTGTGGGTGTGAGTGACGGCGTCCTGGTGGCCGGCCGGCCCGTCACCTTCTACCC
PKD1       CGTGGCTGTGGGTGTGAGTGACGGCGTCCTGGTGGCCGGCCGGCCCGTCACCTTCTACCC
PKD1P4     ------------------------------------------------------------
PKD1P1     ------------------------------------------------------------
PKD1P3     T-----------------------------------------------------------
PKD1P6     ------------------------------------------------------------

PKD1P2     GCATCTGCTGCCCTCGCCTGGGGGTGTTCTTTACACGTGGGACTTCGGGGACGGCTCCCC
PKD1P5     GCATCTGCTGCCCTCGCCTGGGGGTGTTCTTTACACGTGGGACTTCGGGGACGGCTCCCC
PKD1       GCACCCGCTGCCCTCGCCTGGGGGTGTTCTTTACACGTGGGACTTCGGGGACGGCTCCCC
PKD1P4     ------------------------------------------------------------
PKD1P1     ------------------------------------------------------------
PKD1P3     ------------------------------------------------------------
PKD1P6     ------------------------------------------------------------

PKD1P2     TGTCCTGACCCAGAGCCAGCCGGCTGCCAACCACACCTATCCCTCGAGGGGCATCTACCA
PKD1P5     TGTCCTGACCCAGAGCCAGCCGGCTGCCAACCACACCTATCCCTCGAGGGGCATCTACCA
PKD1       TGTCCTGACCCAGAGCCAGCCGGCTGCCAACCACACCTATGCCTCGAGGGGCACCTACCA
PKD1P4     ------------------------------------------------------------
PKD1P1     ------------------------------------------------------------
PKD1P3     ------------------------------------------------------------
PKD1P6     ------------------------------------------------------------

                                                   c.3605
                                                     .
PKD1P2     CGTGCGCCTGGAGGTCAACAACACGGTGAGCGGTGCGGCGGCCCAGGCGGATGTGCGCGT
PKD1P5     CGTGCGCCTGGAGGTCAACAACACGGTGAGCGGTGCGGCGGCCCAGGCGGATGTGCGCGT
PKD1       CGTGCGCCTGGAGGTCAACAACACGGTGAGCGGTGCGGCGGCCCAGGCGGATGTGCGCGT
PKD1P4     ------------------------------------------------------------
PKD1P1     ------------------------------------------------------------
PKD1P3     ------------------------------------------------------------
PKD1P6     ------------------------------------------------------------
```

```
PKD1P2          CTTTGAGGAGCTCCGCGGGCTCAGCGTGGACATGAGCCTGGCCGTGGAGCAGGGCGCCCC
PKD1P5          CTTTGAGGAGCTCCGCGGGCTCAGCGTGGACATGAGCCTGGCCGTGGAGCAGGGCGCCCC
PKD1            CTTTGAGGAGCTCCGCGGACTCAGCGTGGACATGAGCCTGGCCGTGGAGCAGGGCGCCCC
PKD1P4          ------------------------------------------------------------
PKD1P1          ------------------------------------------------------------
PKD1P3          ------------------------------------------------------------
PKD1P6          ------------------------------------------------------------

PKD1P2          CGTGGTGGTCAGTGCCGCGGTGCAGACGGGCGACAACATCACGTGGACCTTCGACATGGG
PKD1P5          CGTGGTGGTCAGTGCCGCGGTGCAGACGGGCGACAACATCACGTGGACCTTCGACATGGG
PKD1            CGTGGTGGTCAGCGCCGCGGTGCAGACGGGCGACAACATCACGTGGACCTTCGACATGGG
PKD1P4          ------------------------------------------------------------
PKD1P1          ------------------------------------------------------------
PKD1P3          ------------------------------------------------------------
PKD1P6          ------------------------------------------------------------

PKD1P2          GGACGGCACCGTGCTGTCGGGCCCAGAGGCCACAGTGGAGCATGTGTACCTGCGGGCACA
PKD1P5          GGACGGCACCGTGCTGTCGGGCCCAGAGGCCACAGTGGAGCATGTGTACCTGCGGGCACA
PKD1            GGACGGCACCGTGCTGTCGGGCCCGGAGGCAACAGTGGAGCATGTGTACCTGCGGGCACA
PKD1P4          ------------------------------------------------------------
PKD1P1          ------------------------------------------------------------
PKD1P3          ------------------------------------------------------------
PKD1P6          ------------------------------------------------------------

PKD1P2          GAACTGCACAGTGACCGTGGGTGCGGCCAGCCCCGCCGGCCACCTGGCCCGGAGCCTGCA
PKD1P5          GAACTGCACAGTGACCGTGGGTGCGGCCAGCCCCGCCGGCCACCTGGCCCGGAGCCTGCA
PKD1            GAACTGCACAGTGACCGTGGGTGCGGCCAGCCCCGCCGGCCACCTGGCCCGGAGCCTGCA
PKD1P4          ------------------------------------------------------------
PKD1P1          ------------------------------------------------------------
PKD1P3          ------------------------------------------------------------
PKD1P6          ------------------------------------------------------------

PKD1P2          CGTGCTGGTCTTCGTCCTGGAGGTGCTGCGCGTCGAGCCCGCCGCCTGCATCCCCACTCA
PKD1P5          CGTGCTGGTCTTCGTCCTGGAGGTGCTGCGCGTCGAGCCCGCCGCCTGCATCCCCACTCA
PKD1            CGTGCTGGTCTTCGTCCTGGAGGTGCTGCGCGTTGAACCCGCCGCCTGCATCCCCACGCA
PKD1P4          ------------------------------------------------------------
PKD1P1          ------------------------------------------------------------
PKD1P3          ------------------------------------------------------------
PKD1P6          ------------------------------------------------------------

PKD1P2          GCCTGACGCGCGGCTCACGGCCTACGTCACCGGGAACCCGGCCCGCTACCTCTTCGACTG
PKD1P5          GCCTGACGCGCGGCTCACGGCCTACGTCACCGGGAACCCGGCCCGCTACCTCTTCGACTG
PKD1            GCCTGACGCGCGGCTCACGGCCTACGTCACCGGGAACCCGGCCCACTACCTCTTCGACTG
PKD1P4          ------------------------------------------------------------
PKD1P1          ------------------------------------------------------------
PKD1P3          ------------------------------------------------------------
PKD1P6          ------------------------------------------------------------

PKD1P2          GACCTTTGGGGATGGCTCCTCCAACACGACCATGCGGGGGTGCCCGACGGTGACACACAA
PKD1P5          GACCTTTGGGGATGGCTCCTCCAACACGACCATGCGGGGGTGCCCGACGGTGACACACAA
PKD1            GACCTTCGGGGATGGCTCCTCCAACACGACCGTGCGGGGGTGCCCGACGGTGACACACAA
PKD1P4          ------------------------------------------------------------
PKD1P1          ------------------------------------------------------------
PKD1P3          ------------------------------------------------------------
PKD1P6          ------------------------------------------------------------

PKD1P2          CTTCACGCGTAGCGGCACGTTCCCCCTGGCGCTGGTGCTGTCCAGCCGCGTGAACAGGGC
PKD1P5          CTTCACGCGTAGCGGCACGTTCCCCCTGGCGCTGGTGCTGTCCAGCCGCGTGAACAGGGC
PKD1            CTTCACGCGGAGCGGCACGTTCCCCCTGGCGCTGGTGCTGTCCAGCCGCGTGAACAGGGC
PKD1P4          ------------------------------------------------------------
PKD1P1          ------------------------------------------------------------
PKD1P3          ------------------------------------------------------------
PKD1P6          ------------------------------------------------------------

PKD1P2          GCGTTACTTCACCAGCATCTGCGTGGAGCCAGAGGTGGGCAACGTCACCCTGCAGCCAGA
PKD1P5          GCGTTACTTCACCAGCATCTGCGTGGAGCCAGAGGTGGGCAACGTCACCCTGCAGCCAGA
PKD1            GCATTACTTCACCAGCATCTGCGTGGAGCCAGAGGTGGGCAACGTCACCCTGCAGCCAGA
PKD1P4          ------------------------------------------------------------
PKD1P1          ------------------------------------------------------------
PKD1P3          ------------------------------------------------------------
PKD1P6          ------------------------------------------------------------

PKD1P2          GAGGCAGTTTGTGCAGCTCGGGGACGAGGCCCGGCTGGTGGCATGTGCCTGGCCCCCGTT
PKD1P5          GAGGCAGTTTGTGCAGCTCGGGGACGAGGCCCGGCTGGTGGCATGTGCCTGGCCCCCGTT
PKD1            GAGGCAGTTTGTGCAGCTCGGGGACGAGGCCTGGCTGGTGGCATGTGCCTGGCCCCCGTT
PKD1P4          ------------------------------------------------------------
PKD1P1          ------------------------------------------------------------
PKD1P3          ------------------------------------------------------------
PKD1P6          ------------------------------------------------------------

                                                      c.4264
                                                        .
PKD1P2          CCCCTACCGCTACACCTGGGACTTTGGCACCGAAGAAGCCGTCCCCGCCCGTGTCGGGGG
PKD1P5          CCCCTACCGCTACACCTGGGACTTTGGCACCGAAGAAGCCGTCCCCGCCCGTGTCGGGGG
PKD1            CCCCTACCGCTACACCTGGGACTTTGGCACCGAGGAAGCCGCCCCCACCCGTGCCAGGGG
PKD1P4          ------------------------------------------------------------
PKD1P1          ------------------------------------------------------------
PKD1P3          ------------------------------------------------------------
```

```
PKD1P6          ------------------------------------------------------------

PKD1P2          CCCTGAGGTGACGTTCATCTACCGAGACCCAGGCTCCTATCTTGTGACAGTCACCGCGTC
PKD1P5          CCCTGAGGTGACGTTCATCTACCGAGACCCAGGCTCCTATCTTGTGACAGTCACCGCGTC
PKD1            CCCTGAGGTGACGTTCATCTACCGAGACCCAGGCTCCTATCTTGTGACAGTCACCGCGTC
PKD1P4          ------------------------------------------------------------
PKD1P1          ------------------------------------------------------------
PKD1P3          ------------------------------------------------------------
PKD1P6          ------------------------------------------------------------

PKD1P2          CAACAACATCTCCGCTGCCAATGACTCAGCCCTGGTGGAGGTGCAGGAGCCCGTGCTGGT
PKD1P5          CAACAACATCTCCGCTGCCAATGACTCAGCCCTGGTGGAGGTGCAGGAGCCCGTGCTGGT
PKD1            CAACAACATCTCTGCTGCCAATGACTCAGCCCTGGTGGAGGTGCAGGAGCCCGTGCTGGT
PKD1P4          ------------------------------------------------------------
PKD1P1          ------------------------------------------------------------
PKD1P3          ------------------------------------------------------------
PKD1P6          ------------------------------------------------------------

PKD1P2          CACCAGCATCAAGGTCAATGGCTCCCTTGGGCTGGAGCTGCAGTAGCCGTACCTGTTCTC
PKD1P5          CACCAGCATCAAGGTCAATGGCTCCCTTGGGCTGGAGCTGCAGCAGCCGTACCTGTTCTC
PKD1            CACCAGCATCAAGGTCAATGGCTCCCTTGGGCTGGAGCTGCAGCAGCCGTACCTGTTCTC
PKD1P4          ------------------------------------------------------------
PKD1P1          ------------------------------------------------------------
PKD1P3          ------------------------------------------------------------
PKD1P6          ------------------------------------------------------------

PKD1P2          TGCTGTGGGCCGTGGGCGCCCCGCCAGCTACCTGTGGGATCTGGGGGACGGTGGGCGGCT
PKD1P5          TGCTGTGGGCCGTGGGCGCCCCGCCAGCTACCTGTGGGATCTGGGGGACGGTGGGCGGCT
PKD1            TGCTGTGGGCCGTGGGCGCCCCGCCAGCTACCTGTGGGATCTGGGGGACGGTGGGTGGCT
PKD1P4          ------------------------------------------------------------
PKD1P1          ------------------------------------------------------------
PKD1P3          ------------------------------------------------------------
PKD1P6          ------------------------------------------------------------

PKD1P2          CGAGGGTCCGGAGGTCACCCACGCTTACAACAGCACAGGTGACTTCACCGTTAGGGTGGC
PKD1P5          CGAGGGTCCGGAGGTCACCCACGCTTACAACAGCACAGGTGACTTCACCGTTAGGGTGGC
PKD1            CGAGGGTCCGGAGGTCACCCACGCTTACAACAGCACAGGTGACTTCACCGTTAGGGTGGC
PKD1P4          ------------------------------------------------------------
PKD1P1          ------------------------------------------------------------
PKD1P3          ------------------------------------------------------------
PKD1P6          ------------------------------------------------------------

PKD1P2          CGGCTGCAATGAGGTGAGCCGCAGCGAGGCCTGGCTCAATGTGACGGTGAAGCGGCGCGT
PKD1P5          CGGCTGCAATGAGGTGAGCCGCAGCGAGGCCTGGCTCAATGTGACGGTGAAGCGGCGCGT
PKD1            CGGCTGGAATGAGGTGAGCCGCAGCGAGGCCTGGCTCAATGTGACGGTGAAGCGGCGCGT
PKD1P4          ------------------------------------------------------------
PKD1P1          ------------------------------------------------------------
PKD1P3          ------------------------------------------------------------
PKD1P6          ------------------------------------------------------------

PKD1P2          GCGGGGGCTCATCGTCAATGCCAGCTGCACGGTGGTGCCCCTGAATGGGAGCATGAGCTT
PKD1P5          GCGGGGGCTCATCGTCAATGCCAGCTGCACGGTGGTGCCCCTGAATGGGAGCATGAGCTT
PKD1            GCGGGGGCTCGTCGTCAATGCAAGCCGCACGGTGGTGCCCCTGAATGGGAGCGTGAGCTT
PKD1P4          ------------------------------------------------------------
PKD1P1          ------------------------------------------------------------
PKD1P3          ------------------------------------------------------------
PKD1P6          ------------------------------------------------------------

PKD1P2          CAGCACCTCGCTGGAGGCCGGCAGTGATGTGCGCTATTCCTGGGTGCTCTGTGACCGCTG
PKD1P5          CAGCACCTCGCTGGAGGCCGGCAGTGATGTGCGCTATTCCTGGGTGCTCTGTGACCGCTG
PKD1            CAGCACGTCGCTGGAGGCCGGCAGTGATGTGCGCTATTCCTGGGTGCTCTGTGACCGCTG
PKD1P4          ------------------------------------------------------------
PKD1P1          ------------------------------------------------------------
PKD1P3          ------------------------------------------------------------
PKD1P6          ------------------------------------------------------------

PKD1P2          CACGCCCATCTCTGGGGGTCCTGCCATCTCTTTACACCTTCCGCTCCGTGGGCACCTTCA
PKD1P5          CACGCCCATCTCTGGGGGTCCTGCCATCTCTTTACACCTTCCGCTCCGTGGGCACCTTCA
PKD1            CACGCCCATCCCTGGGGGTCCTACCATCTCTT-ACACCTTCCGCTCCGTGGGCACCTTCA
PKD1P4          ------------------------------------------------------------
PKD1P1          ------------------------------------------------------------
PKD1P3          ------------------------------------------------------------
PKD1P6          ------------------------------------------------------------

PKD1P2          ATATCATCGTCACAGCTGAGAACGAGGTGGGCTCCGCCCAGGACAGCATCTTCGTCTATG
PKD1P5          ATATCATCGTCACAGCTGAGAACGAGGTGGGCTCCGCCCAGGACAGCATCTTCGTCTATG
PKD1            ATATCATCGTCACGGCTGAGAACGAGGTGGGCTCCGCCCAGGACAGCATCTTCGTCTATG
PKD1P4          ------------------------------------------------------------
PKD1P1          ------------------------------------------------------------
PKD1P3          ------------------------------------------------------------
PKD1P6          ------------------------------------------------------------

PKD1P2          TCCTGCAGCTCATAGAGGGGCTGCAGGTGGTGGGCGGTGGCCGCTACTTCCCCACCAACC
PKD1P5          TCCTGCAGCTCATAGAGGGGCTGCAGGTGGTGGGCGGTGGCCGCTACTTCCCCACCAACC
PKD1            TCCTGCAGCTCATAGAGGGGCTGCAGGTGGTGGGCGGTGGCCGCTACTTCCCCACCAACC
PKD1P4          ------------------------------------------------------------
PKD1P1          ------------------------------------------------------------
```

```
PKD1P3         ------------------------------------------------------------
PKD1P6         ------------------------------------------------------------

PKD1P2         ACACGGTACAGCTGCAGGCCGTGGTCAGGGATGGCACCAAC---ATCTACAGCTGGACTG
PKD1P5         ACATGGTACAGCTGCAGGCCGTGGTCAGGGACGGCACCATC---TCCTACAGCTGGACTG
PKD1           ACACGGTACAGCTGCAGGCCGTGGTTAGGGATGGCACCAACGTCTCCTACAGCTGGACTG
PKD1P4         ------------------------------------------------------------
PKD1P1         ------------------------------------------------------------
PKD1P3         ------------------------------------------------------------
PKD1P6         ------------------------------------------------------------

PKD1P2         CCTGGAGGGACAGGGGCCCGGCCCTGGCCGGCAGCGGCAAAGGCTTCTCGCTCACTGCGC
PKD1P5         CCTGGAGGGACAGGGGCCCGGCCCTGGCCGGCAGCGGCAAAGGCTTCTCGCTCACCGCGC
PKD1           CCTGGAGGGACAGGGGCCCGGCCCTGGCCGGCAGCGGCAAAGGCTTCTCGCTCACCGTGC
PKD1P4         ------------------------------------------------------------
PKD1P1         ------------------------------------------------------------
PKD1P3         ------------------------------------------------------------
PKD1P6         ------------------------------------------------------------

PKD1P2         TCGAGGCCGGCACCTACCATGTGCAGCTGCGGGCCACCAACATGCTGGGCAGCGCCTGGG
PKD1P5         TCGAGGCCGGCTCCTACCATGTGCAGCTGCGGGCCACCAACATGCTGGGCAGCGCCTGGG
PKD1           TCGAGGCCGGCACCTACCATGTGCAGCTGCGGGCCACCAACATGCTGGGCAGCGCCTGGG
PKD1P4         ------------------------------------------------------------
PKD1P1         ------------------------------------------------------------
PKD1P3         ------------------------------------------------------------
PKD1P6         ------------------------------------------------------------

PKD1P2         CTGACTGCACCGTGGACTTCGTGGAGCCTGTGGGGTGGCTGATGGTGGCCGCCTCCCCGA
PKD1P5         CTGACTGCACCGTGGACTTCGTGGAGCCTGTGGGGTGGCTGATGGTGGCCGCCTCCCCGA
PKD1           CCGACTGCACCATGGACTTCGTGGAGCCTGTGGGGTGGCTGATGGTGGCCGCCTCCCCGA
PKD1P4         ------------------------------------------------------------
PKD1P1         ------------------------------------------------------------
PKD1P3         ------------------------------------------------------------
PKD1P6         ------------------------------------------------------------

PKD1P2         ACCCAGCTGCCGTCAACACAAGTGTCACCCTCAGTGCCGAGCTGGCTGGTGGCAGTGGTG
PKD1P5         ACCCAGCTGCCGTCAACACAAGCGTCACCCTCAGTGCCGAGCTGGCTGGTGGCAGTGGTG
PKD1           ACCCAGCTGCCGTCAACACAAGCGTCACCCTCAGTGCCGAGCTGGCTGGTGGCAGTGGTG
PKD1P4         ------------------------------------------------------------
PKD1P1         ------------------------------------------------------------
PKD1P3         ------------------------------------------------------------
PKD1P6         ------------------------------------------------------------
```

```
PKD1P2         TCGTATACACTTGGTCCTTGGAGGAGGGGCTGAGCTGGGAGACCCCCGAGCCATTTACCA
PKD1P5         TCGTATACACTTGGTCCTTGGAGGAGGGGCTGAGCTGGGAGACCCCCGAGCCATTTACCA
PKD1           TCGTATACACTTGGTCCTTGGAGGAGGGGCTGAGCTGGGAGACCTCCGAGCCATTTACCA
PKD1P4         ------------------------------------------------------------
PKD1P1         ------------------------------------------------------------
PKD1P3         ------------------------------------------------------------
PKD1P6         -----------------------------------------------ATGCCGCCCGCCG

PKD1P2         CCCACAGCTTCCCCACACCCGGCCTGCACTTGGTCACCATGACGGCAGGGAACCCGCTGG
PKD1P5         CCCACAGCTTCCCCACACCCGGCCTGCACTTGGTCACCATGACGGCAGGGAACCCGCTGG
PKD1           CCCATAGCTTCCCCACACCCGGCCTGCACTTGGTCACCATGACGGCAGGGAACCCGCTGG
PKD1P4         ------------------------------------------------------------
PKD1P1         ------------------------------------------------------------
PKD1P3         ------------------------------------------------------------
PKD1P6         CGCCCGCCCGCCTGGCGCTGGCCCTGGGCCTGGGC-CTGTGGCT-CGGGGCGCTGGCGGG

            c.5363
               .
PKD1P2         GCTCAGCCAACGCCACCGTGGAAGTGGATGTGCAGGTGCCTGTGAGTGGCCTCAGCATCA
PKD1P5         GCTCAGCCAACGCCACCGTGGAAGTGGATGTGCAGGTGCCTGTGAGTGGCCTCAGCATCA
PKD1           GCTCAGCCAACGCCACCGTGGAAGTGGATGTGCAGGTGCCTGTGAGTGGCCTCAGCATCA
PKD1P4         ------------------------------------------------------------
PKD1P1         ------------------------------------------------------------
PKD1P3         ------------------------------------------------------------
PKD1P6         GGGCCCCGGGCGCGGCTGCGGGCCTTG-CGAGCCCCCCTGCCTCTGCGGCCT-AGCGCCC

PKD1P2         GGGCCAGCGAGCCGGGAGGCAGCTTCGTGGCGGCCGGGTCCTCTGTGCCCTTTTGGGGGC
PKD1P5         GGGCCAGCGAGCCGGGAGGCAGCTTCGTGGCGGCCGGGTCCTCTGTGCCCTTTTGGGGGC
PKD1           GGGCCAGCGAGCCCGGAGGCAGCTTCGTGGCGGCCGGGTCCTCTGTGCCCTTTTGGGGGC
PKD1P4         ------------------------------------------------------------
PKD1P1         ------------------------------------------------------------
PKD1P3         ------------------------------------------------------------
PKD1P6         GGCGCCGCCTGCC--GCGTCAACTGCTCGGGCCGCGGGCTGCGGACGCTCGGTCCCGCGC

PKD1P2         AGCTGGCCACGGGCACCAATGTGAGCTGGTGCTGGGCTGTGCCCGGCGGCAGCAGCAAGC
PKD1P5         AGCTGGCCACGGGCACCAATGTGAGCTGGTGCTGGGCTGTGCCCGGCGGCAGCAGCAAGC
PKD1           AGCTGGCCACGGGCACCAATGTGAGCTGGTGCTGGGCTGTGCCCGGCGGCAGCAGCAAGC
PKD1P4         -------------------TGTGAGC----------------------------------
PKD1P1         -------------------TGTGAGC--GT------------------------------
PKD1P3         ------------------------------------------------------------
PKD1P6         TGCGCATCCCCGCGGACGCCACAGCGCTACGTCTCCCACAACCTGCTCCGGGCGCTGGAC

PKD1P2         GTGGCCCTCATGTCACCATGGTCTTCCCGGATGCTGGCACCTTCAACATCCGGCTCAATG
PKD1P5         GTGGCCCTCATGTCACCATGGTCTTCCCGGATGCTGGCACCTTCAACATCTGGCTCAATG
PKD1           GTGGCCCTCATGTCACCATGGTCTTCCCGGATGCTGGCACCTTCTCCATCCGGCTCAATG
```

```
PKD1P4       ------------------------------------------------------------
PKD1P1       ------------------------------------------------------------
PKD1P3       ------------------------------------------------------------
PKD1P6       GTTGGGCTCCTGGCGAACCTCTCGGCGCTGGCAGAGCTGGATATAAGCAACAACAAGATT

                  c.5611
                     .
PKD1P2       CCTCCAACGCAGTCAGCTGGGTCT-CAGCCACGTACAACCTCACGGTGGAGGAGCCCATC
PKD1P5       CCTCCAACGCAGTCAGCTGGGTCT-CAGCCACGTACAACCTCACGGTGGAGGAGCCCATC
PKD1         CCTCCAACGCAGTCAGCTGGGTCT-CAGCCACGTACAACCTCACGGCGGAGGAGCCCATC
PKD1P4       ------------------------------------------------------------
PKD1P1       ------------------------------------------------------------
PKD1P3       ------------------------------------------------------------
PKD1P6       TCTACGTTAGAAGAAGGAATATTTGCTAATTTATTTAATTTAAGTGAAATAAACCTGAGT

PKD1P2       GTGGGCCTGGTGCTGTGGGCCAGCAGCAAGGTGGTGGCGCCCGGGCAGCTTGTCCATTTT
PKD1P5       GTGGGCCTGGTGCTGTGGGCCAGCAGCAAGGTGGTGGCGCCCGGGCAGCTTGTCCATTTT
PKD1         GTGGGCCTGGTGCTGTGGGCCAGCAGCAAGGTGGTGGCGCCCGGGCAGCTGGTCCATTTT
PKD1P4       ------------------------------------------------------------
PKD1P1       ------------------------------------------------------------
PKD1P3       ------------------------------------------------------------
PKD1P6       GGGAACCCGTTTGAGTGTGACTGTGGCCTGGCGTGGCTGCCGCGATGGGCGGAGGAGCAG

PKD1P2       CAGATCCTGCTGGCTGCCGGCTCAGCTGTCACCTTCCGCCTGCAGGTCGGCGGGGCCAGC
PKD1P5       CAGATCCTGCTGGCTGCCGGCTCAGCTGTCACCTTCCGCCTGCAGGTCGGCGGGGCCAGC
PKD1         CAGATCCTGCTGGCTGCCGGCTCAGCTGTCACCTTCCGCCTGCAGGTCGGCGGGGCCAAC
PKD1P4       ------------------------------------------------------------
PKD1P1       ------------------------------------------------------------
PKD1P3       ------------------------------------------------------------
PKD1P6       CAGGTGCGGGTGGTGCAGCCCGAGGCAGCCACGTGTGCTGGGCCTGACTCCCTGGCTGGC

          c.5783
              .
PKD1P2       CCCGAAGTGCTCCCTGGGCCCCGTTTCTCCCACAGCTTCCCCCGCATCGGAGACCACGTG
PKD1P5       CCCGAAGTGCTCCCTGGGCCCCGTTTCTCCCACAGCTTCCCCCGCATCGGAGACCACGTG
PKD1         CCCGAGGTGCTCCCCGGGCCCCGTTTCTCCCACAGCTTCCCCCGCGTCGGAGACCACGTG
PKD1P4       ------------------------------------------------------------
PKD1P1       ------------------------------------------------------------
PKD1P3       ------------------------------------------------------------
PKD1P6       C---AGCCTCTGCTTGGCATCCCCTTGCTGGACAGTGGCTGTGGT----GAGAGTATGTC

PKD1P2       GTGAGCGTGCAGGGCAAAAACCACGTGAGCTGGGCCCAGGCGCAGGTGCGCATCGTGGTG
PKD1P5       GTGAGCGTGCAGGGCAAAAACCACGTGAGCTGGGCCCAGGCGCAGGTGCGCATCGTGGTG
PKD1         GTGAGCGTGCGGGGCAAAAACCACGTGAGCTGGGCCCAGGCGCAGGTGCGCATCGTGGTG
PKD1P4       ------------------------------------------------------------
PKD1P1       ------------------------------------------------------------
PKD1P3       ------------------------------------------------------------
PKD1P6       GCCTGCCTCCCTGACAACAGCT--------CAGGCACCGTGGCAGCAGTGTCCTTTTCAG

PKD1P2       CTGGAGGCCGTGAGCGGGCTGCAGGTGCCCAACTGCTGTGAGCCTGGCATCGCCATGGGC
PKD1P5       CTGGAGGCCGTGAGCGGGCTGCAGGTGCCCAACTGCTGTGAGCCTGGCATCGCCATGGGC
PKD1         CTGGAGGCCGTGAGTGGGCTGCAGGTGCCCAACTGCTGCGAGCCTGGCATCGCCACGGGC
PKD1P4       ------------------------------------------------------------
PKD1P1       ------------------------------------------------------------
PKD1P3       ------------------------------------------------------------
PKD1P6       CTGCCCAC-GAAGGCCTGCTTCAGCCAGAGGCCTGCAGCGCCTTCTGCTTCTCCACCGGC

              c.5964
                  .
PKD1P2       ACTGAGAGGAACTTCACAGCCCGCGTGCAGCGCGGCTCTCGGGTCGCCTACGCCTGGTAC
PKD1P5       ACTGAGAGGAACTTCACAGCCCGCGTGCAGCGCGGCTCTCGGGTCGCCTACGCCTGGTAC
PKD1         ACTGAGAGGAACTTCACAGCCCGCGTGCAGCGCGGCTCTCGGGTCGCCTACGCCTGGTAC
PKD1P4       ------------------------------------------------------------
PKD1P1       ------------------------------------------------------------
PKD1P3       ------------------------------------------------------------
PKD1P6       C------AGGGCCTCGCAGCCCTCTCGGAGCAGGGCT-----GGTGCCTGTGTGGGGCGG

PKD1P2       TTCTCGCTGCAGAAGGTCCGGGGCGACTCTCTGTTCATCCTGTCGGGCCGCGACGTCACC
PKD1P5       TTCTCGCTGCAGAAGGTCCGGGGCGACTCTCTGTTCATCCTGTCGGGCCGCGACGTCACC
PKD1         TTCTCGCTGCAGAAGGTCCAGGGCGACTCGCTGGTCATCCTGTCGGGCCGCGACGTCACC
PKD1P4       ------------------------------------------------------------
PKD1P1       ------------------------------------------------------------
PKD1P3       ------------------------------------------------------------
PKD1P6       CCC----------AGCCCTCTAGTGCCTCCTTCGCCTGCCTGTCCCTCTGCTCCGGCCCC

PKD1P2       TACACGCC-GTGGCCGCGGGGCTGTTGGAGATCCAGGTGCGTGCCTTCAACGCCCTGGGC
PKD1P5       TACACGCC-GTGGCCGCGGGGCTGTTGGAGATCCAGGTGCGTGCCTTCAACGCCCTGGGC
PKD1         TACACGCCCGTGGCCGCGGGGCTGTTGGAGATCCAGGTGCGCGCCTTCAACGCCCTGGGC
PKD1P4       ------------------------------------------------------------
PKD1P1       ------------------------------------------------------------
PKD1P3       ------------------------------------------------------------
PKD1P6       CCGCCGCCTCCTGCCCCCAC-CTGTAGGGGCCCCA----CCCTCCTCCA-----------

PKD1P2       AGTGAGAACCGCACGCTGGTGCTGGAGGTTCAGGACGCCGTCCAGTATGTGGCCCTGCGG
PKD1P5       AGTGAGAACCGCACGCTGGTGCTGGAGGTTCAGGACGCCGTCCAGTATGTGGCCCTGCGC
PKD1         AGTGAGAACCGCACGCTGGTGCTGGAGGTTCAGGACGCCGTCCAGTATGTGGCCCTGCAG
PKD1P4       ------------------------------------------------------------
PKD1P1       ------------------------------------------------------------
PKD1P3       ------------------------------------------------------------
PKD1P6       ----------GCACGTCTTCCCTGCCTCCCCAGGGGCCCCCCTGG--TGGGGCCCCACGG
```

```
PKD1P2      AGCGGCCCCTGCTTCACCAACCGCTTGGCGCAGTTTGAGGCCGCCACCAGCCCCAGCCCC
PKD1P5      AGCGGCCCCTGCTTCACCAACCGCTCGGCGCAGTTTGAGGCCGCCACCAGCCCCAGCCCC
PKD1        AGCGGCCCCTGCTTCACCAACCGCTCGGCGCAGTTTGAGGCCGCCACCAGCCCCAGCCCC
PKD1P4      ------------------------------------------------------------
PKD1P1      ------------------------------------------------------------
PKD1P3      ------------------------------------------------------------
PKD1P6      A----CCCCTGGC-CTCCGGCCAGCTAGCAGCCTTCCACATCGCT----GCCCCGCTCCC

PKD1P2      CGGCGCGTGGCCTACCACTGGGACTTTGGGGATGGGTCCCCAGGGCAGGACACAGATAAG
PKD1P5      CGGCGCGTGGCCTACCACTGGGACTTTGGGGATGGGTCCCCAGGGCAGGACACAGATGAG
PKD1        CGGCGTGTGGCCTACCACTGGGACTTTGGGGATGGGTCGCCAGGGCAGGACACAGATGAG
PKD1P4      ------------------------------------------------------------
PKD1P1      ------------------------------------------------------------
PKD1P3      ------------------------------------------------------------
PKD1P6      TGTCACT--GCCACACGCTGGGACTTCGGAGACGGCTCCCCCGAGGTGGATGCCGCTGGG

PKD1P2      CCCAGGGCCGAGCACTCCTACCTGAGGCCTGGGGACTACCGCGTGCAGGTGAACGCCTCC
PKD1P5      CCCAGGGCCGAGCACTCCTACCTGAGGCCTGGGGACTACCGCGTGCAGGTGAACGCCTCC
PKD1        CCCAGGGCCGAGCACTCCTACCTGAGGCCTGGGGACTACCGCGTGCAGGTGAACGCCTCC
PKD1P4      ------------------------------------------------------------
PKD1P1      ------------------------------------------------------------
PKD1P3      ------------------------------------------------------------
PKD1P6      CCGGCTGCCTCGCATCGCTATGTGCTGCCTGGGCGCTATCACGTGACGGC---CGTGCTG

PKD1P2      AACCTGGTGAGCTTTTTCGTGGCGCAGGCCACGGTGACCGTCCAGGTGCTGGCCTGCCGG
PKD1P5      AACCTGGTGAGCTTTTTCGTGGCGCAGGCCACGGTGACCGTCCAGGTGCTGGCCTGCCGG
PKD1        AACCTGGTGAGCTTCTTCGTGGCGCAGGCCACGGTGACCGTCCAGGTGCTGGCCTGCCGG
PKD1P4      ------------------------------------------------------------
PKD1P1      ------------------------------------------------------------
PKD1P3      ------------------------------------------------------------
PKD1P6      GCCCTGGGGGCCGGCTCAGCCCTGCTGGGGACAGA---CGTGCAGGTGGAAGCGGCACCT

PKD1P2      GAGCCGGAGGTGGACGTGGTCCTGCCCCTGCAGGTGCTGATGCGACGATCACAGCGCAAC
PKD1P5      GAGCCGGAGGTGGACGTGGTCCTGCCCCTGCAGGTGCTGATGCGACGATCACAGCGCAAC
PKD1        GAGCCGGAGGTGGACGTGGTCCTGCCCCTGCAGGTGCTGATGCGGCGATCACAGCGCAAC
PKD1P4      ------------------------------------------------------------
PKD1P1      ------------------------------------------------------------
PKD1P3      ------------------------------------------------------------
PKD1P6      GCCGCCCTGGAGCTCGTGTGCCCGTCCTCG-GTGCAGAGTGACGAGAGCCTCGACCTCAG

PKD1P2      TGCCTGGATGCCTACGTTGACCTGCGCGACTGTGTCACCTACCAGACTGAGTACCGCTGG
PKD1P5      TGCCTGGATGCCTACGTTGACCTGCGCGACTGTGTCACCTACCAGACTGAGTACCGCTGG
PKD1        TACTTGGAGGCCCACGTTGACCTGCGCGACTGCGTCACCTACCAGACTGAGTACCGCTGG
PKD1P4      ------------------------------------------------------------
PKD1P1      ------------------------------------------------------------
PKD1P3      ------------------------------------------------------------
PKD1P6      CATCCAGAA--CCGCGGTGGTTCAGGCCTGGAGGCTGCCTACAGCATCG--TGGCCCTGG

PKD1P2      GAGGTGTACCGCACCGCCAGCTGCCAGCGGCCGGGGTGCCCGGCGCGTGTGGCCCTGCCC
PKD1P5      GAGGTGTACCGCACCGCCAGCTGCCAGCGGCCGGGGCGCCCGGCGCGTGTGGCCCTGCCC
PKD1        GAGGTGTATCGCACCGCCAGCTGCCAGCGGCCGGGGCGCCCAGCGCGTGTGGCCCTGCCC
PKD1P4      ------------------------------------------------------------
PKD1P1      ------------------------------------------------------------
PKD1P3      ------------------------------------------------------------
PKD1P6      GCGAGGAGCCGGCCCGAGCGGTGCACCCGCTCTGCCCCTCGGACACG-GAGATCTTCTCT

PKD1P2      GGCGTGGACGTGAGCCGGCCTCAGCTGGTGC------TGCCGCGGCTGGCGCTGCCTGTG
PKD1P5      GGCGTGGACGTGAGCCGGCCTCAGCTGGTGC------TGCCGTGGCTGGCGCTGCCTGTG
PKD1        GGCGTGGACGTGAGCCGGCCTCGGCTGGTGC------TGCCGCGGCTGGCGCTGCCTGTG
PKD1P4      ------------------------------------------------------------
PKD1P1      ------------------------------------------------------------
PKD1P3      ------------------------------------------------------------
PKD1P6      GGCAACGGGCACTGCTACCGCCTGGTGGTGGAGAAGGCGGCCTGGCTGCAGGCGCAGGAG

PKD1P2      GGGCACTA---CTGCTTTGTGTTTGTCGTGTCATTTGGGGACACGCCACTGGCACGGAGC
PKD1P5      GGGCACTA---CTGCTTTGTGTTTGTCGTGTCATTTGGGGACACGCCACTGGCACGGAGC
PKD1        GGGCACTA---CTGCTTTGTGTTTGTCGTGTCATTTGGGGACACGCCACTGACACAGAGC
PKD1P4      ------------------------------------------------------------
PKD1P1      ------------------------------------------------------------
PKD1P3      ------------------------------------------------------------
PKD1P6      CAGTGTCGGGCCTGGGCCGGGGCCGCCCTGGCAATGGTGGACAGTCCCGCCGTGCAGCGC

PKD1P2      ATCCAGGCCAATGTGACGGT--GGCCCCCGAGCGCCTGGTGCCCATCACTGAGGGTGGCT
PKD1P5      ATCCAGGCCAATGTGACGGT--GGCCCCCGAGCGCCTGGTGCCCATCACTGAGGGTGGCT
PKD1        ATCCAGGCCAATGTGACGGT--GGCCCCCGAGCGCCTGGTGCCCATCATTGAGGGTGGCT
PKD1P4      ------------------------------------------------------------
PKD1P1      ------------------------------------------------------------
PKD1P3      ------------------------------------------------------------
PKD1P6      TTCCTGGTCTCCCGGGTCACCAGGAGCCTAGACGTGTGGATCGGCTTCTCGACTGTGCAG

PKD1P2      CCTACCGCG-TGTGGTCAGACACACAGGACCTGGTGCTGGATGGGAGCGAGTCCTACGAC
PKD1P5      CCTACCGCG-TGTGGTCAGACACACAGGACCTGGTGCTGGATGGGAGCGAGTCCTACGAC
PKD1        CATACCGCG-TGTGGTCAGACACACGGGACCTGGTGCTGGATGGGAGCGAGTCCTACGAC
PKD1P4      ------------------------------------------------------------
PKD1P1      ------------------------------------------------------------
PKD1P3      ------------------------------------------------------------
```

```
PKD1P6          CGGGTGGAGGTGGGCCCAGCGCCGCAGGGCGAGGCCTTCAGCCTGGAGAGCTGCCAGAAC

PKD1P2          CC-CAACCTGGAGGACGGCGACCAGACGCCGCTCAGTTTCCAGTGGGCCTGTGTGGCTTC
PKD1P5          CC-CAACCTGGAGGACGGCGACCAGACGCCGCTCAGTTTCCAGTGGGCCTGTGTGGCTTC
PKD1            CC-CAACCTGGAGGACGGCGACCAGACGCCGCTCAGTTTCCACTGGGCCTGTGTGGCTTC
PKD1P4          ------------------------------------------------------------
PKD1P1          ------------------------------------------------------------
PKD1P3          ------------------------------------------------------------
PKD1P6          TGGCTGCCCGGGGAGCCACACCCAGCCACAGCCGAG----CACTGCATCTGGCTCGGGCC

PKD1P2          GACACAGAGGGAGGCTGGCGGGTGTGCGCTGAACTTTGGGCCCCGCGGGAGCAGCACGGT
PKD1P5          GACACAGAGGGAGGCTGGCGGGTGTGCGCTGAACTTTGGGCCCCGCGGGAGCAGCACGGT
PKD1            GACACAGAGGGAGGCTGGCGGGTGTGCGCTGAACTTTGGGCCCCGCGGGAGCAGCACGGT
PKD1P4          ------------------------------------------------------------
PKD1P1          ------------------------------------------------------------
PKD1P3          ------------------------------------------------------------
PKD1P6          --CACCAAGGGAGGCTGGCGGGTGTGCGCTGAACTTTGGGCCCCGCGGGAGCAGCACGGT

                                    c.6992
                                       .
PKD1P2          CACCATTCCACGGGAACGGCTGGCAGCTGGCGTGGAGTACACCTTCAGCCTCACCGTGTG
PKD1P5          CACCATTCCACGGGAACGGCTGGCAGCTGGCGTGGAGTACACCTTCAGCCTCACCGTGTG
PKD1            CACCATTCCACGGGAGCGGCTGGCGGCTGGCGTGGAGTACACCTTCAGCCTGACCGTGTG
PKD1P4          ------------------------------------------------------------
PKD1P1          ------------------------------------------------------------
PKD1P3          ------------------------------------------------------------
PKD1P6          CACCATTCCACGGGAACGGCTGGCGGCTGGCGTGGAGTACACCTTCAGCCTCACCGTGTG

PKD1P2          GAAGGCCGGCCACAAGGAGGAGGCCACCAACCAGACGGTGCTGATCCGCAGTGGCCGGGT
PKD1P5          GAAGGCCGGCCGCAAGGAGGAGGCCACCAACCAGACGGTGCTGATCCGGAGTGGCCGGGT
PKD1            GAAGGCCGGCCGCAAGGAGGAGGCCACCAACCAGACGGTGCTGATCCGGAGTGGCCGGGT
PKD1P4          -------------------------------------GTGCTGATCCGCAGTGGCCGGGT
PKD1P1          -------------------------------------GTGCTGATCCGCAGTGGCCGGGT
PKD1P3          -------------------------------------GTGCTGATCCGCAGTGGCCGGGT
PKD1P6          GAAGGCCGGCCGCAAGGAGGAGGCCACCAACCAGACGGTGCTGATCCGCAGTGGCCGGGT
                                                     *********** ***********

PKD1P2          GCCCATTGTGTCCTTGGAGTGTGTGTCCTGCAAGGCACAGGCCGTGTACGAAGTGAGCCG
PKD1P5          GCCCATTGTGTCCTTGGAGTGTGTGTCCTGCAAGGCACAGGCCGTGTACGAAGTGAGCCG
PKD1            GCCCATTGTGTCCTTGGAGTGTGTGTCCTGCAAGGCACAGGCCGTGTACGAAGTGAGCCG
PKD1P4          GCCCATTGTGTCCTTGGAGTGTGTGTCCTGCAAGGCACAGGCCGTGTACGAAGTGAGCCG
PKD1P1          GCCCATTGTGTCCTTGGAGTGTGTGTCCTGCAAGGCACAGGCCGTGTACGAAGTGAGCCG
PKD1P3          GCCCATTGTGTCCTTGGAGTGTGTGTCCTGCAAGGCACAGGCCGTGTACGAAGTGAGCCG
PKD1P6          GCCCATTGTGTCCTTGGAGTGTGTGTCCTGCAAGGCACAGGCCGTGTACGAAGTGAGCCG
                ************************************************************
```

```
PKD1P2          CAGCTCCTACGTGTACCTGGAGGGCCGCTGCCTCAATTGCAGCAGCGGCTCCAAGCGAGG
PKD1P5          CAGCTCCTACGTGTACCTGGAGGGCCGCTGCCTCAATTGCAGCAGCGGCTCCAAGCGAGG
PKD1            CAGCTCCTACGTGTACTTGGAGGGCCGCTGCCTCAATTGCAGCAGCGGCTCCAAGCGAGG
PKD1P4          CAGCTCCTACGTGTACCTGGAGGGCCGCTGCCTCAATTGCAGCAGCGGCTCCAAGCGAGG
PKD1P1          CAGCTCCTACGTGTACCTGGAGGGCCGCTGCCTCAATTGCAGCAGCGGCTCCAAGCGAGG
PKD1P3          CAGCTCCTACGTGTACCTGGAGGGCCGCTGCCTCAATTGCAGCAGCGGCTCCAAGCGAGG
PKD1P6          CAGCTCCTACGTGTACCTGGAGGGCCGCTGCCTCAATTGCAGCAGCGGCTCCAAGCGAGG
                **************** *******************************************

PKD1P2          GCGGTGGGCTGCACGTACGTTCAGCAACAAGACGCTGGTGCTGGATGAGACCACCACATC
PKD1P5          GCGGTGGGCTGCACGTACGTTCAGCAACAAGACACTGGTGCTGGATGAGACCACCACATC
PKD1            GCGGTGGGCTGCACGTACGTTCAGCAACAAGACGCTGGTGCTGGATGAGACCACCACATC
PKD1P4          GCGGTGGGCTGCACGTACGTTCAGCAACAAGACGCTGGTGCTGGATGAGACCACCACATC
PKD1P1          GCGGTGGGCTGCACGTACGTTCAGCAACAAGACGCTGGTGCTGGATGAGACCACCACATC
PKD1P3          GCGGTGGGCTGCACGTACGTTCAGCAACAAGACGCTGGTGCTGGATGAGACCACCACATC
PKD1P6          GCGGTGGGCTGCACGTACGTTCAGCAACAAGACACTGGTGCTGGATGAGACCACCACATC
                ********************************* **************************

PKD1P2          CACGGGCAGCGCAGGCATGTGACTGGTGCTGCGGCGGGGCGTGCTGCGGGACGGCGAGGG
PKD1P5          CACGGGCAGCGCAGGCATGTGACTGGTGCTGCGGCGGGGCGTGCTGCGGGACGGCGAGGG
PKD1            CACGGGCAGTGCAGGCATGCGACTGGTGCTGCGGCGGGGCGTGCTGCGGGACGGCGAGGG
PKD1P4          CACGGGCAGCGCAGGCATGTGACTGGTGCTGCGGCGGGGCGTGCTGCGGGACGGCGAGGG
PKD1P1          CACGGGCAGCGCAGGCATGTGACTGGTGCTGCGGCGGGGCGTGCTGCGGGACGGCGAGGG
PKD1P3          CACGGGCAGCGCAGGCATGTGACTGGTGCTGCGGCGGGGCGTGCTGCGGGACGGCGAGGG
PKD1P6          CACGGGCAGCGCAGGCATGTGACTGGTGCTGCGGCGGGGCGTGCTGCGGGACGGCGAGGG
                ********* ********* ****************************************

PKD1P2          ATACACCTTCACGCTGACGGTGCTGGGCCGCTCTGGCGAGGAGGAGGGCTGCGCCTCCAT
PKD1P5          ATACACCTTCACGCTGACGGTGCTGGGCCGCTCTGGCGAGGAGGAGGGCTGTGCCTCCAT
PKD1            ATACACCTTCACGCTCACGGTGCTGGGCCGCTCTGGCGAGGAGGAGGGCTGCGCCTCCAT
PKD1P4          ATACACCTTCACGCTGACGGTGCTGGGCCGCTCTGGCAAGGAGGAGGGCTGCGCCTCCAT
PKD1P1          ATACACCTTCACGCTGACGGTGCTGGGCCGCTCTGGCGAGGAGGAGGGCTGCGCCTCCAT
PKD1P3          ATACACCTTCACGCTGACGGTGCTGGGCCGCTCTGGCGAGGAGGAGGGCTGCGCCTCCAT
PKD1P6          ATACACCTTCACGCTGACGGTGCTGGGCCGCTCTGGCGAGGAGGAGGGCTGCGCCTCCAT
                *************** ********************* ************* ********

PKD1P2          CCCCCTGTCCCCCAACCGCCCGCCGCTGGGGGGCTCTTGCTGCCTCTTCCCACTGGGCGC
PKD1P5          CCCCCTGTCCCCCAACCGCCCGCCGCTGGAGGGCTCTTGCCGCCTCTTCCCACTGGGCGC
PKD1            CCGCCTGTCCCCCAACCGCCCGCCGCTGGGGGGCTCTTGCCGCCTCTTCCCACTGGGCGC
PKD1P4          CCCCCTGTCCCCCAACCGCCCGCCGCTGGGGGGCTCTTGCCGCCTCTTCCCACTGGGCGC
PKD1P1          CCCCCTGTCCCCCAACCGCCCGCCGCTGGGGGGCTCTTGCTGCCTCTTCCCACTGGGCGC
PKD1P3          CCCCCTGTCCCCCAACCGCCCGCCGCTGGGGGGCTCTTGCCGCCTCTTCCCACTGGGCGC
PKD1P6          CCCCCTGTCCCCCAACCGCCCGCCGCTGGGGGGCTCTTGCCGCCTCTTCCCACTGGGCGC
                ** ************************** ********** *******************

PKD1P2          TGTGCACGCCCTCACCACCAAGGTGCACTTCAAATGCACGGGCTGGCATGACGCGGAGGA
PKD1P5          TGTGCACGCCCTCACCACCAAGGTGCACTTCGAATGCACGGGCTGGCATGACGCGGAGGA
PKD1            TGTGCACGCCCTCACCACCAAGGTGCACTTCGAATGCACGGGCTGGCATGACGCGGAGGA
PKD1P4          TGTGCACGCTCTCACCACCAAGGTGCACTTCGAATGCATGGGCTGGCATGACGCGGAGGA
```

```
PKD1P1      TGTGCACGCTCTCACCACCAAGGTGCACTTCGAATGCATGGGCTGGCATGACGCGGAGGA
PKD1P3      TGTGCACGCTCTCACCACCAAGGTGCACTTCGAATGCATGGGCTGGCATGACGCGGAGGA
PKD1P6      TGTGCACGCCCTCACCACCAAGGTGCACTTCGAATGCACGGGCTGGCATGACGCGGAGGA
            ********* ********************* ****** *********************

PKD1P2      TGCTGGCGCCCCGCTGGTGTATGCCCTGCTGCAGCGCTGTTGCCAGGGCCACTGCAA
PKD1P5      TGCTGGCGCCCCGCTGGTGTACGCCCTGCTGCAGCGCTGTTGCCAGGGCCACTGCAA
PKD1        TGCTGGCGCCCCGCTGGTGTACGCCCTGCTGCGGCGCTGTCGCCAGGGCCACTGCGA
PKD1P4      TGCTGGCGCCCCGCTGGTGTACGCCCTGCTGCAGCGCTGTCGCCAGGGCCACTGCGA
PKD1P1      TGCTGGCGCCCCGCTGGTGTACGCCCTGCTGCAGCGCTGTCGCCAGGGCCACTGCGA
PKD1P3      TGCTGGCGCCCCGCTGGTGTACGCCCTGCTGCAGCGCTGTCGCCAGGGCCACTGCGA
PKD1P6      TGCTGGCGCCCCGCTGGTGTACGCCCTGCTGCAGCGCTGTCGCCAGGGCCACTGCGA
            ********************* ************* ******* ************** *

PKD1P2      AGAGTTCTGTGTCTACAAGAGCAGCCTCTCCGGCTACGGAGCCGTGCTGCCCCCGGGTTT
PKD1P5      AGAGTTCTGTGTCTACAAGAGCAGCCTCTCCGGCTACGGAGCCGTGCTGCCCCCGGGTTT
PKD1        GGAGTTCTGTGTCTACAAGGGCAGCCTCTCCAGCTACGGAGCCGTGCTGCCCCCGGGTTT
PKD1P4      GGAGTTCTGTGTCTACAAGGGCAGCCTCTCCGGCTACGGAGCCGTGCTGCCCCCGGGTTT
PKD1P1      GGAGTTCTGTGTCTACAAGGGCAGCCTCTCCGGCTACGGAGCCGTGCTGCCCCCGGGTTT
PKD1P3      GGAGTTCTGTGTCTACAAGGGCAGCCTCTCCGGCTACGGAGCCGTGCTGCCCCCGGGTTT
PKD1P6      GGAGTTCTGTGTCTACAAGGGCAGCCTCTCCGGCTACGGAGCCGTGCTGCCCCCGGGTTT
             ****************** *********** ****************************

PKD1P2      CAGGCCACACTTCGAGGTGGGCCTGGCCGTGGTGGTGCAGGACCAGCTGGGAGCCGCTGT
PKD1P5      CAGGCCACACTTCGAGGTGGGCCTGGCCGTGGTGGTGCAGGACCAGCTGGGAGCCGCTGT
PKD1        CAGGCCACACTTCGAGGTGGGCCTGGCCGTGGTGGTGCAGGACCAGCTGGGAGCCGCTGT
PKD1P4      CAGGCCACACTTCGAGGTGGGCCTGGCCGTGGTGGTGCAGGACCAGCTGGGAGCCGCTGT
PKD1P1      CAGGCCACACTTCGAGGTGGGCCTGGCCGTGGTGGTGCAGGACCAGCTGGGAGCCGCTGT
PKD1P3      CAGGCCACACTTCGAGGTGGGCCTGGCCGTGGTGGTGCAGGACCAGCTGGGAGCCGCTGT
PKD1P6      CAGGCCACACTTCGAGGTGGGCCTGGCCGTGGTGGTGCAGGACCAGCTGGGAGCCGCTGT
            ************************************************************

PKD1P2      GGTCGCCCTCAACAGGTCTCTGGCCATCACCCTCCCAGAGCCCAACGGCAGCGCAATGGG
PKD1P5      GGTCGCCCTCAACAGGTCTCTGGCCATCACCCTCCCAGAGCCCAACGGCAGCGCAATGGG
PKD1        GGTCGCCCTCAACAGGTCTTTGGCCATCACCCTCCCAGAGCCCAACGGCAGCGCAACGGG
PKD1P4      GGTCGCCCTCAACAGGTCTCTGGCCATCACCCTCCCAGAGCCCAACGGCAGCGCAATGGG
PKD1P1      GGTCGCCCTCAACAGGTCTCTGGCCATCACCCTCCCAGAGCCCAACGGCAGCGCAATGGG
PKD1P3      GGTCGCCCTCAACAGGTCTCTGGCCATCACCCTCCCAGAGCCCAACGGCAGCGCAATGGG
PKD1P6      GGTCGCCCTCAACAGGTCTCTGGCCATCACCCTCCCAGAGCCCAACGGCAGCGCAATGGG
            ******************* ************************************ ***

PKD1P2      GCTCACAGTCTGGCTGCACCGGCTCACCGCTAGTGTGCTCCCGGGGCTGCTGCGGCAGGC
PKD1P5      GCTCACAGTCTGGCTGCACGGGCTCACCGCTAGTGTGCTCCCGGGGCTGCTGCGGCAGGC
PKD1        GCTCACAGTCTGGCTGCACGGGCTCACCGCTAGTGTGCTCCCAGGGCTGCTGCGGCAGGC
PKD1P4      GCTCACAGTCTGGCTGCACGGGCTCACCGCTAGTGTGCTCCCGGGGCTGCTGCGGCAGGC
PKD1P1      GCTCACAGTCTGGCTGCACGGGCTCACCGCTAGTGTGCTCCCGGGGCTGCTGCGGCAGGC
PKD1P3      GCTCACAGTCTGGCTGCACGGGCTCACCGCTAGTGTGCTCCCGGGGCTGCTGCGGCAGGC
PKD1P6      GCTCACAGTCTGGCTGCACGGGCTCACCGCTAGTGTGCTCCCGGGGCTGCTGCGGCAGGC
            ******************* ********************** *****************

PKD1P2      CGATCCCCAGCACGTCATCGAGTACTCGCTGGCCCTGGTCACTGTGCTGAACGAGTACGA
PKD1P5      CGATCCCCAGCACGTCATCGAGTACTCGCTGGCCCTGGTCACTGTGCTGAACGAGTACGA
PKD1        CGATCCCCAGCACGTCATCGAGTACTCGTTGGCCCTGGTCACCGTGCTGAACGAGTACGA
PKD1P4      CGATCCCCAGCACGTCATCGAGTACTCGCTGGCCCTGGTCACTGTGCTGAACGAGTACGA
PKD1P1      CGATCCCCAGCACGTCATCGAGTACTCGCTGGCCCTGGTCACTGTGCTGAACGAGTACGA
PKD1P3      CGATCCCCAGCACGTCATCGAGTACTCGCTGGCCCTGGTCACTGTGCTGAACGAGTACGA
PKD1P6      CGATCCCCAGCACGTCATCGAGTACTCGCTGGCCCTGGTCACCGTCCTGAACGAGTACGA
            **************************** ************* ** **************

PKD1P2      GCGGGCCCTGGACGTGGCGGCAGAGCCCAAGCACGAGCGGCAGCGCCGAGCCCAGATACG
PKD1P5      GCGGGCCCTGGACGTGGCGGCAGAGCCCAAGCACGAGCGGCAGCGCCGAGCCCAGATACG
PKD1        GCGGGCCCTGGACGTGGCGGCAGAGCCCAAGCACGAGCGGCAGCACCGAGCCCAGATACG
PKD1P4      GCGGGCCCTGGACGTGGCGGCAGAGCCCAAGCACGAGCGGCAGCGCCGAGCCCAGATACG
PKD1P1      GCGGGCCCTGGACGTGGCGGCAGAGCCCAAGCACGAGCGGCAGCGCCGAGCCCAGATACG
PKD1P3      GCGGGCCCTGGACGTGGCGGCAGAGCCCAAGCACGAGCGGCAGCGCCGAGCCCAGATACG
PKD1P6      GCGGGCCCTGGACGTGGCGGCAGAGCCCAAGCACGAGCGGCAGCGCCGAGCCCAGATACG
            ******************************************** ***************

PKD1P2      CAAGAACATCACGGAGACTCTGGTGTCCCTGAGGGTCCACACTGTGGATGACATCCAGCA
PKD1P5      CAAGAACATCACGGAGACTCTGGTGTCCCTGAGGGTCCACACTGTGGATGACATCCAGCA
PKD1        CAAGAACATCACGGAGACTCTGGTGTCCCTGAGGGTCCACACTGTGGATGACATCCAGCA
PKD1P4      CAAGAACATCACGGAGACTCTGGTGTCCCTGAGGGTCCACACTGTGGATGACATCCAGCA
PKD1P1      CAAGAACATCACGGAGACTCTGGTGTCCCTGAGGGTCCACACTGTGGATGACATCCAGCA
PKD1P3      CAAGAACATCACGGAGACTCTGGTGTCCCTGAGGGTCCACACTGTGGATGACATCCAGCA
PKD1P6      CAAGAACATCACGGAGACTCTGGTGTCCCTGAGGGTCCACACTGTGGATGACATCCAGCA
            ************************************************************

PKD1P2      GATCGCTGCTGCGCTGGCCCAGTGCATGG-------------------------------
PKD1P5      GATCGCTGCTGCGCTGGCCCAGTGCATGG-------------------------------
PKD1        GATCGCTGCTGCGCTGGCCCAGTGCATGGGGCCCAGCAGGGAGCTCGTATGCCGCTCGTG
PKD1P4      GATCGCTGCTGCGCTGGCCCAGTGCATGGGGCCCAGCAGGGAGCTCGTATGCCGCTCGTG
PKD1P1      GATCGCTGCTGCGCTGGCCCAGTGCATGGGGCCCAGCAGGGAGCTCGTATGCCGCTCGTG
PKD1P3      GATCGCTGCTGCGCTGGCCCAGTGCATGGGGCCCAGCAGGGAGCTCGTATGCCGCTCGTG
PKD1P6      GATCGCTGCTGCGCTGGCCCAGTGCATGG-------------------------------
            *****************************

PKD1P2      ------------------------------------------------------------
PKD1P5      ------------------------------------------------------------
PKD1        CCTGAAGCAGACGCTGCACAAGCTGGAGGCCATGATGCTCATCCTGCAGGCAGAGACCAC
PKD1P4      CCTGAAGCAGACGCTGCACAAGCTGGAGGCCATGATGCGCATCCTGCAGGCAGAGACCAC
PKD1P1      CCTGAAGCAGACGCTGCACAAGCTGGAGGCCATGATGCGCATCCTGCAGGCAGAGACCAC
PKD1P3      CCTGAAGCAGACGCTGCACAAGCTGGAGGCCATGATGCGCATCCTGCAGGCAGAGACCAC
PKD1P6      ------------------------------------------------------------

            c.8111
              .
PKD1P2      ------------------------------------------------------AGACCT
PKD1P5      ------------------------------------------------------AGACCT
```

```
PKD1          CGCGGGCACCGTGACGCCCACCGCCATCGGAGACAGCATCCTCAACATCACAGGAGACCT
PKD1P4        CGCGGGCACCGTGACGCCCACCGCCATCGGAGACAGCATCCTCAACATCACAGGAGACCT
PKD1P1        CGCGGGCACCGTGACGCCCACCGCCATCGGAGACAGCATCCTCAACATCACAGGAGACCT
PKD1P3        CGCGGGCACCGTGACGCCCACCGCCATCGGAGACAGCATCCTCAACATCACAGGAGACCT
PKD1P6        ------------------------------------------------------AGACCT
                                                                    ******

PKD1P2        CATCCACCTGGCCAGCTCAGACGTGCGGGCACCGCAGCGCTCAGAGCTGGGAGCCGAGTC
PKD1P5        CATCCACCTGGCCAGCTCAGACGTGCGGGCACCACAGCGCTCAGAGCTGGGAGCCGAGTC
PKD1          CATCCACCTGGCCAGCTCGGACGTGCGGGCACCACAGCCCTCAGAGCTGGGAGCCGAGTC
PKD1P4        CATCCACCTGGCCAGCTCAGACGTGCGGGCACCGCAGCGCTCAGAGCTGGGAGCCGAGTC
PKD1P1        CATCCACCTGGCCAGCTCAGACGTGCGGGCACCACAGCGCTCAGAGCTGGGAGCCGAGTC
PKD1P3        CATCCACCTGGCCAGCTCAGACGTGCGGGCACCACAGCGCTCAGAGCTGGGAGCCGAGTC
PKD1P6        CATCCACCTGGCCAGCTCAGACGTGCGGGCACCACAGCGCTCAGAGCTGGGAGCCGAGTC
              ****************** ************** **** *********************

PKD1P2        ACCATTGCGGATGGTGGCGTCCCAGGCCTACAACCTGACCTCTGCCCTCATGCGCATCCT
PKD1P5        ACCATCGCGGATGGTGGCGTCCCAGGCCTACAACCTGACCTCTGCCCTCACGCCCATCGT
PKD1          ACCATCTCGGATGGTGGCGTCCCAGGCCTACAACCTGACCTCTGCCCTCATGCGCATCCT
PKD1P4        ACCATTGCGGATGGTGGCGTCCCAGGCCTACAACCTGACCTCTGCCCTCATGCGCATCCT
PKD1P1        ACCATCGCGGATGGTGGCGTCCCAGGCCTACAACCTGACCTCTGCCCTCACGCCCATCGT
PKD1P3        ACCATCGCGGATGGTGGCGTCCCAGGCCTACAACCTGACCTCTGCCCTCACGCCCATCCT
PKD1P6        ACCATCGCGGATGGTGGCGTCCCAGGCCTACAACCTGACCTCTGCCCTCATGCGCATCCT
              *****  ******************************************* ** **** *

PKD1P2        CACGCGCTCCCGCGTGCTCAACGAGGAGCCCGTGACGCTGGCGGGCGAGGAGATCATGGC
PKD1P5        CACGCGCTCCCGCGTGCTCAACGAGGAGCCCCTGACGCTGGCGGGTGAGGAGATCGTGGC
PKD1          CATGCGCTCCCGCGTGCTCAACGAGGAGCCCCTGACGCTGGCGGGCGAGGAGATCGTGGC
PKD1P4        CACGCGCTCCCGCGTGCTCAACGAGGAGCCCGTGACGCTGGCGGGCGAGGAGATCATGGC
PKD1P1        CACGCGCTCCCGCGTGCTCAACGAGGAGCCCCTGACGCTGGCGGGTGAGGAGATCGTGGC
PKD1P3        CACGCGCTCCCGCGTGCTCAACGAGGAGCCCCTGACGCTGGCGGGTGAGGAGATCGTGGC
PKD1P6        CACGCGCTCCCGCGTGCTCAACGAGGAGCCCCTGACACTGGCGGGCGAGGAGATCGTGGC
              ** **************************** **** ******** ********* ****

PKD1P2        CCAGGGCAAGCGCTCGGACCCGCGGAGCCTGCTGTGCTATGGCGGCGCCCCAGGGCCTGG
PKD1P5        CCAGGGCAAGCGCTCGGACCCGCGGAGCCTGCTGTGCTATGGCGGCGCCCCAGGGCCTGG
PKD1          CCAGGGCAAGCGCTCGGACCCGCGGAGCCTGCTGTGCTATGGCGGCGCCCCAGGGCCTGG
PKD1P4        CCAGGGCAAGCGCTCGGACCCGCGGAGCCTGCTGTGCTATGGCGGCGCCCCAGGGCCTGG
PKD1P1        CCAGGGCAAGCGCTCGGACCCGCGGAGCCTGCTGTGCTATGGCGGCGCCCCAGGGCCTGG
PKD1P3        CCAGGGCAAGCGCTCGGACCCGCGGAGCCTGCTGTGCTATGGCGGCGCCCCAGGGCCTGG
PKD1P6        CCAGGGCAAGCGCTCGGACCCGCGGAGCCTGCTGTGCTATGGCGGCGCCCCAGGGCCTGG
              ************************************************************

PKD1P2        CTGCCACCTCTCCATCCCCTAGGCTTTCAGCAGGGCCCCGGCCAACCTCAGTGACGTGGT
PKD1P5        CTGCCACTTCTCCATCCCCTAGGCTTTCAGCAGGGCCCCGGCCAACCTCAGTGACGTGGT
PKD1          CTGCCACTTCTCCATCCCCGAGGCTTTCAGCGGGGCCCTGGCCAACCTCAGTGACGTGGT
PKD1P4        CTGCCACCTCTCCATCCCCTAGGCTTTCAGCAGGGCCCCCGCCAACCTCAGTGACGTGGT
PKD1P1        CTGCCACTTCTCCATCCCCTAGGCTTTCAGCAGGGCCCCGGCCAACCTCAGTGACGTGGT
PKD1P3        CTGCCACTTCTCCATCCCCTAGGCTTTCAGCAGGGCCCCGGCCAACCTCAGTGACGTGGT
PKD1P6        CTGCCACTTCTCCATCCCCTAGGCTTTCAGCAGGGCCCCGGCTAACCTCAGTGACGTGGT
              ******* *********** *********** ******  ** *****************

PKD1P2        GCAGCTCGTCTTTCTGGTGGACTCCAATCCCTTTCTCTTTGGCTATATCAGCAACTACAC
PKD1P5        GCAGCTCATCTTTCTGGTGGACTCCAATCCCTTTCCCTTTGGCTATATCAGCAACTACAC
PKD1          GCAGCTCATCTTTCTGGTGGACTCCAATCCCTTTCCCTTTGGCTATATCAGCAACTACAC
PKD1P4        GCAGCTCGTCTTTCTGGTGGACTCCAATCCCTTTCTCTTTGGCTATATCAGCAACTACAC
PKD1P1        GCAGCTCATCTTTCTGGTGGACTCCAATCCCTTTCCCTTTGGCTATATCAGCAACTACAC
PKD1P3        GCAGCTCATCTTTCTGGTGGACTCCAATCCCTTTCCCTTTGGCTATATCAGCAACTACAC
PKD1P6        GCAGCTCATCTTTCTGGTGGACTCCAATCCCTTTCCCTTTGGCTATATCAGCAACTACAC
              ******* *************************** ************************

PKD1P2        CGTCTCCACCAAGGTGGCCTCGATGGCGTTCCAGACACAGGCCGGCGCCCAGATCCCCAT
PKD1P5        CGTCTCCACCAAGGTGGCCTCGATGGCGTTCCAGACACAGGCCGGCGCCCAGATCCCCAT
PKD1          CGTCTCCACCAAGGTGGCCTCGATGGCATTCCAGACACAGGCCGGCGCCCAGATCCCCAT
PKD1P4        CGTCTCCACCAAGGTGGCCTCGATGGCGTTCCAGACACAGGCCGGCGCCCAGATCCCCAT
PKD1P1        CGTCTCCACCAAGGTGGCCTCGATGGCGTTCCAGACACAGGCCGGCGCCCAGATCCCCAT
PKD1P3        CGTCTCCACCAAGGTGGCCTCGATGGCGTTCCAGACACAGGCCGGCGCCCAGATCCCCAT
PKD1P6        CGTCTCCACCAAGGTGGCCTCGATGGCGTTCCAGACACAGGCCGGCGCCCAGATCCCCAT
              ************************** *********************************

PKD1P2        CGAGCGGCTGGCCTCAGAGCGCGCCATCACCGTGAAGGTGCCCAACAACTCGGACTGGGC
PKD1P5        CGAGCGGCTGGCCTCAGAGCGCGCC-TCACCGTGAAGGTGCCCAACAACTCGGACTGGGC
PKD1          CGAGCGGCTGGCCTCAGAGCGCGCCATCACCGTGAAGGTGCCCAACAACTCGGACTGGGC
PKD1P4        CGAGCGGCTGGCCTCAGAGCGCGCCATCACCGTGAAGGTGCCCAACAACTCGGACTGGGC
PKD1P1        CGAGCGGCTGGCCTCAGAGCGCGCC-TCACCGTGAAGGTGCCCAACAACTCGGACTGGGC
PKD1P3        CGAGCGGCTGGCCTCAGAGCGCGCC-TCACCGTGAAGGTGCCCAACAACTCGGACTGGGC
PKD1P6        CGAGCGGCTGGCCTCAGAGCGCGCCATCACCGTGAAGGTGCCCAACAACTCGGACTGGGC
              ************************* **********************************

PKD1P2        TGCCCGGGGCCACTGCAGCTCCGCCAACTCCG---------TTGTGGTCCAGCCCCAGGC
PKD1P5        TGCCCGGGGCCACCGCAGCTCCGCCAACTCCG---------TTGTGGTCCAGCCCCAGGC
PKD1          TGCCCGGGGCCACCGCAGCTCCGCCAACTCCGCCAACTCCGTTGTGGTCCAGCCCCAGGC
PKD1P4        TGCCCGGGGCCACCGCAGCTCCGCCAACTCCG---------TTGTGGTCCAGCCCCAGGC
PKD1P1        TGCCCGGGGCCACCGCAGCTCCGCCAACTCCG---------TTGTGGTCCAGCCCCAGGC
PKD1P3        TGCCCGGGGCCACCGCAGCTCCGCCAACTCCG---------TTGTGGTCCAGCCCCAGGC
PKD1P6        TGCCCGGGGCCACCGCAGCTCTGCCAACTCCG---------TTGTGGTCCAGCCCCAGGC
              ************* ******* **********         *******************

PKD1P2        CTCCGTCGGTGCTGTGGTCACCCTGGACAGCAGCAACCCTGTGGCCGTGCTGCATCTGCA
PKD1P5        CTCCGTCGGTGCTGTGGTCACCCTGGACAGCAGCAACCCTGCGGCCGTGCTGCATCTGCA
PKD1          CTCCGTCGGTGCTGTGGTCACCCTGGACAGCAGCAACCCTGCGGCCGGGCTGCATCTGCA
PKD1P4        CTCCGTCGGTGCTGTGGTCACCCTGGACAGCAGCAGCCCTGTGGCCGTGCTGCATCTGCA
PKD1P1        CTCCGTCGGTGCTGTGGTCACCCTGGACAGCAGCAACCCTGCGGCCGTGCTGCATCTGCA
PKD1P3        CTCCGTCGGTGCTGTGGTCACCCTGGACAGCAGCAACCCTGCGGCCGTGCTGCATCTGCA
PKD1P6        CTCCGTCGGTGCTGTGGTCACCCTGGACAGCAGCAACCCTGCGGCCGTGCTGCATCTGCA
              *********************************** ***** ***** ************

PKD1P2        GCTCAACTATACGCTGCTGGACGG------------------------------------
```

```
PKD1P5          GCTCAACTATACGCTGCTGGACGG------------------------------------
PKD1            GCTCAACTATACGCTGCTGGACGG------------------------------------
PKD1P4          GCTCAACTATACGCTGCTGGACGG------------------------------------
PKD1P1          GCTCAACTATACGCTGCTGGACGGTGCATGCAGCGGTTGGGGCACACGCGGCCCCCTGGC
PKD1P3          GCTCAACTATACGCTGCTGGACGGTGCATGCAGCGGTTGGGGCACACGCGGCCCCCTGGC
PKD1P6          GCTCAACTATACGCTGCTGGACGG------------------------------------
                ************************

PKD1P2          ------------------------------------------------------------
PKD1P5          ------------------------------------------------------------
PKD1            ------------------------------------------------------------
PKD1P4          ------------------------------------------------------------
PKD1P1          CTTGTTCTTGGGGGGAAGGCGTTTCTCGTAGGGCTTCCATGGGTGTCTCTGGTGAAATTT
PKD1P3          CTTGTTCTTGGGGGGAAGGCGTTTCTCGTAGGGCTTCCATGGGTGTCTCTGGTGAAATTT
PKD1P6          ------------------------------------------------------------


PKD1P2          ------------------------------------------------------------
PKD1P5          ------------------------------------------------------------
PKD1            ------------------------------------------------------------
PKD1P4          ------------------------------------------------------------
PKD1P1          GCTTTCTGTTTCATGGGCTGCTGGGGGCCTGGCCGGAGAGGAGCTGGGGGCCACGGAGAA
PKD1P3          GCTTTCTGTTTCATGGGCTGCTGGGGGCCTGGCCGGAGAGGAGCTGGGGGCCACGGAGAA
PKD1P6          ------------------------------------------------------------


PKD1P2          -----CCGCTACCTGTCTGAGGAACCCGAGCCCTACCTGGCAGTCTACCTGCACTCGGAG
PKD1P5          -----CCGCTACCTGTCTGAGGAACCCGAGCCCTACCTGGCAGTCTACCTGCACTCGGAG
PKD1            -----CCACTACCTGTCTGAGGAACCTGAGCCCTACCTGGCAGTCTACCTACACTCGGAG
PKD1P4          -----CCGCTACCTGTCTGAGGAACCCGAGCCCTACCTGGCAGTCTACCTGCACTCGGAG
PKD1P1          GCAGGCCGCTACCTGTCTGAGGAACCCGAGCCCTACCTGGCAGTCTACCTGCACTCGGAG
PKD1P3          GCAGGCCGCTACCTGTCTGAGGAACCCGAGCCCTACCTGGCAGTCTACCTGCACTCGGAG
PKD1P6          -----CCGCTACCTGTCTGAGGAACCCGAGCCCTACCTGGCAGTCTACCTGCACTCGGAG
                     ** ****************** *********************** *********

PKD1P2          CCCCGGCCCAATGAGCGCAACTGCTCGGCTAGCAGGAGGATCCGCCCAGAGTCCCTCCAG
PKD1P5          CCCCGGCCCAATGAGCGCAACTGCTCGGCTAGCAGGAGGATCCGCCCAGAGTCCCTCCAG
PKD1            CCCCGGCCCAATGAGCACAACTGCTCGGCTAGCAGGAGGATCCGCCCAGAGTCACTCCAG
PKD1P4          CCCCGGCCCAATGAGCGCAACTGCTCGGCTAGCAGGAGGATCCGCCCAGAGTCCCTCCAG
PKD1P1          CCCCGGCCCAATGAGCGCAACTGCTCGGCTAGCAGGAGGATCCGCCCAGAGTCCCTCCAG
PKD1P3          CCCCGGCCCAATGAGCGCAACTGCTCGGCTAGCAGGAGGATCCGCCCAGAGTCCCTCCAG
PKD1P6          CCCCGGCCCAATGAGCACAACTGCTCGGCTAGCAGGAGGATCCGCCCAGAGTCCCTCCAG
                **************** ************************************ ******

PKD1P2          GGTGCCGACCACCGGCCCTACACCTTCTTCATTTCCCCGGGGACCAGAGACCCAGTGGGG
PKD1P5          GGTGCCGACCACCGGCCCTACACCTTCTTCATTTCCCCGGGGACCAGAGACCCAGTGGGG
PKD1            GGTGCTGACCACCGGCCCTACACCTTCTTCATTTCCCCGGGGAGCAGAGACCCAGCGGGG
PKD1P4          GGTGCCGACCACCGGCCCTACACCTTCTTCATTTCCCCGGGGACCAGAGACCCAGTGGGG
PKD1P1          GGTGCCGACCACCGGCCCTACACCTTCTTCATTTCCCCGGGGACCAGAGACCCAGTGGGG
PKD1P3          GGTGCCGACCACCGGCCCTACACCTTCTTCATTTCCCCGGGGACCAGAGACCCAGTGGGG
PKD1P6          GGTGCCGACCACCGGCCCTACACCTTCTTCATTTCCCCGGGGACCAGAGACCCAGTGGGG
                ***** ************************************* *********** ****

PKD1P2          AGTTACCGTCTGAACCTCTCCAGCCACTTCCGCTGGTCGGCGCTGGAGGTGTCCGTGGGC
PKD1P5          AGTTACCGTCTGAACCTCTCCAGCCACTTCCGCTGGTCGGCGCTGGAGGTGTCCGTGGGC
PKD1            AGTTACCATCTGAACCTCTCCAGCCACTTCCGCTGGTCGGCGCTGCAGGTGTCCGTGGGC
PKD1P4          AGTTACCGTCTGAACCTCTCCAGCCACTTCCGCTGGTCGGCGCTGGAGGTGTCCGTGGGC
PKD1P1          AGTTACCGTCTGAACCTCTCCAGCCACTTCCGCTGGTCGGCGCTGGAGGTGTCCGTGGGC
PKD1P3          AGTTACCGTCTGAACCTCTCCAGTCACTTCCGCTGGTCGGCGCTGGAGGTGTCCGTGGGC
PKD1P6          AGTTACCGTCTGAACCTCTCCAGCCACTTCCGCCTGTCGGCGCTGGAGGTGTCCGTGGGC
                ******* *************** *********  ********** **************

PKD1P2          TTGTACACGTCCCTGTGCCAGTACTTCAGCGAGGAGGATGTGGTGTGGCGGACAGAGGGG
PKD1P5          TTGTACACGTCCCTGTGCCAGTACTTCAGCGAGGAGGACGTGGTGTGGCGGACAGAGGGG
PKD1            CTGTACACGTCCCTGTGCCAGTACTTCAGCGAGGAGGACATGGTGTGGCGGACAGAGGGG
PKD1P4          TTGTACACGTCCCTGTGCCAGTACTTCAGCGAGGAGGATGTGGTGTGGCGGACAGAGGGG
PKD1P1          TTGTACACGTCCCTGTGCCAGTACTTCAGCGAGGAGGACGTGGTGTGGCGGACAGAGGGG
PKD1P3          TTGTACACGTCCCTGTGCCAGTACTTCAGCGAGGAGGACGTGGTGTGGCGGACAGAGGGG
PKD1P6          CTGTACACGTCCCTGTGCCAGTACTTCAGCGAGGAGGACGTGGTGTGGCGGACAGAGGGG
                 *************************************  ********************

PKD1P2          CTGCTGCCCCTGGAGGAGACCTCGCCCCGCCAGGCCGTCTGCCTCACCCGCCACCTCACC
PKD1P5          CTGCTGCCCCTGGAGGAGACCTCGCCCCGCCAGGCCGTCTGCCTCACCCGCCACCTCACC
PKD1            CTGCTGCCCCTGGAGGAGACCTCGCCCCGCCAGGCCGTCTGCCTCACCCGCCACCTCACC
PKD1P4          CTGCTGCCCCTGGAGGAGACCTCGCCCCGCCAGGCCGTCTGCCTCACCCGCCACCTCACC
PKD1P1          CTGCTGCCCCTGGAGGAGACCTCGCCCCGCCAGGCCGTCTGCCTCACCCGCCACCTCACC
PKD1P3          CTGCTGCCCCTGGAGGAGACCTCGCCCCGCCAGGCCGTCTGCCTCACCCGCCACCTCACC
PKD1P6          CTGCTGCCCCTGGAGGAGACCTCGCCCCGCCAGGCCGTCTGCCTCACCCGCCACCTCACC
                ************************************************************

PKD1P2          GCCTTCGGCACCAGCCTCTTCATGCCCCCAAGCCATGTACGCTTTGTGTTTCCTGAGCCG
PKD1P5          GCCTTCGGCACCAGCCTCTTCGTGCCCCCAAGCCATATCCGCTTTGTGTTTCCTGAGCCA
PKD1            GCCTTCGGCGCCAGCCTCTTCGTGCCCCCAAGCCATGTCCGCTTTGTGTTTCCTGAGCCG
PKD1P4          GCCTTCGGCGCCAGCCTCTTCATGCCCCCAAGCCATGTACGCTTTGTGTTTCCTGAGCCG
PKD1P1          GCCTTCGGCACCAGCCTCTTCATGCCCCCAAGCCATGTACGCTTTGTGTTTCCTGAGCCA
PKD1P3          GCCTTCGGCACCAGCCTCTTCGTGCCCCCAAGCCATATCCGCTTTGTGTTTCCTGAGCCA
PKD1P6          GCCTTCGGCGCCAGCCTCTTCGTGCCCCCAAGCCATGTACGCTTTGTGTTTCCTGAGCCG
                ********* *********** ************** * ******************** 

PKD1P2          ACAGCGGATGTAAACTACATCGTCATGCTGACATGTGCTGTGTGCCTGGTGACCTACATG
PKD1P5          ACAGCGGATGTAAACTACATCGTCATGCTGACATGTGCTGTGTGCCTGGTGACCTACATG
PKD1            ACAGCGGATGTAAACTACATCGTCATGCTGACATGTGCTGTGTGCCTGGTGACCTACATG
PKD1P4          ACAGCGGATGTAAACTACATCGTCATGCTGACATGTGCTGTGTGCCTGGTGACCTACATG
PKD1P1          ACAGCGGATGTAAACTACATCGTCATGCTGACATGTGCTGTGTGCCTGGTGACCTACATG
PKD1P3          ACAGCGGATGTAAACTACATCGTCATGCTGACATGTGCTGTGTGCCTGGTGACCTACATG
PKD1P6          ACAGCGGATGTAAACTACATCGTCATGCTGACATGTGCTGTGTGCCTGGTGACCTACATG
                ************************************************************
```

```
PKD1P2              GTCATGGCCGCCATCCTGCACAAGCTGGACCAGTTGGATGCCAGCCGGGGCTGCGCCATC
PKD1P5              GTCATGGCCGCCATCCTGCACAAGCTGGACCAGTTGGATGCCAGCCGGGGCTGCGCCATC
PKD1                GTCATGGCCGCCATCCTGCACAAGCTGGACCAGTTGGATGCCAGCCGGGGCCGCGCCATC
PKD1P4              GTCATGGCCGCCATCCTGCACAAGCTGGACCAGTTGGATGCCAGCCGGGGCTGCGCCATC
PKD1P1              GTCATGGCCGCCATCCTGCACAAGCTGGACCAGTTGGATGCCAGCCGGGGCTGCGCCATC
PKD1P3              GTCATGGCCGCCATCCTGCACAAGCTGGACCAGTTGGATGCCAGCCGGGGCCGCGCCATC
PKD1P6              GTCATGGCCGCCATCCTGCACAAGCTGGACCAGTTGGATGCCAGCCGGGGCCGCGCCATC
                    *************************************************** ********

PKD1P2              CCCTTCTGTGGGCAGCGGGGCCGCTTCAAGTACGAGATCCTCGTCAAGACAGGCTGGGGC
PKD1P5              CCCTTCTGTGGGCAGCGGGGCCGCTTCAAGTACGAGATCCTCGTCAAGACAGGCTGGGGC
PKD1                CCTTTCTGTGGGCAGCGGGGCCGCTTCAAGTACGAGATCCTCGTCAAGACAGGCTGGGGC
PKD1P4              CCCTTCTGTGGGCAGCGGGGCCGCTTCAAGTACGAGATCCTCGTCAAGACAGGCTGGGGC
PKD1P1              CCCTTCTGTGGGCAGCGGGGCCGCTTCAAGTACGAGATCCTCGTCAAGACAGGCTGGGGC
PKD1P3              CCCTTCTGTGGGCAGCGGGGCCGCTTCAAGTACGAGATCCTCGTCAAGACAGGCTGGGGC
PKD1P6              CCCTTCTGTGGGCAGCGGGGCCGCTTCAAGTACGAGATCCTCGTCAAGACAGGCTGGGGC
                    ** *********************************************************

PKD1P2              CGGGGCTCAGGTACCACGGCCCACGTGGGCATCATGCTGTATGGGGTGGACAGCCGGAGT
PKD1P5              CGGGGCTCAGGTACCACGGCCCACGTGGGCATCATGCTGTATGGGGTGGACAGCCGGAGC
PKD1                CGGGGCTCAGGTACCACGGCCCACGTGGGCATCATGCTGTATGGGGTGGACAGCCGGAGC
PKD1P4              CGGGGCTCAGGTACCACGGCCCACGTGGGCATCATGCTGTATGGGGTGGACAGCCGGAGC
PKD1P1              CGGGGCTCAGGTACCACGGCCCACGTGGGCATCATGCTGTATGGGGTGGACAGCCGGAGC
PKD1P3              CGGGGCTCAGGTACCACGGCCCACGTGGGCATCATGCTGTATGGGGTGGACAGCCGGAGC
PKD1P6              CGGGGCTCAGGTACCACGGCCCACGTGGGCATCATGCTGTATGGGGTGGACAGCCGGAGC
                    ***********************************************************

                c.9448
                    .
PKD1P2              GGCCACCGGCACCTGGACGGCGACAGAGCCTTCCACCGCAACAGTCTGGACATCTTCCGG
PKD1P5              GGCCACCGGCACCTGGACGGCGACAGAGCCTTCCACCGCAACAGTCTGGACATCTTCCAG
PKD1                GGCCACCGGCACCTGGACGGCGACAGAGCCTTCCACCGCAACAGCCTGGACATCTTCCGG
PKD1P4              GGCCACCGGCACCTGGACGGCGACAGAGCCTTCCACCGCAACAGTCTGGACATCTTCCGG
PKD1P1              GGCCACCGGCACCTGGACGGCGACAGAGCCTTCCACCGCAACAGTCTGGACATCTTCCAG
PKD1P3              GGCCACCGGCACCTGGACGGCGACAGAGCCTTCCACCGCAACAGTCTGGACATCTTCCAG
PKD1P6              GGCCACCGGCACCTGGATGGCGACAGAGCCTTCCACCGCAACAGCCTGGACATCTTCCGG
                    ***************** ************************** ************* *

PKD1P2              ATCGCCACCCCGCACAGCCTGGGTAGCGTGTGGAAGATCCGAGTGTGGCACGACAACAAA
PKD1P5              ATCGCCACCCCGCACAGCCTGGGTAGCATGTGGAAGATCCGAGTGTGGCACGACAACAAA
PKD1                ATCGCCACCCCGCACAGCCTGGGTAGCGTGTGGAAGATCCGAGTGTGGCACGACAACAAA
PKD1P4              ATCGCCACCCCGCACAGCCTGGGTAGCGTGTGGAAGATCCGAGTGTGGCACGACAACAAA
PKD1P1              ATCGCCACCCCGCACAGCCTGGGTAGCATGTGGAAGATCCGAGTGTGGCACGACAACAAA
PKD1P3              ATCGCCACCCCGCACAGCCTGGGTAGCGTGTGGAAGATCCGAGTGTGGCACGACAACAAA
PKD1P6              ATCGCCACCCCGCACAGCCTGGGTAGCGTGTGGAAGATCCGAGTGTGGCACGACA---AA
                    *************************** ***************************   **

PKD1P2              GGGCTCAGCCCTGCCTGGTTCCTGCAGCACATCATCGTCAGGGACCTGCAGACGGCACGC
PKD1P5              GGGCTCAGCCCTGCCTGGTTCCTGCAGCACATCATCGTCAGGGACCTGCAGACGGCACGC
PKD1                GGGCTCAGCCCTGCCTGGTTCCTGCAGCACGTCATCGTCAGGGACCTGCAGACGGCACGC
PKD1P4              GGGCTCAGCCCTGCCTGGTTCCTGCAGCACATCATCGTCAGGGACCTGCAGACGGCACGC
PKD1P1              GGGCTCAGCCCTGCCTGGTTCCTGCAGCACATCATCGTCAGGGACCTGCAGACGGCACGC
PKD1P3              GGGCTCAGCCCTGCCCGGTTCCTGCAGCACATCATCGTCAGGGACCTGCAGACGGCACGC
PKD1P6              GGGCTCAGCCCTGCCTGGTTCCTGCAGCACGTCATCATCAGGGACCTGCAGACGGCACAC
                    *************** ************** ***** ********************* *

PKD1P2              AGCACCTTCTTCCTGGTCAATGACTGGCTTTCGGTGGAGACGGAGGCCAACGGGGGCCTG
PKD1P5              AGCACCTTCTTCCTGGTCAATGACTGGCTTTCGGTGGAGACGGAGGCCAACGGGGGCCTG
PKD1                AGCGCCTTCTTCCTGGTCAATGACTGGCTTTCGGTGGAGACGGAGGCCAACGGGGGCCTG
PKD1P4              AGCACCTTCTTCCTGGTCAATGACTGGCTTTCGGTGGAGACGGAGGCCAACGGGGGCCTG
PKD1P1              AGCACCTTCTTCCTGGTCAATGACTGGCTTTCGGTGGAGACGGAGGCCAACGGGGGCCTG
PKD1P3              AGCACCTTCTTCCTGGTCAATGACTGGCTTTCGGTGGAGACGGAGGCCAACGGGGGCCTG
PKD1P6              AGCACCTTCTTCCTGGTCAATGACTGGCTTTCGGTGGAGACGGAGGCCAACGGGGGCCTG
                    *** ********************************************************

PKD1P2              GTGGAGAAGGAGGTGCTGGCTGCGAGTCACGCAGCCCTGTTGCGCTTCCGGCGCCTGCTG
PKD1P5              GTGGAGAAGGAGGTGCTGGCCGCGAGTCACGCAGCCCTGTTGCGCTTCCGGCGCCTGCTG
PKD1                GTGGAGAAGGAGGTGCTGGCCGCGAGCGACGCAGCCCTTTTGCGCTTCCGGCGCCTGCTG
PKD1P4              GTGGAGAAGGAGGTGCTGGCTGCGAGTCACGCAGCCCTGTTGCGCTTCCGGCGCCTGCTG
PKD1P1              GTGGAGAAGGAGGTGCTGGCCGCGAGTCACGCAGCCCTGTTGCGCTTCCGGCGCCTGCTG
PKD1P3              GTGGAGAAGGAGGTGCTGGCCGCGAGTCACGCAGCCCTGTTGCGCTTCCGGCGCCTGCTG
PKD1P6              GTGGAGAAGGAGGTGCTGGCCGCGAGTCACGCAGCCCTGTTGCGCTTCCGGCGCCTGCTG
                    ******************** *****  ********** *********************

PKD1P2              GTGGCTGAGCTGCAGCGTGGCTTCTTTGACAAGCACATCTGGCTCTCCATATGGGACCGG
PKD1P5              GTGGCTGAGCTGCAGCGTGGCTTCTTTGACAAGCACATCTGGCTCTCCATATGGGACCGG
PKD1                GTGGCTGAGCTGCAGCGTGGCTTCTTTGACAAGCACATCTGGCTCTCCATATGGGACCGG
PKD1P4              GTGGCTGAGCTGCAGCGTGGCTTCTTTGACAAGCACATCTGGCTCTCCATATGGGACCGG
PKD1P1              GTGGCTGAGCTGCAGCGTGGCTTCTTTGACAAGCACATCTGGCTCTCCATATGGGACCGG
PKD1P3              GTGGCTGAGCTGCAGCGTGGCTTCTTTGACAAGCACATCTGGCTCTCCATATGGGACCGG
PKD1P6              GTGGCTGAGCTGCAGTGCGGCTTCTTTGACAAGCACATCTGGCTCTCCATATGGGACCGG
                    *************** * ******************************************

PKD1P2              CCACCTCGTAGCTGTTTCACTCGCATCCAGAGGGCCACCTGCTGCGTTCTCCTCATCTGC
PKD1P5              CCGCCTCGTAGCTGTTTCACTCGCATCCAGAGGGCCACCTGCTGCGATCTCCTCATCTGT
PKD1                CCGCCTCGTAGCCGTTTCACTCGCATCCAGAGGGCCACCTGCTGCGTTCTCCTCATCTGC
PKD1P4              CCACCTCGTAGCTGTTTCACTCGCATCCAGAGGGCCACCTGCTGCGTTCTCCTCATCTGC
PKD1P1              CCGCCTCGGAGCTGTTTCACTCGCATCCAGAGGGCCACCTGCTGCGTTCTCCTCATCTGT
PKD1P3              CCGCCTCGTAGCTATTTCACTCACATCCAGAGGGCCACCTGCTGCGTTCTCCTCATCTGT
PKD1P6              CCGCCTCGTAGCTGTTTCACTCGCATCCAGAGGGCCACCTGCTGCGTTCTCCTCATCTGC
                    ** ***** ***  ******** *********************** ************

                                c.9889
                                       .
PKD1P2              CTCTTCCTGGGCGCCAACGCCGTGTGGTACGGGGCTGTTGGTGACTCTGCCTACAGCACG
PKD1P5              CTCTTCCTGGGCGCCAACGCCGTGTGGTACGGGGCTGTTGGAGACTCTGCCTACAGCACG
PKD1                CTCTTCCTGGGCGCCAACGCCGTGTGGTACGGGGCTGTTGGCGACTCTGCCTACAGCACG
PKD1P4              CTCTTCCTGGGCGCCAACGCCGTGTGGTACGGGGCTGTTGGTGACTCTGCCTACAGCACG
```

```
PKD1P1              CTCTTCCTGGGCGCCAACGCCGTGTGGTACGGGGCTGTTGGAGACTCTGCCTACAGCACG
PKD1P3              CTCTTCCTGGGCGCCAACGCCGTGTGGTACGGGGCTGTTGGAGACTCTGCCTACAGCACG
PKD1P6              CTCTTCCTGGGCGCCAACGCCGTGTGGTACGGGGCTGTTGGTGACTCTGCCTACAGCACG
                    ***************************************** ******************

PKD1P2              GGGCATGTGTCCAGGCTGAGCCCGCTGAGCGTCGACACAGTCGCTGTTGGCCTGGTGTCC
PKD1P5              GGGCGTGTGTCCAGGCTGAACCCGCTGAGCGTCGACACAGTCGCTGTTGGCCTGGTGTCC
PKD1                GGGCATGTGTCCAGGCTGAGCCCGCTGAGCGTCGACACAGTCGCTGTTGGCCTGGTGTCC
PKD1P4              GGGCATGTGTCCAGGCTGAGCCCGCTGAGCGTCGACACAGTCGCTGTTGGCCTGGTGTCC
PKD1P1              GGGCGTGTGTCCAGGCTGAACCCGCTGAGCGTCGACACAGTCGCTGTTGGCCTGGTGTCC
PKD1P3              GGGCGTGTGTCCAGGCTGAACCCGCTGAGCGTCGACACAGTCGCTGTTGGCCTGGTGTCC
PKD1P6              GGGCATGTGTCCAGGCTGAGCCCGCTGAGCGTCGACACAGTCGCTGTTGGCCTGGTGTCC
                    **** ************** ****************************************

PKD1P2              AGCGTGGTTGTCTATCCCGTCTACCTGGCCATCCTCTTTCTCTTCCGGATGTCCCGGAGC
PKD1P5              AGCGTGGTTGTCTATCCCGTCTACCTGGCCATCCTCTTTCTCTTCCGGATGTCCCGGAGC
PKD1                AGCGTGGTTGTCTATCCCGTCTACCTGGCCATCCTTTTTCTCTTCCGGATGTCCCGGAGC
PKD1P4              AGCGTGGTTGTCTATCCCGTCTACCTGGCCATCCTCTTTCTCTTCCGGATGTCCCGGAGC
PKD1P1              AGCGTGGTTGTCTATCCCGTCTACCTGGCCATCCTCTTTCTCTTCCGGATGTCCCGGAGC
PKD1P3              AGCGTGGTTGTCTATCCCGTCTACCTGGCCATCCTCTTTCTCTTCCGGATGTCCCGGAGC
PKD1P6              AGCGTGGTTGTCTATCCCGTCTACCTGGCCATCCTCTTTCTCTTCTGGATGTCCCGGAGC
                    *********************************** ********* **************

PKD1P2              AAGGTGGCTGGGAGCCTGAGCCCCACACCTGCCGGGCAGTAGGTGCTGGACATCGACAGC
PKD1P5              AAGGTGGCTGGGAGCCCGAGCCCCACACCTGCCGGGCAGCAGGTGCTGGACGTCGACAGC
PKD1                AAGGTGGCTGGGAGCCCGAGCCCCACACCTGCCGGGCAGCAGGTGCTGGACATCGACAGC
PKD1P4              AAGGTGGCTGGGAGCCTGAGCCCCACACCTGCCGGGCAGTAGGTGCTGGACATCGACAGC
PKD1P1              AAGGTGGCTGGGAGCCCGAGCCCCACACCTGCCGGGCAGCAGGTGCTGGACGTCGACAGC
PKD1P3              AAGGTGGCTGGGAGCCCGAGCCCCACACCTGCCGGGCAGCAGGTGCTGGACGTCGACAGC
PKD1P6              AAGGTGGCTGGGAGCCCGAGCCCCACACCTGCCGGGCAGCAGGTGCTGGACATCGACAGC
                    **************** ********************** *********** ********

PKD1P2              TGCCTGGACTCGTCCGTGCTGGACAGCTCCTTCCTCACGTTCTCAGGCCTCCACGCTGAG
PKD1P5              TGCCTGGACTCATCCGTGCTGGACAGCTCCTTCCTCACGTTCTCAGGCCTCCACGCTGAG
PKD1                TGCCTGGACTCGTCCGTGCTGGACAGCTCCTTCCTCACGTTCTCAGGCCTCCACGCTGAG
PKD1P4              TGCCTGGACTCGTCCGTGCTGGACAGCTCCTTCCTCACGTTCTCAGGCCTCCACGCTGAG
PKD1P1              TGCCTGGACTCATCCGTGCTGGACAGCTCCTTCCTCACGTTCTCAGGCCTCCACGCTGAG
PKD1P3              TGCCTGGACTCATCCGTGCTGGACAGCTCCTTCCTCACGTTCTGAGGCCTCCACGCTGAG
PKD1P6              TGCCTGGACTCGTCCGTGCTGGACAGCTCCTTCCTCACGTTCTCAGGCCTCCACGCTGAG
                    *********** ******************************* ****************

PKD1P2              ---------------------GCCTTTGCTGGACAGGTGAAGAGTGACTTGTTTCTGGAT
PKD1P5              C------------------AGGCCTTTGCTGGACAGATGAAGAGTGACTTGTTTCTGGAT
PKD1                C------------------AGGCCTTTGTTGGACAGATGAAGAGTGACTTGTTTCTGGAT
PKD1P4              ---------------------GCCTTTGCTGGACAGGTGAAGAGTGACTTGTTTCTGGAT
PKD1P1              ACCCTGTGCACCTCTCAGCAGGCCTTTGCTGGACAGATGAAGAGTGACTTGTTTCTGGAT
PKD1P3              C------------------AGGCCTTTGCTGGACAGATGAAGAGTGACTTGTTTCTGGAT
PKD1P6              ---------------------GCCTTTGCTGGACAGGTGAAGAGTGACTTGTTTCTGGAT
                                         ******* ******* ***********************
```

```
                                         c.10231
                                            .
PKD1P2              GATTCTAAGAGTCTGGTGTGCTGGCCCTCCAGCGAGGGAACGCCCAGTTGGCCGGACCTG
PKD1P5              GATTCTAAGAGTCTGGTGTGCTGGCCCTCCAGCGAGGGAACGCCCAGTTGGCCGGACCTG
PKD1                GATTCTAAGAGTCTGGTGTGCTGGCCCTCCGGCGAGGGAACGCTCAGTTGGCCGGACCTG
PKD1P4              GATTCTAAGAGTCTGGTGTGCTGGCCCTCCAGCGAGGGAACGCCCAGTTGGCCGGACCTG
PKD1P1              GATTCTAAGAGTCTGGTGTGCTGGCCCTCCAGCGAGGGAACGCCCAGTTGGCCGGACCTG
PKD1P3              GATTCTAAGAGTCTGGTGTGCTGGCCCTCCAGCGAGGGAACGCCCAGTTAGCCGGACCTG
PKD1P6              GATTCTAAGAGTCTGGTGTGCTGGCCCTCCAGCGAGGGAACGCCCAGTTGGCCGGACCTG
                    ****************************** ************* ***** **********

PKD1P2              CTCAGTGACCCGTCCATTGTGGGTAGCAATCTGCGGCA----------------------
PKD1P5              CTCAGTGACCCGTCCATTGTGGGTAGCAATCTGCGGCA----------------------
PKD1                CTCAGTGACCCGTCCATTGTGGGTAGCAATCTGCGGCAGCTGGCACGGGGCCAGGCGGGC
PKD1P4              CTCAGTGACCCGTCCATTGTGGGTAGCAATCTGCGGCA----------------------
PKD1P1              CTCAGTGACCCGTCCATTGTGGGTAGCAATCTG---------------------------
PKD1P3              CTCAGTGACCCGTCCATTGTGGGTAGCAATCTGCGGCA----------------------
PKD1P6              CTCAGTGACCCGTCCATTGTGGGTAGCAATCTGCG-------------------------
                    *********************************

PKD1P2              ------------------------------------------------------------
PKD1P5              ------------------------------------------------------------
PKD1                CATGGGCTGGGCCCAGAGGAGGACGGCTTCTCCCTGGCCAGCCCCTACTCGCCTGCCAAA
PKD1P4              ------------------------------------------------------------
PKD1P1              ------------------------------------------------------------
PKD1P3              ------------------------------------------------------------
PKD1P6              ------------------------------------------------------------

PKD1P2              ------------------------------------------------------------
PKD1P5              ------------------------------------------------------------
PKD1                TCCTTCTCAGCATCAGATGAAGACCTGATCCAGCAGGTCCTTGCCGAGGGGGTCAGCAGC
PKD1P4              ------------------------------------------------------------
PKD1P1              ------------------------------------------------------------
PKD1P3              ------------------------------------------------------------
PKD1P6              ------------------------------------------------------------

PKD1P2              ------------------------------------------------------------
PKD1P5              ------------------------------------------------------------
PKD1                CCAGCCCCTACCCAAGACACCCACATGGAAACGGACCTGCTCAGCAGCCTGTCCAGCACT
PKD1P4              ------------------------------------------------------------
PKD1P1              ------------------------------------------------------------
```

```
PKD1P3          ------------------------------------------------------------
PKD1P6          ------------------------------------------------------------

PKD1P2          ------------------------------------------------------------
PKD1P5          ------------------------------------------------------------
PKD1            CCTGGGGAGAAGACAGAGACGCTGGCGCTGCAGAGGCTGGGGGAGCTGGGGCCACCCAGC
PKD1P4          ------------------------------------------------------------
PKD1P1          ------------------------------------------------------------
PKD1P3          ------------------------------------------------------------
PKD1P6          ------------------------------------------------------------

PKD1P2          ------------------------------------------------------------
PKD1P5          ------------------------------------------------------------
PKD1            CCAGGCCTGAACTGGGAACAGCCCCAGGCAGCGAGGCTGTCCAGGACAGGACTGGTGGAG
PKD1P4          ------------------------------------------------------------
PKD1P1          ------------------------------------------------------------
PKD1P3          ------------------------------------------------------------
PKD1P6          ------------------------------------------------------------

PKD1P2          ------------------------------------------------------------
PKD1P5          ------------------------------------------------------------
PKD1            GGTCTGCGGAAGCGCCTGCTGCCGGCCTGGTGTGCCTCCCTGGCCCACGGGCTCAGCCTG
PKD1P4          ------------------------------------------------------------
PKD1P1          ------------------------------------------------------------
PKD1P3          ------------------------------------------------------------
PKD1P6          ------------------------------------------------------------

PKD1P2          ------------------------------------------------------------
PKD1P5          ------------------------------------------------------------
PKD1            CTCCTGGTGGCTGTGGCTGTGGCTGTCTCAGGGTGGGTGGGTGCGAGCTTCCCCCCGGGC
PKD1P4          ------------------------------------------------------------
PKD1P1          ------------------------------------------------------------
PKD1P3          ------------------------------------------------------------
PKD1P6          ------------------------------------------------------------

PKD1P2          ------------------------------------------------------------
PKD1P5          ------------------------------------------------------------
PKD1            GTGAGTGTTGCGTGGCTCCTGTCCAGCAGCGCCAGCTTCCTGGCCTCATTCCTCGGCTGG
PKD1P4          ------------------------------------------------------------
PKD1P1          ------------------------------------------------------------
PKD1P3          ------------------------------------------------------------
PKD1P6          ------------------------------------------------------------

PKD1P2          ------------------------------------------------------------
PKD1P5          ------------------------------------------------------------
PKD1            GAGCCACTGAAGGTCTTGCTGGAAGCCCTGTACTTCTCACTGGTGGCCAAGCGGCTGCAC
PKD1P4          ------------------------------------------------------------
PKD1P1          ------------------------------------------------------------
PKD1P3          ------------------------------------------------------------
PKD1P6          ------------------------------------------------------------

PKD1P2          ------------------------------------------------------------
PKD1P5          ------------------------------------------------------------
PKD1            CCGGATGAAGATGACACCCTGGTAGAGAGCCCGGCTGTGACGCCTGTGAGCGCACGTGTG
PKD1P4          ------------------------------------------------------------
PKD1P1          ------------------------------------------------------------
PKD1P3          ------------------------------------------------------------
PKD1P6          ------------------------------------------------------------

PKD1P2          ------------------------------------------------------------
PKD1P5          ------------------------------------------------------------
PKD1            CCCCGCGTACGGCCACCCCACGGCTTTGCACTCTTCCTGGCCAAGGAAGAAGCCCGCAAG
PKD1P4          ------------------------------------------------------------
PKD1P1          ------------------------------------------------------------
PKD1P3          ------------------------------------------------------------
PKD1P6          ------------------------------------------------------------

PKD1P2          ------------------------------------------------------------
PKD1P5          ------------------------------------------------------------
PKD1            GTCAAGAGGCTACATGGCATGCTGCGGAGCCTCCTGGTGTACATGCTTTTTCTGCTGGTG
PKD1P4          ------------------------------------------------------------
PKD1P1          ------------------------------------------------------------
PKD1P3          ------------------------------------------------------------
PKD1P6          ------------------------------------------------------------

PKD1P2          ------------------------------------------------------------
PKD1P5          ------------------------------------------------------------
PKD1            ACCCTGCTGGCCAGCTATGGGGATGCCTCATGCCATGGGCACGCCTACCGTCTGCAAAGC
PKD1P4          ------------------------------------------------------------
PKD1P1          ------------------------------------------------------------
PKD1P3          ------------------------------------------------------------
PKD1P6          ------------------------------------------------------------

PKD1P2          ------------------------------------------------------------
PKD1P5          ------------------------------------------------------------
PKD1            GCCATCAAGCAGGAGCTGCACAGCCGGGCCTTCCTGGCCATCACGCGGTCTGAGGAGCTC
PKD1P4          ------------------------------------------------------------
```

```
PKD1P1          ------------------------------------------------------------
PKD1P3          ------------------------------------------------------------
PKD1P6          ------------------------------------------------------------

PKD1P2          ------------------------------------------------------------
PKD1P5          ------------------------------------------------------------
PKD1            TGGCCATGGATGGCCCACGTGCTGCTGCCCTACGTCCACGGGAACCAGTCCAGCCCAGAG
PKD1P4          ------------------------------------------------------------
PKD1P1          ------------------------------------------------------------
PKD1P3          ------------------------------------------------------------
PKD1P6          ------------------------------------------------------------

PKD1P2          ------------------------------------------------------------
PKD1P5          ------------------------------------------------------------
PKD1            CTGGGGCCCCCACGGCTGCGGCAGGTGCGGCTGCAGGAAGCACTCTACCCAGACCCTCCC
PKD1P4          ------------------------------------------------------------
PKD1P1          ------------------------------------------------------------
PKD1P3          ------------------------------------------------------------
PKD1P6          ------------------------------------------------------------

PKD1P2          ------------------------------------------------------------
PKD1P5          ------------------------------------------------------------
PKD1            GGCCCCAGGGTCCACACGTGCTCGGCCGCAGGAGGCTTCAGCACCAGCGATTACGACGTT
PKD1P4          ------------------------------------------------------------
PKD1P1          ------------------------------------------------------------
PKD1P3          ------------------------------------------------------------
PKD1P6          ------------------------------------------------------------

PKD1P2          ------------------------------------------------------------
PKD1P5          ------------------------------------------------------------
PKD1            GGCTGGGAGAGTCCTCACAATGGCTCGGGGACGTGGGCCTATTCAGCGCCGGATCTGCTG
PKD1P4          ------------------------------------------------------------
PKD1P1          ------------------------------------------------------------
PKD1P3          ------------------------------------------------------------
PKD1P6          ------------------------------------------------------------

PKD1P2          ------------------------------------------------------------
PKD1P5          ------------------------------------------------------------
PKD1            GGGGCATGGTCCTGGGGCTCCTGTGCCGTGTATGACAGCGGGGGCTACGTGCAGGAGCTG
PKD1P4          ------------------------------------------------------------
PKD1P1          ------------------------------------------------------------
PKD1P3          ------------------------------------------------------------
PKD1P6          ------------------------------------------------------------

PKD1P2          ------------------------------------------------------------
PKD1P5          ------------------------------------------------------------
PKD1            GGCCTGAGCCTGGAGGAGAGCCGCGACCGGCTGCGCTTCCTGCAGCTGCACAACTGGCTG
PKD1P4          ------------------------------------------------------------
PKD1P1          ------------------------------------------------------------
PKD1P3          ------------------------------------------------------------
PKD1P6          ------------------------------------------------------------

PKD1P2          ------------------------------------------------------------
PKD1P5          ------------------------------------------------------------
PKD1            GACAACAGGAGCCGCGCTGTGTTCCTGGAGCTCACGCGCTACAGCCCGGCCGTGGGGCTG
PKD1P4          ------------------------------------------------------------
PKD1P1          ------------------------------------------------------------
PKD1P3          ------------------------------------------------------------
PKD1P6          ------------------------------------------------------------

PKD1P2          ------------------------------------------------------------
PKD1P5          ------------------------------------------------------------
PKD1            CACGCCGCCGTCACGCTGCGCCTCGAGTTCCCGGCGGCCGGCCGCGCCCTGGCCGCCCTC
PKD1P4          ------------------------------------------------------------
PKD1P1          ------------------------------------------------------------
PKD1P3          ------------------------------------------------------------
PKD1P6          ------------------------------------------------------------

PKD1P2          ------------------------------------------------------------
PKD1P5          ------------------------------------------------------------
PKD1            AGCGTCCGCCCCTTTGCGCTGCGCCGCCTCAGCGCGGGCCTCTCGCTGCCTCTGCTCACC
PKD1P4          ------------------------------------------------------------
PKD1P1          ------------------------------------------------------------
PKD1P3          ------------------------------------------------------------
PKD1P6          ------------------------------------------------------------

PKD1P2          ------------------------------------------------------------
PKD1P5          ------------------------------------------------------------
PKD1            TCGGTGTGCCTGCTGCTGTTCGCCGTGCACTTCGCCGTGGCCGAGGCCCGTACTTGGCAC
PKD1P4          ------------------------------------------------------------
PKD1P1          ------------------------------------------------------------
PKD1P3          ------------------------------------------------------------
PKD1P6          ------------------------------------------------------------

PKD1P2          ------------------------------------------------------------
PKD1P5          ------------------------------------------------------------
PKD1            AGGGAAGGGCGCTGGCGCGTGCTGCGGCTCGGAGCCTGGGCGCGGTGGCTGCTGGTGGCG
```

```
PKD1P4          ------------------------------------------------------------
PKD1P1          ------------------------------------------------------------
PKD1P3          ------------------------------------------------------------
PKD1P6          ------------------------------------------------------------

PKD1P2          ------------------------------------------------------------
PKD1P5          ------------------------------------------------------------
PKD1            CTGACGGCGGCCACGGCACTGGTACGCCTCGCCCAGCTGGGTGCCGCTGACCGCCAGTGG
PKD1P4          ------------------------------------------------------------
PKD1P1          ------------------------------------------------------------
PKD1P3          ------------------------------------------------------------
PKD1P6          ------------------------------------------------------------

PKD1P2          ------------------------------------------------------------
PKD1P5          ------------------------------------------------------------
PKD1            ACCCGTTTCGTGCGCGGCCGCCCGCGCCGCTTCACTAGCTTCGACCAGGTGGCGCAGCTG
PKD1P4          ------------------------------------------------------------
PKD1P1          ------------------------------------------------------------
PKD1P3          ------------------------------------------------------------
PKD1P6          ------------------------------------------------------------

PKD1P2          ------------------------------------------------------------
PKD1P5          ------------------------------------------------------------
PKD1            AGCTCCGCAGCCCGTGGCCTGGCGGCCTCGCTGCTCTTCCTGCTTTTGGTCAAGGCTGCC
PKD1P4          ------------------------------------------------------------
PKD1P1          ------------------------------------------------------------
PKD1P3          ------------------------------------------------------------
PKD1P6          ------------------------------------------------------------

PKD1P2          ------------------------------------------------------------
PKD1P5          ------------------------------------------------------------
PKD1            CAGCAGCTACGCTTCGTGCGCCAGTGGTCCGTCTTTGGCAAGACATTATGCCGAGCTCTG
PKD1P4          ------------------------------------------------------------
PKD1P1          ------------------------------------------------------------
PKD1P3          ------------------------------------------------------------
PKD1P6          ------------------------------------------------------------

PKD1P2          ------------------------------------------------------------
PKD1P5          ------------------------------------------------------------
PKD1            CCAGAGCTCCTGGGGGTCACCTTGGGCCTGGTGGTGCTCGGGGTAGCCTACGCCCAGCTG
PKD1P4          ------------------------------------------------------------
PKD1P1          ------------------------------------------------------------
PKD1P3          ------------------------------------------------------------
PKD1P6          ------------------------------------------------------------

PKD1P2          ------------------------------------------------------------
PKD1P5          ------------------------------------------------------------
PKD1            GCCATCCTGCTCGTGTCTTCCTGTGTGGACTCCCTCTGGAGCGTGGCCCAGGCCCTGTTG
PKD1P4          ------------------------------------------------------------
PKD1P1          ------------------------------------------------------------
PKD1P3          ------------------------------------------------------------
PKD1P6          ------------------------------------------------------------

PKD1P2          ------------------------------------------------------------
PKD1P5          ------------------------------------------------------------
PKD1            GTGCTGTGCCCTGGGACTGGGCTCTCTACCCTGTGTCCTGCCGAGTCCTGGCACCTGTCA
PKD1P4          ------------------------------------------------------------
PKD1P1          ------------------------------------------------------------
PKD1P3          ------------------------------------------------------------
PKD1P6          ------------------------------------------------------------

PKD1P2          ------------------------------------------------------------
PKD1P5          ------------------------------------------------------------
PKD1            CCCCTGCTGTGTGTGGGGCTCTGGGCACTGCGGCTGTGGGGCGCCCTACGGCTGGGGGCT
PKD1P4          ------------------------------------------------------------
PKD1P1          ------------------------------------------------------------
PKD1P3          ------------------------------------------------------------
PKD1P6          ------------------------------------------------------------

PKD1P2          ------------------------------------------------------------
PKD1P5          ------------------------------------------------------------
PKD1            GTTATTCTCCGCTGGCGCTACCACGCCTTGCGTGGAGAGCTGTACCGGCCGGCCTGGGAG
PKD1P4          ------------------------------------------------------------
PKD1P1          ------------------------------------------------------------
PKD1P3          ------------------------------------------------------------
PKD1P6          ------------------------------------------------------------

PKD1P2          ------------------------------------------------------------
PKD1P5          ------------------------------------------------------------
PKD1            CCCCAGGACTACGAGATGGTGGAGTTGTTCCTGCGCAGGCTGCGCCTCTGGATGGGCCTC
PKD1P4          ------------------------------------------------------------
PKD1P1          ------------------------------------------------------------
PKD1P3          ------------------------------------------------------------
PKD1P6          ------------------------------------------------------------

PKD1P2          ------------------------------------------------------------
PKD1P5          ------------------------------------------------------------
```

```
PKD1            AGCAAGGTCAAGGAGTTCCGCCACAAAGTCCGCTTTGAAGGGATGGAGCCGCTGCCCTCT
PKD1P4          ------------------------------------------------------------
PKD1P1          ------------------------------------------------------------
PKD1P3          ------------------------------------------------------------
PKD1P6          ------------------------------------------------------------

PKD1P2          ------------------------------------------------------------
PKD1P5          ------------------------------------------------------------
PKD1            CGCTCCTCCAGGGGCTCCAAGGTATCCCCGGATGTGCCCCCACCCAGCGCTGGCTCCGAT
PKD1P4          ------------------------------------------------------------
PKD1P1          ------------------------------------------------------------
PKD1P3          ------------------------------------------------------------
PKD1P6          ------------------------------------------------------------

PKD1P2          ------------------------------------------------------------
PKD1P5          ------------------------------------------------------------
PKD1            GCCTCGCACCCCTCCACCTCCTCCAGCCAGCTGGATGGGCTGAGCGTGAGCCTGGGCCGG
PKD1P4          ------------------------------------------------------------
PKD1P1          ------------------------------------------------------------
PKD1P3          ------------------------------------------------------------
PKD1P6          ------------------------------------------------------------

PKD1P2          ------------------------------------------------------------
PKD1P5          ------------------------------------------------------------
PKD1            CTGGGGACAAGGTGTGAGCCTGAGCCCTCCCGCCTCCAAGCCGTGTTCGAGGCCCTGCTC
PKD1P4          ------------------------------------------------------------
PKD1P1          ------------------------------------------------------------
PKD1P3          ------------------------------------------------------------
PKD1P6          ------------------------------------------------------------

PKD1P2          ------------------------------------------------------------
PKD1P5          ------------------------------------------------------------
PKD1            ACCCAGTTTGACCGACTCAACCAGGCCACAGAGGACGTCTACCAGCTGGAGCAGCAGCTG
PKD1P4          ------------------------------------------------------------
PKD1P1          ------------------------------------------------------------
PKD1P3          ------------------------------------------------------------
PKD1P6          ------------------------------------------------------------

PKD1P2          ------------------------------------------------------------
PKD1P5          ------------------------------------------------------------
PKD1            CACAGCCTGCAAGGCCGCAGGAGCAGCCGGGCGCCCGCCGGATCTTCCCGTGGCCCATCC
PKD1P4          ------------------------------------------------------------
PKD1P1          ------------------------------------------------------------
PKD1P3          ------------------------------------------------------------
PKD1P6          ------------------------------------------------------------

PKD1P2          ------------------------------------------------------------
PKD1P5          ------------------------------------------------------------
PKD1            CCGGGCCTGCGGCCAGCACTGCCCAGCCGCCTTGCCCGGGCCAGTCGGGGTGTGGACCTG
PKD1P4          ------------------------------------------------------------
PKD1P1          ------------------------------------------------------------
PKD1P3          ------------------------------------------------------------
PKD1P6          ------------------------------------------------------------

PKD1P2          ------------------------------------------------------------
PKD1P5          ------------------------------------------------------------
PKD1            GCCACTGGCCCCAGCAGGACACCCCTTCGGGCCAAGAACAAGGTCCACCCCAGCAGCACT
PKD1P4          ------------------------------------------------------------
PKD1P1          ------------------------------------------------------------
PKD1P3          ------------------------------------------------------------
PKD1P6          ------------------------------------------------------------

PKD1P2          ------------------------------------------------------------
PKD1P5          ------------------------------------------------------------
PKD1            TAGTCCTCCTTCCTGGCGGGGGTGGGCCGTGGAGTCGGAGTGGACACCGCTCAGTATTAC
PKD1P4          ------------------------------------------------------------
PKD1P1          ------------------------------------------------------------
PKD1P3          ------------------------------------------------------------
PKD1P6          ------------------------------------------------------------

PKD1P2          ------------------------------------------------------------
PKD1P5          ------------------------------------------------------------
PKD1            TTTCTGCCGCTGTCAAGGCCGAGGGCCAGGCAGAATGGCTGCACGTAGGTTCCCCAGAGA
PKD1P4          ------------------------------------------------------------
PKD1P1          ------------------------------------------------------------
PKD1P3          ------------------------------------------------------------
PKD1P6          ------------------------------------------------------------

PKD1P2          ------------------------------------------------------------
PKD1P5          ------------------------------------------------------------
PKD1            GCAGGCAGGGGCATCTGTCTGTCTGTGGGCTTCAGCACTTTAAAGAGGCTGTGTGGCCAA
PKD1P4          ------------------------------------------------------------
PKD1P1          ------------------------------------------------------------
PKD1P3          ------------------------------------------------------------
PKD1P6          ------------------------------------------------------------

PKD1P2          ------------------------------------------------------------
```

```
PKD1P5          ------------------------------------------------------------
PKD1            CCAGGACCCAGGGTCCCCTCCCCAGCTCCCTTGGGAAGGACACAGCAGTATTGGACGGTT
PKD1P4          ------------------------------------------------------------
PKD1P1          ------------------------------------------------------------
PKD1P3          ------------------------------------------------------------
PKD1P6          ------------------------------------------------------------

PKD1P2          ------------------------------------------------------------
PKD1P5          ------------------------------------------------------------
PKD1            TCTAGCCTCTGAGATGCTAATTTATTTCCCCGAGTCCTCAGGTACAGCGGGCTGTGCCCG
PKD1P4          ------------------------------------------------------------
PKD1P1          ------------------------------------------------------------
PKD1P3          ------------------------------------------------------------
PKD1P6          ------------------------------------------------------------

PKD1P2          ------------------------------------------------------------
PKD1P5          ------------------------------------------------------------
PKD1            GCCCCACCCCCTGGGCAGATGTCCCCCACTGCTAAGGCTGCTGGCTTCAGGGAGGGTTAG
PKD1P4          ------------------------------------------------------------
PKD1P1          ------------------------------------------------------------
PKD1P3          ------------------------------------------------------------
PKD1P6          ------------------------------------------------------------

PKD1P2          ------------------------------------------------------------
PKD1P5          ------------------------------------------------------------
PKD1            CCTGCACCGCCGCCACCCTGCCCCTAAGTTATTACCTCTCCAGTTCCTACCGTACTCCCT
PKD1P4          ------------------------------------------------------------
PKD1P1          ------------------------------------------------------------
PKD1P3          ------------------------------------------------------------
PKD1P6          ------------------------------------------------------------

PKD1P2          ------------------------------------------------------------
PKD1P5          ------------------------------------------------------------
PKD1            GCACCGTCTCACTGTGTGTCTCGTGTCAGTAATTTATATGGTGTTAAAATGTGTATATTT
PKD1P4          ------------------------------------------------------------
PKD1P1          ------------------------------------------------------------
PKD1P3          ------------------------------------------------------------
PKD1P6          ------------------------------------------------------------

PKD1P2          ------------------------------------------------------------
PKD1P5          ------------------------------------------------------------
PKD1            TTGTATGTCACTATTTTCACTAGGGCTGAGGGGCCTGCGCCCAGAGCTGGCCTCCCCCAA
PKD1P4          ------------------------------------------------------------
PKD1P1          ------------------------------------------------------------
PKD1P3          ------------------------------------------------------------
PKD1P6          ------------------------------------------------------------

PKD1P2          ------------------------------------------------------------
PKD1P5          ------------------------------------------------------------
PKD1            CACCTGCTGCGCTTGGTAGGTGTGGTGGCGTTATGGCAGCCCGGCTGCTGCTTGGATGCG
PKD1P4          ------------------------------------------------------------
PKD1P1          ------------------------------------------------------------
PKD1P3          ------------------------------------------------------------
PKD1P6          ------------------------------------------------------------

PKD1P2          ------------------------------------------------------------
PKD1P5          ------------------------------------------------------------
PKD1            AGCTTGGCCTTGGGCCGGTGCTGGGGGCACAGCTGTCTGCCAGGCACTCTCATCACCCCA
PKD1P4          ------------------------------------------------------------
PKD1P1          ------------------------------------------------------------
PKD1P3          ------------------------------------------------------------
PKD1P6          ------------------------------------------------------------

PKD1P2          ------------------------------------------------------------
PKD1P5          ------------------------------------------------------------
PKD1            GAGGCCTTGTCATCCTCCCTTGCCCCAGGCCAGGTAGCAAGAGAGCAGCGCCCAGGCCTG
PKD1P4          ------------------------------------------------------------
PKD1P1          ------------------------------------------------------------
PKD1P3          ------------------------------------------------------------
PKD1P6          ------------------------------------------------------------

PKD1P2          ------------------------------------------------------------
PKD1P5          ------------------------------------------------------------
PKD1            CTGGCATCAGGTCTGGGCAAGTAGCAGGACTAGGCATGTCAGAGGACCCCAGGGTGGTTA
PKD1P4          ------------------------------------------------------------
PKD1P1          ------------------------------------------------------------
PKD1P3          ------------------------------------------------------------
PKD1P6          ------------------------------------------------------------

PKD1P2          ------------------------------------------------------------
PKD1P5          ------------------------------------------------------------
PKD1            GAGGAAAAGACTCCTCCTGGGGGCTGGCTCCCAGGGTGGAGGAAGGTGACTGTGTGTGTG
PKD1P4          ------------------------------------------------------------
PKD1P1          ------------------------------------------------------------
PKD1P3          ------------------------------------------------------------
PKD1P6          ------------------------------------------------------------
```

```
PKD1P2          ------------------------------------------------------------
PKD1P5          ------------------------------------------------------------
PKD1            TGTGTGTGCGCGCGCGCACGCGCGAGTGTGCTGTATGGCCCAGGCAGCCTCAAGGCCCTC
PKD1P4          ------------------------------------------------------------
PKD1P1          ------------------------------------------------------------
PKD1P3          ------------------------------------------------------------
PKD1P6          ------------------------------------------------------------

PKD1P2          ------------------------------------------------------------
PKD1P5          ------------------------------------------------------------
PKD1            GGAGCTGGCTGTGCCTGCTTCTGTGTACCACTTCTGTGGGCATGGCCGCTTCTAGAGCCT
PKD1P4          ------------------------------------------------------------
PKD1P1          ------------------------------------------------------------
PKD1P3          ------------------------------------------------------------
PKD1P6          ------------------------------------------------------------

PKD1P2          ------------------------------------------------------------
PKD1P5          ------------------------------------------------------------
PKD1            CGACACCCCCCCAACCCCCGCACCAAGCAGACAAAGTCAATAAAAGAGCTGTCTGACTGC
PKD1P4          ------------------------------------------------------------
PKD1P1          ------------------------------------------------------------
PKD1P3          ------------------------------------------------------------
PKD1P6          ------------------------------------------------------------

PKD1P2          --
PKD1P5          --
PKD1            AA
PKD1P4          --
PKD1P1          --
PKD1P3          --
PKD1P6          --
```
