# Supplementary material for: Nephroplex: a kidney-focused NGS panel highlights the challenges of PKD1 sequencing and identifies a founder BBS4 mutation
Source: J Nephrol. 2021 May 8;34(6):1855–74. doi: 10.1007/s40620-021-01048-4 (PMC8610957; doi:10.1007/s40620-021-01048-4)
Supplement: Supplementary file 2 — Supplementary file2 (DOCX 1389 KB) [file 40620_2021_1048_MOESM2_ESM.docx]

| **GENE** | **Associated disease** | **GENE** | **Associated disease** | **GENE** | **Associated disease** |
| --- | --- | --- | --- | --- | --- |
| ***ACTN4*** | Glomerulopathy | ***CYP17A1*** | Other | ***PKHD1*** | Ciliopathy |
| ***AGXT*** | Metabolic Nephropathy | ***CYP21A2*** | Other | ***PLCE*** | Glomerulopathy |
| ***AHI1*** | Ciliopathy | ***DGKE*** | Glomerulopathy | ***PTPRO*** | Glomerulopathy |
| ***APOA1*** | Glomerulopathy | ***EGF*** | Tubulopathy | ***REN*** | CAKUT |
| ***APOL1*** | Glomerulopathy | ***EHHADH*** | Tubulopathy | ***SCNN1B*** | Tubulopathy |
| ***AQP2*** | Tubulopathy | ***FAN1*** | Ciliopathy | ***SCNN1G*** | Tubulopathy |
| ***ARL6*** | Ciliopathy | ***FGF23*** | Tubulopathy | ***SDCCAG8*** | Ciliopathy |
| ***ARL13B*** | Ciliopathy | ***FN1*** | Glomerulopathy | ***SLC2A2*** | Metabolic Nephropathy |
| ***ATP6V0A4*** | Tubulopathy | ***FXYD2*** | Tubulopathy | ***SLC2A9*** | Tubulopathy |
| ***ATP6V1B1*** | Tubulopathy | ***GATA3*** | CAKUT | ***SLC3A1*** | Tubulopathy |
| ***AVP*** | Other | ***GLA*** | Metabolic Nephropathy | ***SLC4A1*** | Tubulopathy |
| ***AVPR2*** | Tubulopathy | ***GLIS2*** | Ciliopathy | ***SLC4A4*** | Tubulopathy |
| ***BBS1*** | Ciliopathy | ***HPRT1*** | Tubulopathy | ***SLC5A2*** | Tubulopathy |
| ***BBS2*** | Ciliopathy | ***HSD11B2*** | Tubulopathy | ***SLC7A9*** | Tubulopathy |
| ***BBS4*** | Ciliopathy | ***INF2*** | Glomerulopathy | ***SLC9A3R1*** | Metabolic Nephropathy |
| ***BBS5*** | Ciliopathy | ***INPP5E*** | Ciliopathy | ***SLC12A1*** | Tubulopathy |
| ***BBS7*** | Ciliopathy | ***INVS*** | Ciliopathy | ***SLC22A3*** | Other |
| ***BBS9*** | Ciliopathy | ***IQCB1*** | Ciliopathy | ***SLC22A12*** | Tubulopathy |
| ***BBS10*** | Ciliopathy | ***KCNJ1*** | Tubulopathy | ***SLC26A4*** | Tubulopathy |
| ***BBS12*** | Ciliopathy | ***KCNJ5*** | Other | ***SLC26A6*** | Tubulopathy |
| ***BSND*** | Tubulopathy | ***KLHL3*** | Tubulopathy | ***SLC34A1*** | Metabolic Nephropathy |
| ***C2ORF86*** | Ciliopathy | ***LAMB2*** | Glomerulopathy | ***SOX17*** | CAKUT |
| ***CA2*** | Tubulopathy | ***MKKS*** | Ciliopathy | ***TMEM216*** | Ciliopathy |
| ***CASR*** | Tubulopathy | ***MKS1*** | Ciliopathy | ***TMEM237*** | Ciliopathy |
| ***CD2AP*** | Glomerulopathy | ***MUC1*** | Ciliopathy | ***TRIM32*** | Ciliopathy |
| ***CEP41*** | Ciliopathy | ***NEK8*** | Ciliopathy | ***TRPC6*** | Glomerulopathy |
| ***CEP164*** | Ciliopathy | ***NPHP1*** | Ciliopathy | ***TRPV6*** | Other |
| ***CEP290*** | Ciliopathy | ***NPHP3*** | Ciliopathy | ***TSC1*** | Ciliopathy |
| ***CLCN5*** | Tubulopathy | ***NPHP4*** | Ciliopathy | ***TSC2*** | Ciliopathy |
| ***CLCNKA*** | Tubulopathy | ***NPHS1*** | Glomerulopathy | ***TTC8*** | Ciliopathy |
| ***CLCNKB*** | Tubulopathy | ***NPHS2*** | Glomerulopathy | ***UMOD*** | Ciliopathy |
| ***CLDN16*** | Tubulopathy | ***NR3C1*** | Other | ***UPK3A*** | CAKUT |
| ***CLDN19*** | Tubulopathy | ***NR3C2*** | Tubulopathy | ***VHL*** | Ciliopathy |
| ***CNNM2*** | Tubulopathy | ***OCRL*** | Tubulopathy | ***WNK1*** | Tubulopathy |
| ***COL4A3*** | Glomerulopathy | ***OFD1*** | Ciliopathy | ***WNK4*** | Tubulopathy |
| ***COL4A4*** | Glomerulopathy | ***PAX2*** | CAKUT | ***XPNPEP3*** | Ciliopathy |
| ***COL4A5*** | Glomerulopathy | ***PHEX*** | Tubulopathy | ***ZNF423*** | Ciliopathy |
| ***CTNS*** | Tubulopathy | ***PKD1*** | Ciliopathy |  |  |
| ***CUL3*** | Tubulopathy | ***PKD2*** | Ciliopathy |  |  |

**Supplemental Table 1. Selected genes for Nephroplex.**

Abbreviations: CAKUT=congenital anomalies of the kidney and the urinary tract.

| **Patient ID** | **Sex** | **Phenotype** | **gene** | **HumanGRCh37/hg19** | **Genetic Variant** | **Exon** | **protein variant** | **ExAC** | **gnomAD** | **CLINVAR** | **VARSOME** | **MAYO** | **Note** |
| --- | --- | --- | --- | --- | --- | --- | --- | --- | --- | --- | --- | --- | --- |
| K7 | F | Hypercalciuria | *PKD1* | chr16:2159202 | NM_000296:c.5964_5965delTG | 15 | p.R1990Efs*59 | 0 | 0 | Not reported | Pathogenic | Not reported | possible contamination |
| K8 | M | Hypercalciuria | *PKD1* | chr16:2147491 | NM_000296:c.10231C>T | 33 | p.P3411S | 0.001072 | 0.000824 | Likely Benign​ | Likely Benign | Likely Neutral | possible contamination |
|  |  |  | *PKD1* | chr16:2149896 | NM_000296:c.9889G>A | 29 | p.V3297M | 0.0002037 | 0.000212 | Uncertain Significance | Likely Benign | Not reported | possible contamination |
|  |  |  | *PKD1* | chr16:2161563 | NM_000296:c.3605C>T | 15 | p.A1202V | 0.000 | 0.0000425 | Not reported | Uncertain Significance/Likely Benign | Not reported | possible contamination |
| K12 | M | Diabetes Insipidus + | *PKD1* | chr16:2141018 | NM_000296:c.11867G>A | 43 | p.G3956D | 0.0009940 | 0.000631 | Not reported | Likely Benign | Likely Neutral | possible contamination |
|  |  | Nephrocalcinosis |  |  |  |  |  |  |  |  |  |  |  |
| K17 | F | Gitelman syndrome | *PKD1* | chr16:2150517 | NM_000296:c.9448G>A | 27 | p.G3150S | 0 | 0.0000081 | Not reported | Likely Pathogenic | Not reported | possible contamination |
| K20 | M | Hypertension/Hyperaldost. | *PKD2* | chr4:88989089 | NM_000297:c.2398A>C | 13 | p.M800L | 0.004812 | 0.00487 | Likely Benign​ | Benign | Likely Neutral | possible contamination |
| K26 | M | Bardet-Biedl syndrome | *PKD1* | chr16:2164535 | NM_000296:c.2489C>T | 11 | p.A830V | 0.00005214 | 0.0000137 | Not reported | Uncertain Significance | Not reported | possible contamination |
| K30 | F | Bardet-Biedl syndrome | *PKD1* | chr16:2157957 | NM_000296:c.6992C>G | 16 | p.A2331G | 0 | 0.000109 | Not reported | Likely Benign | Not reported | possible contamination |
| K39 | F | Gitelman syndrome | *PKD2* | chr4:88967919 | NM_000297:c.1445T>G | 6 | p.F482C | 0.002117 | 0.00204 | Likely Benign​ | Benign | Likely Neutral | possible contamination |
| K41 | M | Bardet-Biedl syndrome | *PKD1* | chr16:2159385 | NM_000296:c.5783C>G | 15 | p.P1928R | 0.0003906 | 0.000373 | Not reported | Likely Benign | Likely Neutral | possible contamination |
| K42 | F | Alport syndrome | *PKD1* | chr16:2159805 | NM_000296:c.5363G>A | 15 | p.G1788D | 0.0003973 | 0.000355 | Not reported | Likely Benign | Likely Neutral | possible contamination |
|  |  |  | *PKD2* | chr4:88996055 | NM_000297:c.2614C>G | 14 | p.R872G | 0.00004121 | 0.0000358 | Not reported | Uncertain Significance | Not reported | possible contamination |
| K45 | M | Bardet-Biedl syndrome | *PKD1* | chr16:2161738 | NM_000296:c.3430G>A | 15 | p.V1144I | 0.0009743 | 0.000319 | Not reported | Likely Benign | Likely Neutral | possible contamination |
| K58 | F | Bardet-Biedl syndrome | *PKD1* | chr16:2159557 | NM_000296:c.5611G>A | 15 | p.A1871T | 0.004631 | 0.00389 | Benign/Likely benign​ | Benign | Likely Neutral | possible contamination |
| K61 | F | CAKUT | *PKD2* | chr4:88973149 | NM_000297:c.1555G>A | 7 | p.V519M | 0.00006700 | 0.0000921 | Uncertain Significance | Likely Benign | Indeterminate | possible contamination |
| K68 | F | Renal Glucosuria | *PKD2* | chr4:88967918 | NM_000297:c.1444T>G | 6 | p.F482V | 0.00003295 | 0.0000318 | Uncertain Significance | Likely Benign | Indeterminate | possible contamination |
| K78 | M | Bartter/Gitelman syndrome | *PKD1* | chr16:2160904 | NM_000296:c.4264G>A | 15 | p.A1422T | 0.006014 | 0.00591 | Benign/Likely benign​ | Benign | Likely Neutral | possible contamination |
| K86 | F | Renal Glucosuria | *PKD1* | chr16:2159557 | NM_000296:c.5611G>A | 15 | p.A1871T | 0.004631 | 0.00389 | Benign/Likely benign​ | Benign | Likely Neutral | possible contamination |
| K87 | F | Bardet-Biedl syndrome | *PKD1* | chr16:2140294 | NM_000296:c.12433G>A | 45 | p.V4145I | 0.004847 | 0.00439 | Benign/Likely benign​ | Benign | Likely Neutral | possible contamination |
|  |  |  | *PKD1* | chr16:2164302 | NM_000296:c.2722G>A | 11 | p.V908M | 0.00007801 | 0.0000805 | Not reported | Likely Benign | Not reported | possible contamination |
|  |  |  | *PKD1* | chr16:2154549 | NM_000296:c.8111C>T | 22 | p.A2704V | 0.003471 | 0.00142 | Benign/Likely benign​ | Benign | Likely Neutral | possible contamination |
| K101 | F | Multicystic kidney (CAKUT) | *PKD1* | chr16:2140752 | NM_000296:c.12058C>G | 44 | p.R4020G | 0.00002561 | 0.0000203 | Not reported | Likely Benign | Not reported | likely benign |
|  |  |  | *PKD1* | chr16:2160904 | NM_000296:c.4264G>A | 15 | p.A1422T | 0.006014 | 0.00591 | Benign/Likely benign​ | Benign | Likely Neutral | possible contamination |
|  |  |  | *PKD1* | chr16:2165612 | NM_000296:c.1864G>A | 10 | p.G622S | 0.00001616 | 0.00000896 | Not reported | Uncertain Significance | Not reported | possible contamination |
| K102 | F | Acidosi tubulare | *PKD1* | chr16:2160904 | NM_000296:c.4264G>A | 15 | p.A1422T | 0.006014 | 0.00591 | Benign/Likely benign​ | Benign | Likely Neutral | possible contamination |
| K106 | M | Gitelman syndrome | *PKD1* | chr16:2160904 | NM_000296:c.4264G>A | 15 | p.A1422T | 0.006014 | 0.00591 | Benign/Likely benign​ | Benign | Likely Neutral | possible contamination |
| K109 | M | Alport syndrome | *PKD2* | chr4:88996137 | NM_000297:c.2670+26C>T | intron | spl | 0.00003311 | 0.00002 | Not reported | Likely Benign | Not reported | benign |
| K128 | M | Bardet-Biedl syndrome | *PKD1* | chr16:2141167 | NM_001009944:c.11718C>G | 43 | p.C3906W | 0 | 0 | Not reported | Uncertain Significance | Not reported | likely benign |

**Supplemental Table 2**. List of *PKD1-2* variants in the cohort of non-cystic individuals.


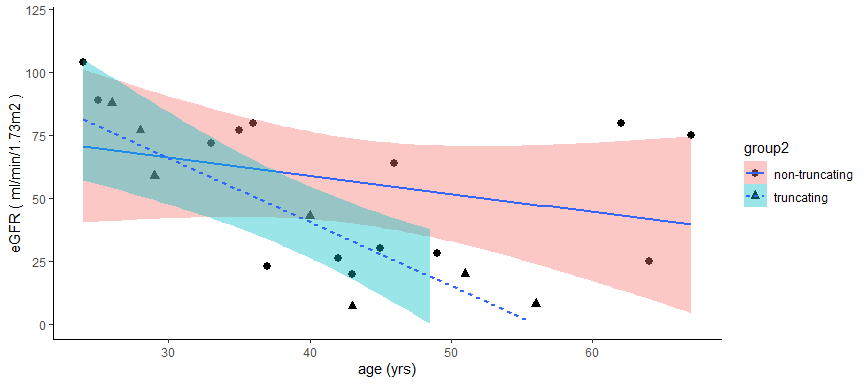

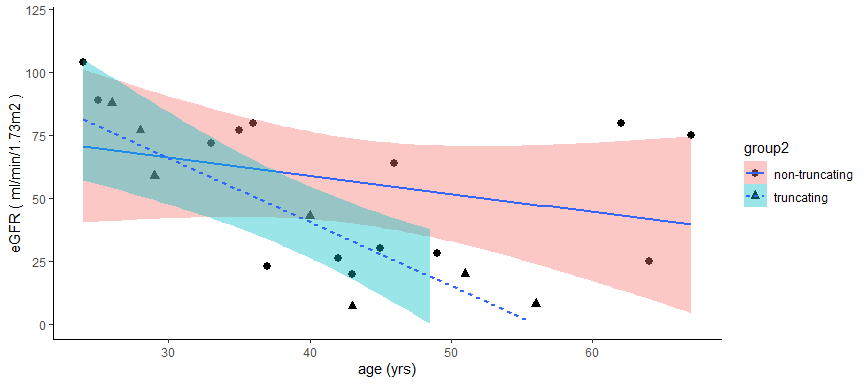

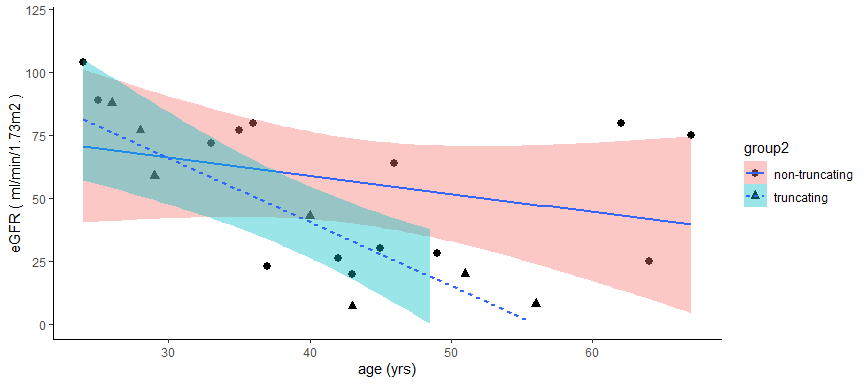


All other ADPKD Patients

Patients with truncating *PKD1* mutations

**Supplemental Figure 1. Genotype-phenotype correlation in ADPKD patients**: Patients with truncating *PKD1* mutations showed a trend toward a most severe estimated glomerular filtration rate (eGFR) decline over years than others (patients with missense *PKD1* variants, any *PKD2* variants and no detected mutations).
